# Antitumor activity and expression profiles of genes induced by sulforaphane in human melanoma cells

**DOI:** 10.1007/s00394-017-1527-7

**Published:** 2017-09-01

**Authors:** Paola Arcidiacono, Francesco Ragonese, Anna Stabile, Alessandra Pistilli, Ekaterina Kuligina, Mario Rende, Ugo Bottoni, Stefano Calvieri, Andrea Crisanti, Roberta Spaccapelo

**Affiliations:** 1 Department of Life Sciences, Imperial College London, London SW7 2AZ, United KingdomDepartment of Experimental Medicine, University of Perugia, Piazza Lucio Severi, 06132 Perugia, Italy; 2 Dermatology Clinic, Department of Internal Medicine and Medical Specialties, University of Rome, Rome, Italy; 3 Department of Surgery and Biomedical Sciences, University of Perugia, 06132 Perugia, Italy; 4 Present Address: N.N. Petrov Institute of Oncology, Saint Petersburg, 197758 Russia; 5 University Magna Graecia, Catanzaro, Italy; 6 Department of Life Sciences, Imperial College London, London, SW7 2AZ United Kingdom

**Keywords:** Melanoma, Melanocytes, Sulforaphane, Apoptosis, Transcriptome, RNA-Seq

## Abstract

**Purpose:**

Human melanoma is a highly aggressive incurable cancer due to intrinsic cellular resistance to apoptosis, reprogramming, proliferation and survival during tumour progression. Sulforaphane (SFN), an isothiocyanate found in cruciferous vegetables, plays a role in carcinogenesis in many cancer types. However, the cytotoxic molecular mechanisms and gene expression profiles promoted by SFN in human melanoma remain unknown.

**Methods:**

Three different cell lines were used: two human melanoma A375 and 501MEL and human epidermal melanocytes (HEMa). Cell viability and proliferation, cell cycle analysis, cell migration and invasion and protein expression and phosphorylation status of Akt and p53 upon SFN treatment were determined. RNA-seq of A375 was performed at different time points after SFN treatment.

**Results:**

We demonstrated that SFN strongly decreased cell viability and proliferation, induced G_2_/M cell cycle arrest, promoted apoptosis through the activation of caspases 3, 8, 9 and hampered migration and invasion abilities in the melanoma cell lines. Remarkably, HEMa cells were not affected by SFN treatment. Transcriptomic analysis revealed regulation of genes involved in response to stress, apoptosis/cell death and metabolic processes. SFN upregulated the expression of pro-apoptotic genes, such as *p53*, *BAX*, *PUMA*, *FAS* and *MDM2*; promoted cell cycle inhibition and growth arrest by upregulating *EGR1*, *GADD45B*, *ATF3* and *CDKN1A*; and simultaneously acted as a potent inhibitor of genotoxicity by launching the stress-inducible protein network (HMOX1, HSPA1A, HSPA6, SOD1).

**Conclusion:**

Overall, the data show that SFN cytotoxicity in melanoma derives from complex and concurrent mechanisms during carcinogenesis, which makes it a promising cancer prevention agent.

**Electronic supplementary material:**

The online version of this article (doi:10.1007/s00394-017-1527-7) contains supplementary material, which is available to authorized users.

## Introduction

Melanoma continues to be the skin disease causing the highest mortality due to its propensity to metastasize. In fact, it is responsible for more than 75% of skin cancer deaths. Epidemiological studies have shown that the incidence of melanoma increases at a faster rate than that of any other cancer worldwide [1, 2]. Unsatisfactory results with single-agent or combination chemotherapy schemes underscore the need for the application of different strategies to cure melanoma. Treatment options have rapidly expanded in the past 5 years with the introduction of targeted therapy (BRAF and MEK inhibitors) and immune checkpoint blockades [3–5]. Interestingly, many naturally occurring dietary compounds found in fruits and vegetables consumed daily (e.g., curcumin, indole-3-carbinol, brassinin, sulforaphane (SFN), epigallocatechin-3-gallate, lycopene and quercetin) have been found to inhibit one or more pathways that contribute to malignant transformation and possess cancer-preventive properties against different types of tumours, including melanoma [6, 7]. SFN (*R*-1-isothiocyanato-4-methylsulfinyl butane), an isothiocyanate found especially in broccoli sprouts, Chinese kale, cabbage and watercress, can prevent or delay preneoplastic lesions as well as act as a therapeutic agent in tumour cell cultures and carcinogen-induced and genetic animal cancer models [8–10]. SFN induces phase II carcinogen detoxification enzymes, such as NAD(P)H:quinone oxidoreductase I [11] and heme oxygenase-1 (HMOX-1), via the ARE–NRF2 pathway [12], thereby allowing a different array of electrophilic and oxidative toxicants to be eliminated before they damage critical cellular macromolecules [13, 14]. Studies have shown that SFN induces apoptosis and inhibits the progression and metastasis of many cancers [15–17]. Despite a growing number of studies describing the chemopreventive and chemotherapeutic properties of SFN in cancer [18–20], little is known about the molecular mechanisms underlying the antitumour effects of SFN in human melanoma cells. Some previous reports have shown that SFN could be effective in treating melanoma, but virtually all promising results have thus far been obtained from animal studies. Specifically, SFN was shown to inhibit the metastasis of B16F-10 mouse melanoma cells both in vivo and in vitro [21]. In a murine model, SFN was shown to induce a significant reduction in the expression of cell proliferation markers (metalloproteinases 2 and 9), leading to an increase in the survival rate of animals bearing metastatic tumours, probably via stimulating a cell-mediated immune response [22, 23]. Moreover, SFN-induced apoptosis in B16F-10 has been shown to be associated with the activation of caspases 3 and 9, BAX and p53 and the downregulation of NF-kB [24].

In this study, we analysed the tumour cytotoxic effects of SFN on human primary melanoma cells (A375), human metastatic melanoma cells (501MEL) and control human epidermal melanocytes (HEMa) using multiple approaches. We also aimed to decipher the A375 cell transcriptome following exposure to SFN using RNA sequencing (RNA-Seq) technology. To the best of our knowledge, the present study is the first to demonstrate the effects of SFN on HEMa cells and on the expression profiles of genes involved in SFN-induced apoptosis, cell cycle arrest, migration and invasion in melanoma.

## Materials and methods

### Reagents

DL-SFN, dimethyl sulfoxide (DMSO), propidium iodide (PI), Ponceau S and collagenase were purchased from Sigma-Aldrich. Trypan blue, Dulbecco’s modified Eagle medium (DMEM), Roswell Park Memorial Institute (RPMI) 1640, l-glutamine 100×, foetal bovine serum (FBS), penicillin–streptomycin, 0.25% trypsin–EDTA, Medium 254, human melanocyte growth supplement-2 PMA-Free (HMGS-2), RNAse-A, DEPC–water, collagen I bovine, Hank’s balanced salt solution (HBSS), trypsin neutralizer solution and 0.025% trypsin/EDTA solution were obtained from Gibco Life Technologies. Other chemical supplements and compounds were purchased from several companies as follows: CellTiter-Glo luminescent cell viability assay (Promega), turbo DNA free (Ambion, Life Technologies), FITC Annexin V apoptosis detection kit I (BD Pharmingen), TRIzol reagent (Ambion, Life Technologies), methylene blue (Merck), BCA protein assay kit and NP-40 (Pierce, Thermo scientific), ECL Prime Western blotting detection reagents (Amersham), stripping buffer stripAblot and Tween 20 (EuroClone), complete protease inhibitor cocktail (Roche), PageRule prestained protein ladder (Fermentas, part of Thermo Fisher Scientific), superscript III Reverse Transcriptase (Life Technologies) and iQ™ SYBR^®^ green supermix (Bio-Rad).

### Cell lines and treatments

A375 is a primary human malignant melanoma cell line that was obtained from American Type Culture Collection (ATCC). The cells were maintained in DMEM medium containing 10% FBS, 1% l-glutamine and 1% penicillin–streptomycin (10,000 units/ml). The 501MEL cell line was obtained from surgically removed tumours from patients with melanoma at the National Cancer Institute in Milan, Italy, and they were maintained in RPMI 1640 medium supplemented with 10% FBS, 1% l-glutamine (200 mM) and 1% penicillin–streptomycin (10,000 units/ml). Human epidermal melanocyte (HEMa) is a cell line isolated from lightly pigmented (LP) adult skin purchased from Invitrogen (Life Technologies). The cells were propagated in Medium 254 supplemented with HMGS-2 and incubated at 37 °C with 5% CO_2_ until they reached approximately 70% confluence. They were then treated with different concentrations of SFN dissolved in dimethyl sulfoxide (DMSO). The same DMSO concentration used to dilute the SFN was utilized as a negative control (untreated).

### Cell viability and morphology analysis

Cells were seeded at a density of 3 × 10^5^ cells/well onto six-well plates, and after 24 h treated with SFN at final concentrations of 1, 2 and 5 μg/ml. The control cells were treated with the same concentration of DMSO (0.001%). The cell lines were photographed under a phase-contrast microscope at 10× magnification (Nikon Eclipse) to observe morphological changes. For the viability assay, A375 and 501MEL cells were plated at a density of 3 × 10^5^ cells/well and treated with SFN for 24 and 48 h. Dead cells were stained with 0.4% trypan blue dye. In addition, cell viability was evaluated using the Cell Titer-Glo assay. The cells were plated at a density of 5 × 10^3^ cells/well in 100 μL of medium on 96-well white plates (Cellstar, Greiner) and treated with different concentrations of SFN for 24 and 48 h. The cells were subsequently lysed using Cell Titer-Glo luminescent reagent following the manufacturer’s protocol, and the results are expressed as a percentage based on the ratio of the absorbance of treated cells to that of the control cells (100%).

### Cell cycle analysis

Cells were plated at a density of 3 × 10^5^ cells/well on six-well plates and treated with SFN for 24 and 48 h. After incubation, the cells were trypsinized, washed with ice-cold phosphate-buffered saline (PBS) and fixed in 70% ethanol overnight. Next, the cells were re-suspended in PBS containing RNAse-A (30 mg/mL) and incubated at 4 °C for 5 min. After FACS buffer (PBS + 2% FBS) and PI (1 mg/mL) were added, the cells were incubated at 4 °C for 30 min and then immediately analysed for DNA content using the FACSCalibur flow cytometer with CellQuest software (Becton–Dickinson). In total, 20,000 events per sample were recorded, and experiments were performed in triplicate. Apoptotic cells with hypodiploid DNA content were measured by quantifying the sub-G_1_ peak in the cell cycle pattern.

### Apoptotic cell death assay

Cells were plated at a density of 3 × 10^5^ cells/well on six-well plates, and 24 h later treated with SFN for 24 and 48 h. Floating cells were collected, combined with the trypsinized adherent cells and stained with Annexin V and PI according to the manufacturer’s recommended protocol. The samples were analysed by FACS within 1 h. For this assay, 20,000 events were counted. The analyses were performed in triplicate.

### Western blotting analysis

Cells were washed with PBS and then lysed with lysis buffer [1 M Tris, 2.5 M NaCl, 10% glycerol, 0.5 M glycerophosphate, 1% Tween 20, 0.5% NP-40 and a complete protease inhibitor tablet (Roche)] for 20 min on ice. The cell lysates were separated by 10 or 12% denaturing SDS-PAGE. The proteins were then transferred to nitrocellulose membranes (Whatman), blocked with PBS containing 5% milk and 0.05% Tween and incubated with specific primary antibodies overnight. After being washed with PBS containing 0.05% Tween, the blots were incubated with peroxidase-conjugated secondary antibodies labelled with horseradish peroxidase (HRP) (Sigma, Italy) and developed using ECL according to the manufacturer’s instructions. Rabbit polyclonal anti-GAPDH (ab 9485) and anti-procaspase-9 (ab 32068) were purchased from Abcam (Italy) and diluted 1:500. Rabbit anti-PARP (9542) and anti-p53 (Ser15-9286) were purchased from Cell Signaling Technology (Italy) and diluted 1:500. Anti-cleaved caspase-3 (9661), also purchased from Cell Signaling Technology, was diluted 1:1000. Rabbit anti-Bcl2 (ab 59348) and rabbit polyclonal anti-cyclin B1 (4138), purchased from Santa Cruz Biotechnology (Italy), were diluted 1:500. Rabbit anti-procaspase-8 (ab 49853) was purchased from Sigma and diluted 1:1000.

### Scratch wound healing assay

A375 cells were seeded in six-well plates at a concentration of 3 × 10^5^ cells/well in complete medium and incubated for 24 h. After 24 h, the cells were treated with mitomycin (15 µg/ml, Sigma) to prevent cell proliferation for 45 min at 37 °C. Next, the medium was removed, and a vertical scratch was created in the centre of the well using a sterile tip. The cells were subsequently washed with PBS and treated with 2 µg/ml SFN. The groove was monitored and photographed both immediately and 24 h after the scratch was created using a phase-contrast microscope (Evos, Zeiss) with 4× magnification. The migration was evaluated as the residual area of the groove, and three different fields were counted for each condition. The wound area was calculated by tracing a line along the border of the wound using ImageJ software, and the percentage of wound closure was calculated using the following equation: [Wound area (0 h) − Wound area (*X* h)] × 100/Wound area (0 h) = %Wound closure.

### Invasion assay with collagen

The collagen matrix was generated from bovine type I collagen at a final concentration of 1 mg/ml according to the manufacturer’s protocol. Collagen was plated immediately onto 24-well plates and then incubated at 37 °C with 95% humidity for 30 min. After collagen polymerization, cells were seeded at a density of 3 × 10^4^ cells/well and treated with 2 µg/ml SFN. After treatment, viability and invasion were evaluated by analysing and counting the cells in the supernatant, the adherent cells harvested using PBS/EDTA (5 min at 37 °C) from the upper collagen surface and the cells remaining in the collagen matrix after the adherent cells were removed. Collagen was fixed with paraformaldehyde (4%), and migrated cell nuclei were stained with blue methylene (1:10). The samples were analysed using a microscope (Olympus BH-2) to count cell numbers (*n* = 6 independent fields for each condition), and cell images were captured with an Olympus U-PMTVC camera. The analyses were performed in triplicate for statistical evaluation.

### Quantitative real-time PCR (qPCR)

cDNA was synthesized using Superscript III Reverse Transcriptase (Sigma) according to the manufacturer’s instructions. qPCR was performed in 96-well plates using iQ™ SYBR Green Supermix (Bio-Rad) and an I-cycler iQ Real-Time PCR instrument (Bio-Rad). Measurements were performed in triplicate with a variability <0.5 Ct. mRNA expression was analysed by normalizing to that of the housekeeping gene GADPH. Primers were designed with PERL primer software using NCBI EntrezGene reference sequences as templates and synthesized by Sigma. The primers used are listed in Supplementary Table S4.

### RNA sequencing (RNA-seq) and data analysis

A375 cells were seeded at a density of 3 × 10^5^ cells/well on six-well plates and treated with SFN for 2, 6, 24 and 48 h. Total RNA was isolated using a standard TRIzol (Life Technologies) extraction protocol. Contaminating DNA was removed by Turbo DNase treatment (Life Technologies), and RNA was purified using the RNeasy Mini Kit (Qiagen). RNA quality was measured by the RNA 6000 Bioanalyzer Nano Kit (Agilent Technologies) according to the manufacturer’s instructions. Only samples with a minimum RIN score of 9.8 were used for further analyses. Double-stranded cDNA libraries were generated from 1 μg of total RNA using a TruSeq RNA Sample Preparation Kit (Illumina) following the manufacturer’s instructions. Libraries were validated using an Agilent High-Sensitivity DNA Kit on an Agilent 2100 Bioanalyzer (Agilent Technologies) and quantified by qPCR using a KAPA Library Quantification Kit (Kapa Biosystems) on a StepOne Real-Time PCR system (Applied Biosystem) at 95 °C (5 min), followed by 35 cycles of 95 °C (30 s) and 60 °C (45 s). After being quantified, the libraries were normalized to a final concentration of 10 nM. The libraries (2 nM) were pooled at final concentrations of 9 and 7 pM and loaded into a HiSeq SR flow cell v3 (Illumina) using a cBot (Illumina) according to the manufacturer’s instructions. The loaded flow cell was sequenced on a HiSeq 1500 platform (Illumina, by PoloGGB) to perform a 100 bp single-read run following the manufacturer’s instructions. The images were processed using the Illumina RTA base calling pipeline, converted in FASTQ using Casava 1.8 software and evaluated for quality using FastQC (v 0.10.1). The reads were trimmed with Trimmomatic (v 0.32) and all reads with >Q20 were selected and aligned with STAR (v 2.3.0) to hg19/GRCh37 in the UCSC Genome Browser. Bam and GTF files were used to create a matrix associating all mapped read numbers to each sample and gene using Htseq. The obtained matrix was processed with the DeSeq R package to select for all differential expressed genes (DEGs) that had a false discovery rate (FDR) <10%. Expression differences were considered significant if their *p* value was <0.05 and their induction (or repression) ratio was ≥1.5. All graphs were produced with R software (v 3.0.0). Functional clustering was performed with DAVID 6.7 and the Web Gene Set Analysis Toolkit (WebGestalt) for enrichment analysis of the differentially expressed genes. DEGs were screened using enrichment analysis based on the hypergeometric distribution WebGestalt algorithm.

### Constructing the protein interaction network

Using the online database Search Tool for the Retrieval of Interacting Genes (STRING) v. 9.1 ([25]; http://string-db.org), interactions between the DEGs were predicted. The interactions include direct (physical) and indirect (functional) associations derived from four sources: genomic context, high-throughput, co-expression and prior knowledge.

### Statistical analysis

All experiments were performed at least three times independently. The data are expressed as the mean ± standard deviation (SD). Data were analysed by the paired two-tailed Student’s *t* test [*p* < 0.05 (*), *p* < 0.01 (**) and *p* < 0.001 (***)]. The Dunnett’s test was used to assess the significance of the viability assay results. All statistical analyses were performed with Prism 5 Software.

## Results

### SFN decreased the viability and induced morphological changes in human melanoma cells

**Fig. 1:**
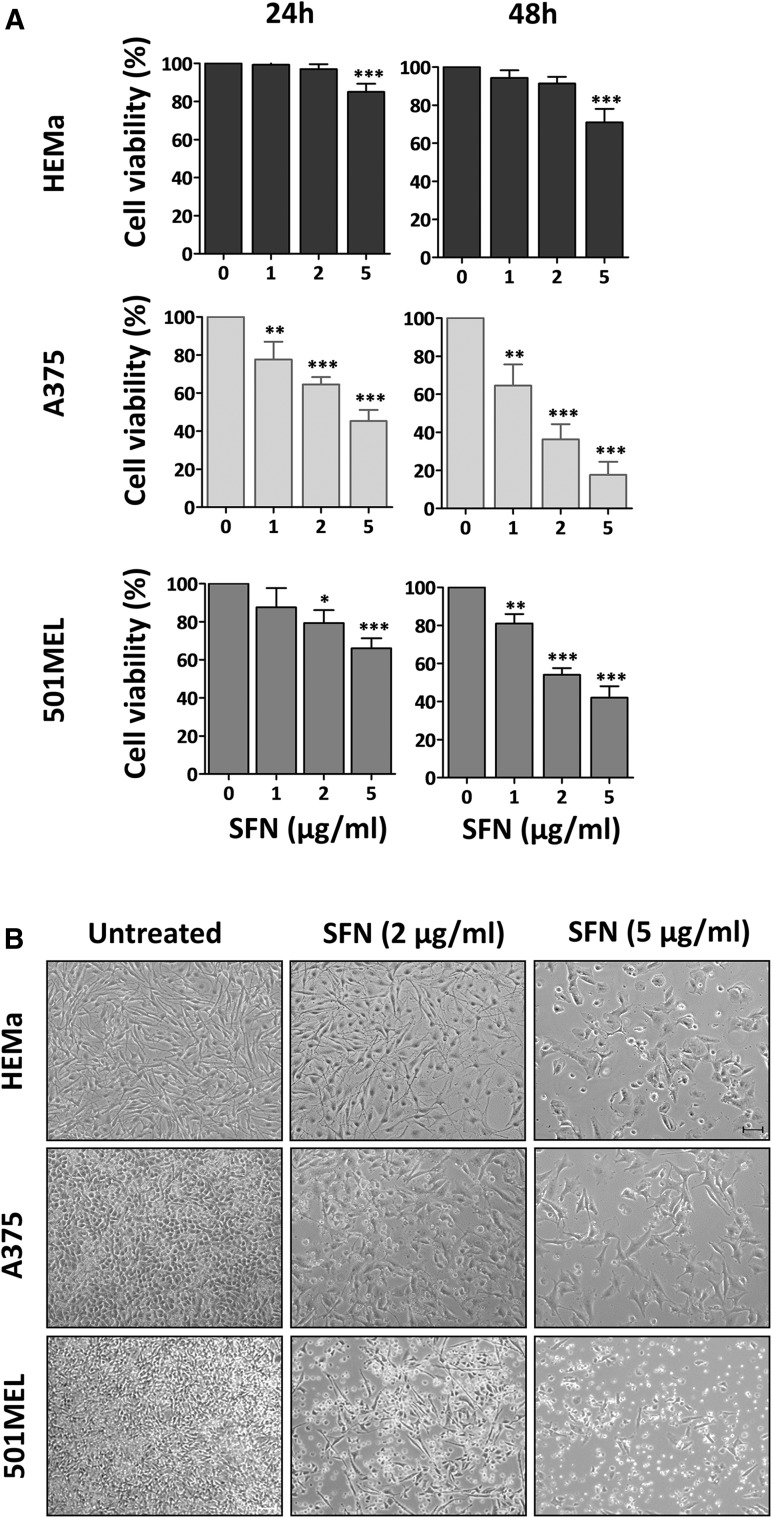
SFN inhibits cell viability and induces morphological changes. **a** HEMa, A375, and 501MEL cells were treated with different doses of SFN for 24 or 48 h. Cell viability was determined by CellTiter-Glo assay. The *bars* represent the mean values of three experiments plus standard deviation; the significance level compared to the control was specified as *p < 0.05, **p < 0.01 and ***p < 0.001 using one-way ANOVA and the Dunnett’s multiple comparison test. **b** Representative phase-contrast microscopy (×10) of cell morphology with or without SFN (2 and 5 μg/ml) at 48 h. *Scale bar* 100 μm

To assess the effects of SFN on cell viability, primary human melanoma A375, metastatic human melanoma 501MEL and HEMa cells were treated with increasing concentrations of SFN (1–5 µg/ml) for 24 and 48 h. SFN treatment significantly reduced the viability of both A375 and 501MEL cells in a dose- and time-dependent manner (Fig. 1a), though A375 cells were more sensitive to SFN treatment than 501MEL cells. In fact, SFN began to significantly reduce the viability of 501MEL cells only at the concentration of 2 µg/ml. After 48 h, 2 µg/ml SFN decreased the viability of the A375 cells by 64% compared to only 46% for the 501MEL cells. As a control, HEMa cells were treated with the same concentrations of SFN, and only the higher dose (5 µg/ml) significantly decreased their viability. These results were also confirmed by cell proliferation analysis using the trypan blue exclusion assay (Supplement Fig. S1a) and phosphorylated AKT (p-AKT) expression analysis (Supplement Fig. S1b). Trypan blue cell counting showed significantly reduced proliferation of both A375 and 501MEL cells at 24 and 48 h post-treatment. Moreover, high levels of p-AKT in both melanoma cell lines were significantly decreased after 24 h of SFN treatment.

To determine cell death due to reduced viability, cell morphology was observed in the confluent monolayer after treatment with 2 and 5 µg/ml SFN. Specifically, the A375 cells displayed increased size, irregular shape and membrane blebbing after 48 h (Fig. 1b). Morphological alterations were also observed in the 501MEL treated cells, as they retracted into a spherical shape and formed suspended clusters. In contrast to melanoma cells, HEMa cells did not exhibit any significant morphological alterations at the 2 µg/ml dose of SFN, and only high concentrations of SFN induced rounded melanocytes, irregular morphology and membrane blebbing. Because 2 µg/ml SFN had no inhibitory effect on HEMa cells but was extremely effective in both melanoma cells lines, this concentration was used for further analysis.

### Sulforaphane induced cell cycle arrest and apoptosis

**Fig. 2:**
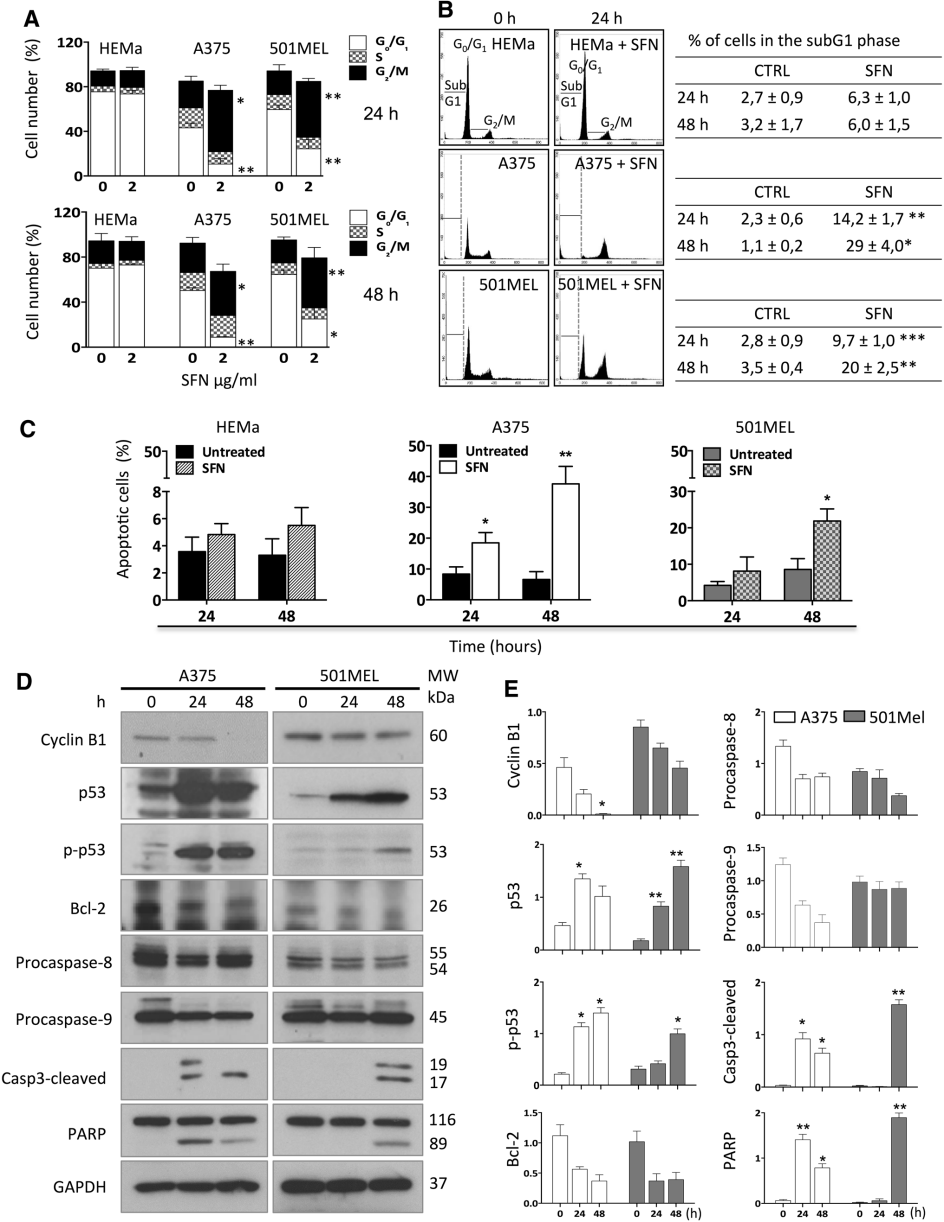
SFN promotes cell cycle arrest and apoptosis. **a** Assessment of DNA content in A375, 501MEL and HEMa cells after 24 or 48 h of treatment with 2 μg/ml SFN. DNA content analysis was carried out by flow cytometry using propidium iodide staining. The percentages of A375, 501MEL and HEMa cells in the G_0_/G_1_, G_2_/M and S phases of the cell cycle with or without 2 μg/ml SFN are shown. **b** Representative plots of the cell cycle analysis histogram by flow cytometry before and after 24 h treatment are reported. The table indicates the percentage of cells with or without 2 µg/ml SFN in the sub-G_1_ phase. **c** Annexin V assay of cells at 24 and 48 h after 2 μg/ml SFN exposure. The graphs represent the mean ± SD of apoptotic (early and late) cells at 24 h post-treatment. **d** Western blot analysis of several proteins involved in the cell cycle and apoptosis in A375 and 501MEL cells after 24 or 48 h exposure to 2 µg/ml SFN. GAPDH was used as loading control. The molecular weight (MW) of the proteins (kDa) is reported. **e** The representative blots show the protein expression levels of the different proteins analysed and the *bar graph* represents the results from the photodensitometric analysis of the bands, using GADPH as an internal control. The data are presented as mean values ± SD of three independent experiments. *p < 0.05, **p < 0.01, ***p < 0.001 using a paired two-tailed Student’s *t* test

To further investigate the inhibitory effects of SFN on cell viability, we analysed cell cycle progression and apoptosis by both flow cytometry and Western blot. SFN exposure changed the cell cycle phase distribution in both melanoma cell lines, and, in agreement with the cell viability data, no changes were observed in the HEMa cells (Fig. 2a). A375 and 501MEL cells treated with SFN significantly accumulated in the G_2_/M phase, as up to 55 and 50% of cells were observed in this phase at 24 h post-treatment, respectively. These numbers shifted down to 40 and 45%, respectively, after 48 h (Fig. 2a). Conversely, the proportion of cells in the G_0_/G_1_ phase was markedly decreased in both cell lines, while the percentage of cells in the S phase remained stable.

To evaluate whether SFN induces apoptosis in human melanoma cell lines, we used several experimental approaches. The assessment of cells in the sub-G_1_ phase using cell cycle analysis showed a time-dependent significant increase in the percentage of cell death after SFN exposure at 48 h in both melanoma cell lines, as up to 29 and 20% of A375 and 501MEL cells were dead, respectively (*p* = 0.0339 and *p* = 0.0023, respectively). In contrast, no significant effects were observed in the HEMa cells (Fig. 2b). To validate the pro-apoptotic effects of SFN, both the A375 and the 501MEL cells were analysed by flow cytometry using Annexin V–FITC. The percentage of total (early + late) apoptotic cells increased in a time-dependent manner. At 48 h post-treatment, the number of apoptotic cells reached 38 and 22% in the A375 and 501MEL cells, respectively (Fig. 2c). As previously shown, SFN exhibited a stronger apoptotic effect on the A375 cells than on the 501MEL cells. No effects were observed in the HEMa cells.

We further investigated the mechanisms underlying cell death induced by SFN by assessing proteins that play a crucial role in the cell cycle and apoptosis regulation. Immunoblotting on protein cell extracts revealed that SFN completely inhibited the expression of cyclin B1, a G_2_/M phase marker, in A375 cells at 48 h post-treatment, which agrees with the accumulation of cells in the G_2_/M phase. In the 501MEL cells, there was only a partial reduction (Fig. 2d, e). The levels of total p53 and phosphorylated p53 [p-p53 (Ser-15)] were strongly increased in a time-dependent manner in both melanoma cell lines. The levels of Bcl-2, an anti-apoptotic protein and a transcriptional target of p53, were also analysed. In accordance with p53 expression, Bcl-2 expression was decreased in both melanoma cell lines after SFN exposure (Fig. 2d, e). Moreover, to determine whether SFN-induced apoptosis in melanoma cells is caspase-dependent, caspase-8, caspase-9 and caspase-3 expression was assessed. Procaspase-9 was strongly decreased in a time-dependent manner in A375 cells after SFN treatment, suggesting a major role of the intrinsic apoptotic pathway. In contrast, no differences were detected in the 501MEL at any time point. Procaspase-8 was used to assess the extrinsic apoptotic pathway, and its levels were slightly reduced in 501MEL cells at 48 h post-treatment and slightly reduced in A375 cells at both 24 and 48 h post-treatment. Because of caspase-8 and caspase-9 activation, caspase-3 was completely cleaved in A375 cells at 48 h after SFN treatment, while caspase-3 cleavage started later and it was only partially cleaved after 48 h in 501MEL cells (Fig. 2d, e). Because caspase-3 is the most efficient processing enzyme for poly (ADP-ribose) polymerase (PARP), we also analysed its activation. SFN activated PARP cleavage in both melanoma cell lines. PARP was remarkably cleaved at 24 h in A375 cells and persisted until at least 48 h after treatment, while PARP cleavage was only detected in 501MEL cells at 48 h (Fig. 2d, e). These findings highlight the differences in the apoptotic mechanism of SFN between primary and metastatic human melanoma cells.

### SFN hampered A375 cell migration and invasion

**Fig. 3:**
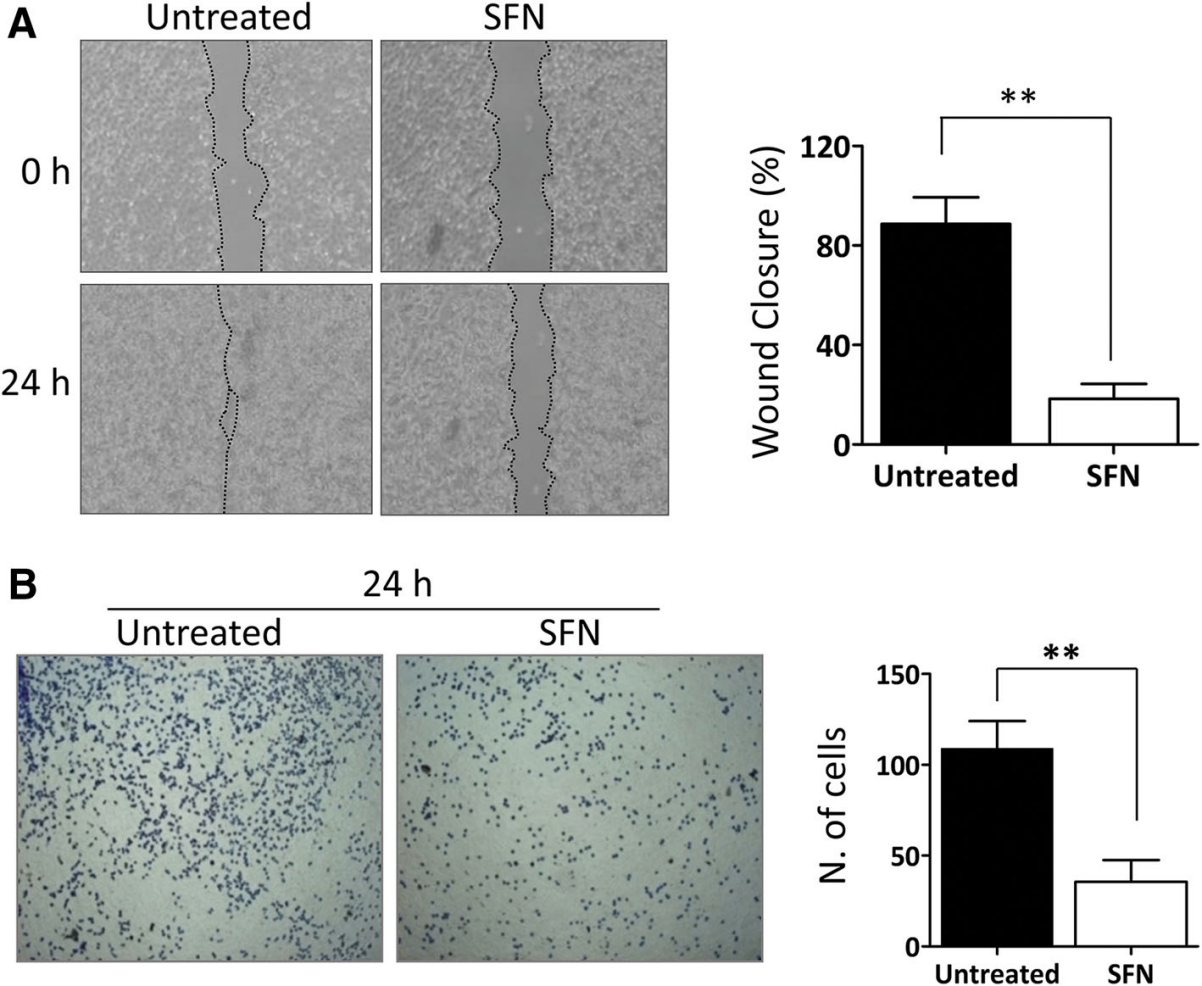
SFN reduces cell migration and invasion. **a** Representative images from the scratch assay of A375 cells captured at 0 and 24 h after treatment with 2 µg/ml SFN by a phase-contrast microscope (10×). The percentage of the closed wound area was calculated by tracing the border around the wound using ImageJ software. Data represent the mean ± SD from four separate experiments. **b** Representative images of A375 cells treated with 2 µg/ml SFN at 24 h after collagen invasion. Migrated cell nuclei were stained with methylene blue; images were captured by a phase-contrast microscope (4×) and the number of cells that migrated into the collagen was counted. Data represent the mean ± SD of six fields in triplicate. The statistical analysis was performed by paired two-tailed Student’s test (*p < 0.05, **p < 0.01 and ***p < 0.001)

To evaluate the effects of SFN on melanoma cell migration, we performed a scratch assay. The data demonstrated that SFN treatment strongly reduced wound closure in A375 cells at 24 h. Only 18% of the tumour cells migrated compared to 89% for the untreated cells (*p* = 0.0022; Fig. 3a). The difference in the migration ability between the treated and untreated cells cannot be ascribed to a difference in their proliferation rates because the cells were treated with mitomycin prior to SFN treatment. To measure the impact of SFN on tumour cell invasion, we counted the number of cells that could penetrate type I collagen matrix. The data showed a significant reduction in the number of tumour cells invading the collagen compared to that of untreated cells (108 vs. 35.5, *p* = 0.0057) (Fig. 3b). All together, these results clearly suggest that SFN affects the migration and invasion ability of melanoma cells.

### SFN altered the transcriptome profile of melanoma cells

**Fig. 4:**
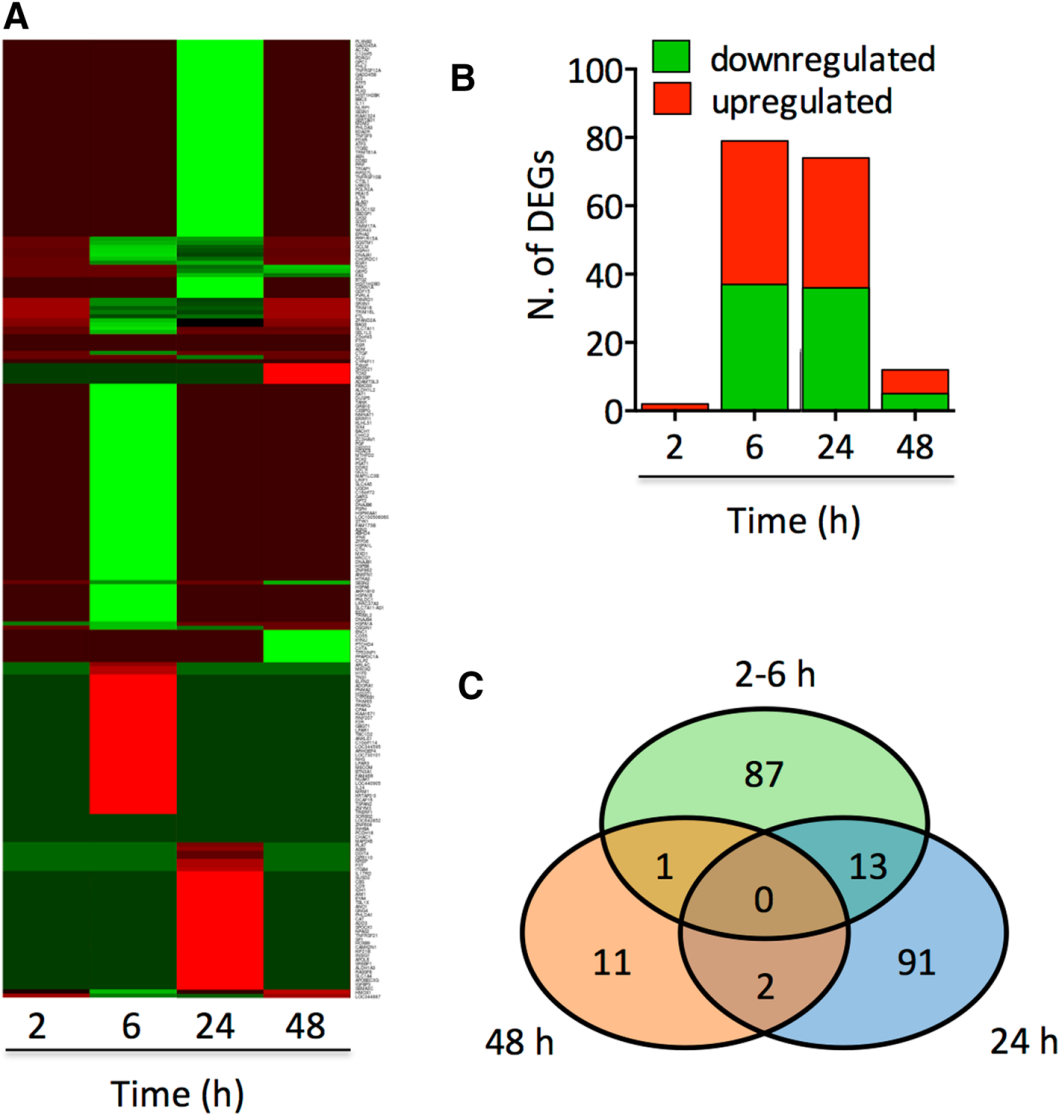
SFN induces changes in gene expression profiles. **a** Heat map summary reflecting gene expression values of A375 cells treated with 2 µg/ml SFN at different time points (*columns*). *Red* indicates high and *green* indicates low gene expression values. **b** The histograms represent the number of DEGs at different time points after SFN treatment. **c** Venn diagram showing the overlap between the differentially expressed genes in the A375 cells treated with 2 µg/ml SFN at different time points

To decipher the antitumour molecular mechanisms driven by SFN, we investigated the whole transcriptome of A375 cells at four different time points (2, 6, 24 and 48 h after SFN treatment) using Illumina RNA-Seq technology. The results from two independent experiments revealed a total of 329 differently expressed genes (DEGs). Among these genes, 219 were affected by SFN treatment (Table 1), while 110 genes were intrinsically modulated during cell growth in culture (without compound treatment) (Supplementary Table S1). Out of the 219 genes, 74 were downregulated (34%) and 145 were upregulated (66%) by SFN at least at one time point (Fig. 4a, b; Table 1). Only two genes were upregulated at 2 h post-treatment, heat shock 70 kDa protein 1A (*HSPA1A*) and heme oxygenase (decycling) 1 (*HMOX1*). The number of DEGs modulated by SFN was much higher at 6 and 24 h after exposure compared to that at 48 h post-treatment (Fig. 4a, b; Table 1). Most genes were specifically affected at a unique time point, and only a few genes shared differential expression over several hours post-SFN treatment, specifically between 6 and 24 h (Fig. 4c).

**Table 1:** Upregulated and downregulated genes in A375 cells treated with SFN at different time points

Time (h)	Gene name and symbol	Fold change
2	Heat shock 70 kDa protein 1A (HSPA1A)	3.18
	Heme oxygenase (decycling) 1 (HMOX1)	2.68
6	Heme oxygenase (decycling) 1 (HMOX1)	6.12
	Heat shock 70 kDa protein 6 (HSP70B’ (HSPA6)	4.40
	NmrA-like family domain containing 1 pseudogene (LOC344887)	4.39
	Aldo-keto reductase family 1, member B10 (aldose reductase) (AKR1B10)	4.39
	Oxidative stress-induced growth inhibitor 1 (OSGIN1)	4.38
	Heat shock 70 kDa protein 1B (HSPA1B)	4.19
	Heat shock 70 kDa protein 1A (HSPA1A)	4.12
	Poly(A)-specific ribonuclease (PARN)-like domain containing 1 (PNLDC1)	4.01
	Tripartite motif family-like 2 (TRIML2)	3.39
	Solute carrier family 7 (anionic amino acid transporter light chain, xc-system), member 11 (SLC7A11)	3.36
	DnaJ (Hsp40) homolog, subfamily B, member 4 (DNAJB4)	3.33
	EP300 interacting inhibitor of differentiation 3 (EID3)	3.19
	Leucine rich repeat containing 37, member A3 (LRRC37A3)	3.08
	SLC7A11 antisense RNA 1 (SLC7A11-AS1)	3.02
	BCL2-associated athanogene 3 (BAG3)	2.75
	Tripartite motif containing 16-like (TRIM16L)	2.73
	Zinc finger, AN1-type domain 2A (ZFAND2A)	2.64
	Thioredoxin reductase 1 (TXNRD1)	2.54
	HtrA serine peptidase 3 (HTRA3)	2.52
	Sulfiredoxin 1 (SRXN1)	2.33
	Sel-1 suppressor of lin-12-like 3 (*C. elegans*) (SEL1L3)	2.28
	Zinc finger protein 862 (ZNF862)	2.25
	DnaJ (Hsp40) homolog, subfamily B, member 1 (DNAJB1)	2.11
	Tripartite motif containing 16 (TRIM16)	2.08
	MAX dimerization protein 1 (MXD1)	2.08
	Lysine-rich coiled-coil 1 (KRCC1)	2.07
	Heat shock 22 kDa protein 8 (HSPB8)	1.99
	Heat shock 70 kDa protein 1-like (HSPA1L)	1.91
	Glutamate–cysteine ligase, modifier subunit (GCLM)	1.89
	Heat shock 105 kDa/110 kDa protein 1 (HSPH1)	1.86
	Cystathionase (cystathionine gamma-lyase) (CTH)	1.86
	SMG1 homolog, phosphatidylinositol 3-kinase-related kinase (*C. elegans*) pseudogene (LOC100506060)	1.80
	Serine/threonine/tyrosine kinase 1 (STYK1)	1.79
	Family with sequence similarity 173, member B (FAM173B)	1.77
	Asparagine synthetase (glutamine-hydrolyzing) (ASNS)	1.75
	Protein phosphatase 1, regulatory subunit 15A (PPP1R15A)	1.72
	ZFP36 ring finger protein (ZFP36)	1.72
	Early growth response 1 (EGR1)	1.68
	ZFP36 ring finger protein (ZFP36)	1.72
	Early growth response 1 (EGR1)	1.68
	Abhydrolase domain containing 4 (ABHD4)	1.68
	Interferon, epsilon (IFNE)	1.68
	Ferritin, light polypeptide (FTL)	1.62
	Dual specificity phosphatase 5 (DUSP5)	1.62
	F-box protein 30 (FBXO30)	1.62
	Aldehyde dehydrogenase 1 family, member L2 (ALDH1L2)	1.62
	Spermidine/spermine N1-acetyltransferase 1 (SAT1)	1.62
	TRAF family member-associated NFKB activator (TANK)	1.61
	Growth factor receptor-bound protein 10 (GRB10)	1.58
	Sequestosome 1 (SQSTM1)	1.56
	CCAAT/enhancer binding protein (C/EBP), gamma (CEBPG)	1.54
	DnaJ (Hsp40) homolog, subfamily A, member 1 (DNAJA1)	1.54
	Nicotinamide nucleotide adenylyltransferase 1 (NMNAT1)	1.54
	ERBB receptor feedback inhibitor 1 (ERRFI1)	1.51
	Histone deacetylase 9 (HDAC9)	1.48
	Sestrin 2 (SESN2)	1.44
	Placental growth factor (PGF)	1.44
	Death effector domain containing 2 (DEDD2)	1.42
	Kelch-like family member 31 (KLHL31)	1.40
	SIX homeobox 4 (SIX4)	1.40
	BTB and CNC homology 1, basic leucine zipper transcription factor 1 (BACH1)	1.40
	Cysteine-rich hydrophobic domain 2 (CHIC2)	1.39
	Zinc finger CCCH-type, antiviral 1 (ZC3HAV1)	1.37
	Cysteine and histidine-rich domain (CHORD) containing 1 (CHORDC1)	1.30
	UDP-glucose 6-dehydrogenase (UGDH)	1.29
	Ligand-dependent nuclear receptor interacting factor 1 (LRIF1)	1.27
	Solute carrier family 4 (sodium bicarbonate cotransporter), member 5 (SLC4A5)	1.27
	Chromosome 16 open reading frame 72 (C16orf72)	1.25
	Glycyl-tRNA synthetase (GARS)	1.25
	Glutamate–cysteine ligase, catalytic subunit (GCLC)	1.22
	NAD-dependent methylene tetrahydrofolate dehydrogenase cyclohydrolase (MTHFD2)	1.21
	Phosphoenolpyruvate carboxykinase 2 (mitochondrial) (PCK2)	1.21
	Phosphoserine aminotransferase 1 (PSAT1)	1.21
	Discoidin domain receptor tyrosine kinase 2 (DDR2)	1.20
	Microtubule-associated protein 1 light chain 3 beta (MAP1LC3B)	1.18
	Glutamic pyruvate transaminase (alanine aminotransferase) 2 (GPT2)	1.15
	DnaJ (Hsp40) homolog, subfamily B, member 6 (DNAJB6)	1.15
	Connective tissue growth factor (CTGF)	1.14
	Phosphoserine phosphatase (PSPH)	1.14
	Heat shock protein 90 kDa alpha (cytosolic), class A member 1 (HSP90AA1)	1.09
	Peroxisome proliferator-activated receptor gamma (PPARG)	−1.16
	Carboxypeptidase A4 (CPA4)	−1.19
	Tripartite motif containing 65 (TRIM65)	−1.23
	Uncharacterized protein KIAA1671 (KIAA1671)	−1.30
	Ring finger protein 207 (RNF207)	−1.33
	Coagulation factor II (thrombin) receptor (F2R)	−1.34
	Lysophosphatidic acid receptor 1 (LPAR1)	−1.37
	TBC1 domain family, member 2 (TBC1D2)	−1.38
	Ankyrin repeat and LEM domain containing 1 (ANKLE1)	−1.38
	Globoside alpha-1,3-*N*-acetylgalactosaminyltransferase 1 (GBGT1)	−1.42
	Mesenchyme homeobox 2 (MEOX2)	−1.52
	Interleukin 24 (IL24)	−1.57
	Mitochondrial rRNA methyltransferase 1 homolog (S. cerevisiae) (MRM1)	−1.61
	Keratin-associated protein 2-3 (KRTAP2-3)	−1.63
	DDB1 and CUL4-associated factor 16 (DCAF16)	−1.64
	Tetraspanin 2 (TSPAN2)	−1.66
	Zinc finger, MYM-type 3 (ZMYM3)	−1.66
	Transcriptional regulating factor 1 (TRERF1)	−1.67
	NUAK family, SNF1-like kinase, 1 (NUAK1)	−1.70
	Family with sequence similarity 46, member B (FAM46B)	−1.72
	MDS1 and EVI1 complex locus (MECOM)	−1.75
	H1 histone family, member 0 (H1F0)	−1.78
	Butyrophilin, subfamily 3, member A1 (BTN3A1)	−1.78
	ADP-ribosylation factor-like 4C (ARL4C)	−1.79
	Rho guanine nucleotide exchange factor (GEF) 4 (ARHGEF4)	−1.85
	Uncharacterized LOC730101 (LOC730101)	−1.91
	Nance–Horan syndrome (congenital cataracts and dental anomalies) (NHS)	−1.93
	Lysophosphatidic acid receptor 3 (LPAR3)	−1.93
	Cancer susceptibility candidate 10 (C10orf114)	−2.04
	Paraneoplastic Ma antigen 2 (PNMA2)	−2.34
	Heparan sulfate (glucosamine) 3-*O*-sulfotransferase 1 (HS3ST1)	−2.38
	Cytochrome P450, family 26, subfamily B, polypeptide 1 (CYP26B1)	−2.39
	Extracellular leucine-rich repeat and fibronectin type III domain containing 2 (ELFN2)	−2.45
	Adenosine A1 receptor (ADORA1)	−2.47
	Tensin 1 (TNS1)	−3.00
24	Poliovirus receptor-related 4 (PVRL4)	4.64
	NmrA-like family domain containing 1 pseudogene (LOC344887)	3.99
	Oxidative stress-induced growth inhibitor 1 (OSGIN1)	3.20
	Growth differentiation factor 15 (GDF15)	3.15
	BTG family, member 2 (BTG2)	2.83
	Histone cluster 1, H2bd (HIST1H2BD)	2.82
	Cyclin-dependent kinase inhibitor 1A (p21, Cip1) (CDKN1A)	2.67
	Heme oxygenase (decycling) 1 (HMOX1)	2.29
	Fas cell surface death receptor (FAS)	2.28
	Ferredoxin reductase (FDXR)	2.28
	Activating transcription factor 3 (ATF3)	2.23
	Tripartite motif containing 16-like (TRIM16L)	2.22
	Integrin, beta 2 (complement component 3 receptor 3 and 4 subunit) (ITGB2)	2.14
	KIAA1324 (KIAA1324)	2.07
	SERTA domain containing 1 (SERTAD1)	2.06
	Tripartite motif containing 16 (TRIM16)	2.02
	MDM2 oncogene, E3 ubiquitin protein ligase (MDM2)	2.01
	Pleckstrin homology-like domain, family A, member 3 PHLDA3	1.99
	Early growth response 1 (EGR1)	1.92
	Ectodysplasin A2 receptor (EDA2R)	1.91
	Tumor necrosis factor (ligand) superfamily, member 9 (TNFSF9)	1.91
	Thioredoxin reductase 1 (TXNRD1)	1.91
	Ferritin, light polypeptide (FTL)	1.87
	Sulfiredoxin 1 (SRXN1)	1.84
	BCL2 binding component 3 (BBC3)	1.83
	Interleukin 11 (IL11)	1.75
	NLR family, pyrin domain containing 1 (NLRP1)	1.63
	Sestrin 1 (SESN1)	1.62
	Phosphatase 1, regulatory subunit 15A (PPP1R15A)	1.61
	Polo-like kinase 3 (PLK3)	1.51
	Histone cluster 1, H2bk (HIST1H2BK)	1.48
	BCL2-associated X protein (BAX)	1.41
	Inhibitor of DNA binding 3, dominant negative helix-loop-helix protein (ID3)	1.38
	Activating transcription factor 5 (ATF5)	1.36
	Sequestosome 1 (SQSTM1)	1.34
	Plexin B2 (PLXNB2)	1.31
	Growth arrest and DNA damage-inducible, alpha (GADD45A)	1.31
	Actin, alpha 2, smooth muscle, aorta (ACTA2)	1.30
	Chromosome 12 open reading frame 5 (C12orf5)	1.27
	p53 and DNA damage regulated 1 (PDRG1)	1.25
	Glucose-6-phosphate dehydrogenase (G6PD)	1.24
	Glypican 1 (GPC1)	1.22
	Four-and-a-half LIM domains 2 (FHL2)	1.22
	Clusterin (CLU)	1.21
	Tumor necrosis factor receptor superfamily, member 12A (TNFRSF12A)	1.20
	Growth arrest and DNA damage-inducible, beta (GADD45B)	1.19
	Glutamate–cysteine ligase, modifier subunit (GCLM)	1.19
	TP53 regulated inhibitor of apoptosis 1 (TRIAP1)	1.15
	Ribosomal protein S27-like (RPS27L)	1.13
	Peptidylprolyl isomerase F (PPIF)	1.11
	Zinc finger, AN1-type domain 2A (ZFAND2A)	1.09
	Damage-specific DNA binding protein 2, 48 kDa (DDB2)	1.09
	tRNA methyltransferase 61 homolog A (*S. cerevisiae*) (TRMT61A)	1.09
	Apoptosis enhancing nuclease (AEN)	1.09
	Heat shock 105 kDa/110 kDa protein 1 (HSPH1)	1.08
	Polymerase (RNA) II (DNA directed) polypeptide A, 220 kDa (POLR2A)	1.05
	Ubiquitin-conjugating enzyme E2S (UBE2S)	1.03
	Tumor necrosis factor receptor superfamily, member 10b (TNFRSF10B)	1.02
	Cathepsin L (CTSL1)	1.02
	Phosphoprotein enriched in astrocytes 15 (PEA15)	0.98
	BCL2-associated athanogene 3 (BAG3)	0.97
	Partner of NOB1 homolog (S. cerevisiae) (PNO1)	0.94
	Interleukin 7 receptor (IL7R)	0.92
	Cysteine and histidine-rich domain (CHORD) containing 1 (CHORDC1)	0.91
	Aminolevulinate, delta-, synthase 1 (ALAS1)	0.91
	Biogenesis of lysosomal organelles complex-1, subunit 2 (BLOC1S2)	0.86
	Shwachman–Bodian–Diamond syndrome pseudogene 1 (SBDSP1)	0.84
	DnaJ (Hsp40) homolog, subfamily A, member 1 (DNAJA1)	0.80
	CDC28 protein kinase regulatory subunit 2 (CKS2)	0.77
	Superoxide dismutase 1, soluble (SOD1)	0.77
	Translocase of inner mitochondrial membrane 17 homolog A (yeast) (TIMM17A)	0.75
	WD repeat domain 43 (WDR43)	0.75
	EPH receptor A2 (EPHA2)	0.72
	Catalase (CAT)	−0.87
	Adducin 3 (gamma) (ADD3)	−0.88
	Sparc/osteonectin, cwcv and kazal-like domains proteoglycan (testican) 1 (SPOCK1)	−0.89
	Tumor necrosis factor receptor superfamily, member 21 (TNFRSF21)	−0.91
	Sp1 transcription factor (SP1)	−0.91
	Neuronal PAS domain protein 2 (NPAS2)	−0.92
	Guanine nucleotide binding protein (G protein), gamma 4 (GNG4)	−0.95
	Pleckstrin homology-like domain, family A, member 1 PHLDA1	−0.97
	Plasminogen activator, tissue (PLAT)	−1.02
	CD9 molecule (CD9)	−1.02
	Isocitrate dehydrogenase 1 (NADP+), soluble (IDH1)	−1.04
	Eyes absent homolog 4 (Drosophila) (EYA4)	−1.06
	Transducin (beta)-like 1X-linked (TBL1X)	−1.07
	Anoctamin 1, calcium activated chloride channel (ANO19)	−1.07
	Absent in melanoma 1 (AIM1)	−1.09
	Aldehyde dehydrogenase 1 family, member A3 (ALDH1A3)	−1.27
	Ras association (RalGDS/AF-6) domain family (N-terminal) member 8 (RASSF8)	−1.28
	Sterol regulatory element binding transcription factor 1 (SREBF1)	−1.31
	DNA damage-inducible transcript 4 (DDIT4)	−1.32
	Ankyrin repeat and SOCS box containing 9 (ASB9)	−1.37
	Solute carrier family 1 (SLC1A4)	−1.38
	Apolipoprotein B mRNA editing enzyme, catalytic polypeptide-like 3G (APOBEC3G)	−1.39
	Insulin-like growth factor binding protein 3 (IGFBP3)	−1.45
	Domain, immunoglobulin domain (Ig), short basic domain, secreted, (semaphorin) 3C (SEMA3C)	−1.46
	Calcium/calmodulin-dependent protein kinase II inhibitor 1 (CAMK2N1)	−1.55
	Kinesin family member 21B (KIF21B)	−1.56
	Insulin-induced gene 1 (INSIG1)	−1.56
	Apolipoprotein L, 6 (APOL6)	−1.57
	Protein-coupled receptor 110 (GPR110)	−1.59
	Homeobox B9 (HOXB9)	−1.70
	Neuronal regeneration related protein (NREP)	−1.78
	Sushi domain containing 2 (SUSD2)	−1.90
	Cystathionine-beta-synthase (CBS)	−2.04
	Follistatin (FST)	−2.08
	Interleukin 17 receptor D (IL17RD)	−2.20
	Integrin, beta 4 (ITGB4)	−2.32
48	Phosphatidic acid phosphatase type 2 domain containing 1A (PPAPDC1A)	3.40
	Cartilage intermediate layer protein 2 (CILP2)	3.32
	Tumor protein p53 inducible nuclear protein 1 (TP53INP1)	2.39
	Patched domain containing 4 (PTCHD4)	2.11
	Class II, major histocompatibility complex, transactivator (CIITA)	2.02
	Kynureninase (KYNU)	1.83
	Ectodermal-neural cortex 1 (with BTB domain) (ENC1)	1.82
	CD55 molecule, decay accelerating factor for complement (Cromer blood group) (CD55)	1.81
	Fas cell surface death receptor (FAS)	1.71
	Sestrin 2 (SESN2)	1.61
	Glucose-6-phosphate dehydrogenase (G6PD)	1.54
	TOX high mobility group box family member 2 (TOX2)	−1.78
	ABI family, member 3 (NESH) binding protein (ABI3BP)	−2.63
	ADAMTS-like 3 (ADAMTSL3)	−3.44

Enrichment cluster analysis of gene functions and biological processes was carried out using the WEB-based GEne SeT AnaLysis Toolkit (WebGestalt). Gene ontology analysis based on biological processes, molecular functions and cellular components are presented in Table 2, and the list of genes in each category is presented in Supplement Table S2. Clustering based on the biological process classification revealed that SFN-regulated genes are predominantly involved in response to stress (86 genes, *p* = 2.43e−12), the apoptotic process/cell death (60 genes, *p* = 3.44e−11), response to topologically incorrect proteins (16 genes, *p* = 9.47e−09), response to different stimuli (129 genes, *p* = 9.47e−08) and positive regulation of metabolic processes (50 genes, *p* = 0.0001). Molecular function clustering revealed that SFN regulates genes generally involved in protein binding (135 genes, *p* = 6.97e−08). According to the cellular component classification, SFN induces the expression of genes involved in the intracellular ferritin pathway (2 genes, *p* = 0.012) and the glutamate–cysteine ligase complex (2 genes, *p* = 0.012).

**Table 2:** Gene ontologies cluster analysis show global effects of SFN on human melanoma cells (WebGestalt analysis)

GO category	GO_ID	GO_ID number of genes in the category	*p* value
*Biological process*			
Response to stress	GO:0006950	86	2.43e−12
Apoptotic process	GO:0006915	57	3.44e−11
Programmed cell death	GO:0012501	57	3.44e−11
Death	GO:0016265	60	3.44e−11
Cell death	GO:0008219	60	3.44e−11
Response to topologically incorrect protein	GO:0035966	16	9.47e−09
Response to unfolded protein	GO:0006986	15	3.87e−08
Regulation of programmed cell death	GO:0043067	43	4.39e−08
Regulation of cell death	GO:0010941	43	8.30e−08
Regulation of apoptotic process	GO:0042981	42	9.08e−08
Response to stimulus	GO:0050896	129	9.47e−08
Cellular response to stress	GO:0033554	42	2.99e−07
Response to organic substance	GO:0010033	54	4.43e−07
Response to chemical stimulus	GO:0042221	69	1.38e−06
Positive regulation of biological process	GO:0048518	75	2.99e−06
Negative regulation of cellular process	GO:0048523	68	7.14e−06
Positive regulation of cellular process	GO:0048522	72	8.82e−06
Negative regulation of biological process	GO:0048519	71	1.94e−05
Execution phase of apoptosis	GO:0097194	15	2.86e−05
Regulation of execution phase of apoptosis	GO:1900117	13	2.99e−05
Apoptotic signalling pathway	GO:0097190	13	2.99e−05
Cellular response to stimulus	GO:0051716	98	3.49e−05
Response to abiotic stimulus	GO:0009628	29	4.88e−05
Intrinsic apoptotic signalling pathway	GO:0097193	10	9.05e−05
Positive regulation of metabolic process	GO:0009893	50	0.0001
Neuron death	GO:0070997	12	0.0001
Regulation of response to stimulus	GO:0048583	55	0.0001
Glutathione metabolic process	GO:0006749	7	0.0001
Serine family amino acid metabolic process	GO:0009069	6	0.0001
Cysteine metabolic process	GO:0006534	4	0.0001
Regulation of protein metabolic process	GO:0051246	39	0.0002
Neuron apoptotic process	GO:0051402	11	0.0002
Homeostasis of number of cells	GO:0048872	12	0.0002
Signal transduction	GO:0007165	85	0.0002
Regulation of metabolic process	GO:0019222	90	0.0003
Positive regulation of cell death	GO:0010942	19	0.0003
Response to oxygen levels	GO:0070482	14	0.0004
Regulation of signal transduction	GO:0009966	44	0.0005
Positive regulation of cellular metabolic process	GO:0031325	46	0.0006
*Molecular function*			
Protein binding	GO:0005515	135	6.97e−08
Protein dimerization activity	GO:0046983	31	0.0001
Cofactor binding	GO:0048037	15	0.0001
Binding	GO:0005488	170	0.0005
Identical protein binding	GO:0042802	27	0.0007
Vitamin B6 binding	GO:0070279	6	0.0028
Pyridoxal phosphate binding	GO:0030170	6	0.0028
Glutamate–cysteine ligase activity	GO:0004357	2	0.0065
*Cellular component*			
Ferritin complex	GO:0070288	2	0.0116
Intracellular ferritin complex	GO:0008043	2	0.0116
Glutamate–cysteine ligase complex	GO:0017109	2	0.0116
Cytosol	GO:0005829	46	0.0217
Intracellular organelle lumen	GO:0070013	57	0.0373
Organelle lumen	GO:0043233	58	0.0373
Membrane-enclosed lumen	GO:0031974	58	0.0373
Nucleus	GO:0005634	88	0.0544
Nuclear part	GO:0044428	52	0.0696
Nuclear lumen	GO:0031981	47	0.0818

### Target verification by qPCR

**Fig. 5:**
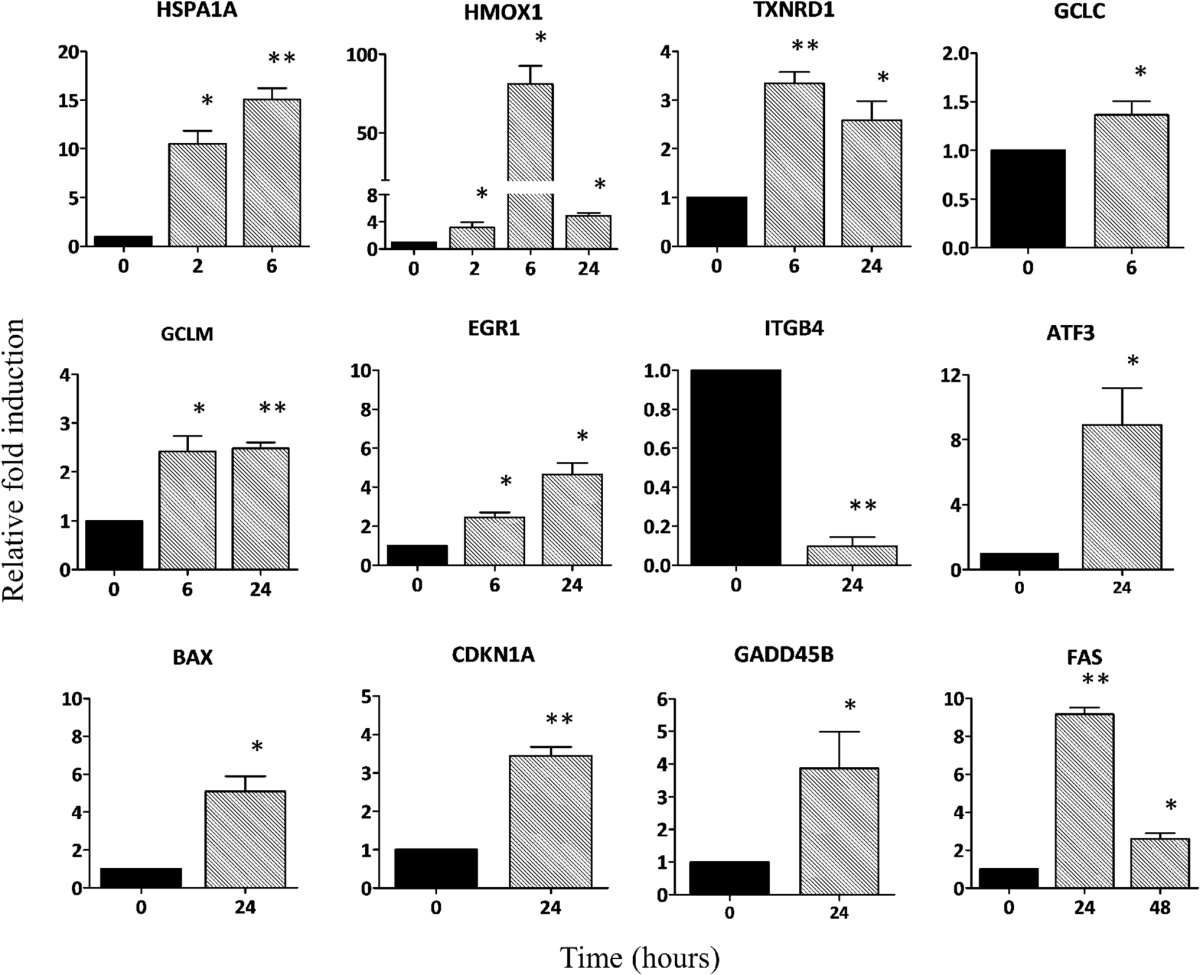
Quantitative PCR validation of 12 DEGs in A375 cells treated with SFN. Relative fold expression of candidate genes was normalized against GADPH expression. The data represent the mean  ±  SD of three independent replicates. The *black bars* represent untreated cells at time 0, and the *grey bars* represent the relative fold change after SFN treatment. Statistical analysis was performed by paired two-tailed Student’s test (*p < 0.05, **p < 0.01)

To verify the expression patterns of the DEGs detected by RNA-Seq, 12 genes that are modulated by SFN were selected for quantitative real-time PCR analysis, and *GADPH* was used as the reference gene. This includes five stress response genes [(*HSPA1A*, *HMOX1*, thioredoxin reductase 1 (*TXNRD1*), glutamate–cysteine ligase, catalytic subunit (*GCLC*) and modifier subunit (*GCLM*))], six p53 network genes [early growth response 1 (*EGR1*), activating transcription factor 3 (*ATF3*), BCL2-associated X protein (*BAX*), Fas cell surface death receptor (*FAS*), growth arrest and DNA damage-inducible beta (*GADD45B*) and cyclin-dependent kinase inhibitor 1A (*CDKN1A*))] and integrin beta 4 (*ITGB4*). The qPCR results showed high concordance with the RNA-Seq data, although the fold change values varied accordingly with the analytical method, suggesting that the RNA-Seq findings are reliable (Fig. 5). However, these data support the observation that SFN regulates the transcription of genes related to the Nrf2-signaling pathway and its involvement in physiological processes in melanoma cells.

### Protein interaction network of differentially expressed proteins (DEPs)

**Fig. 6:**
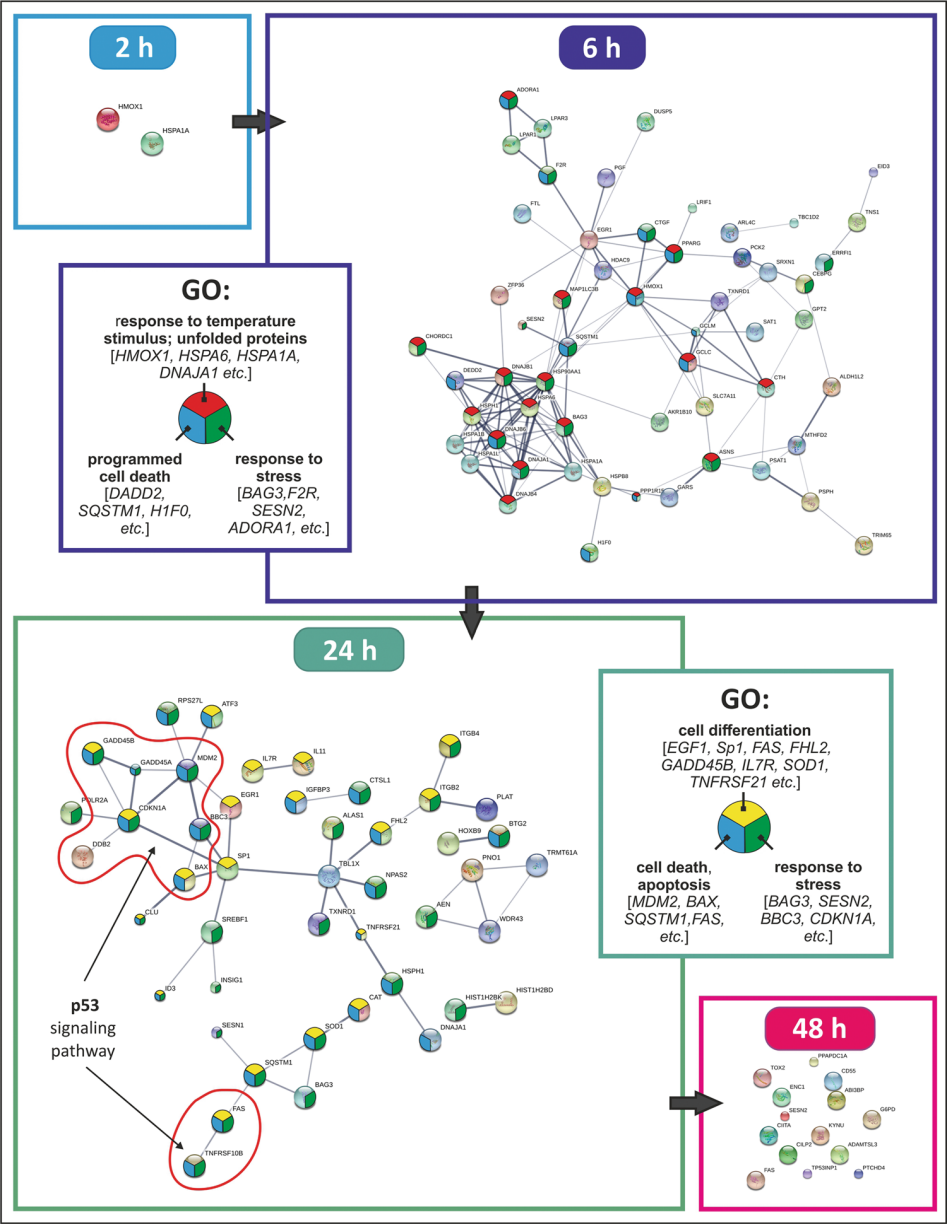
Protein interaction network analysis using STRING. Representation of a functional protein association network of DEGs from A375 cells treated with 2 µg/ml SFN at 2, 6, 24 and 48 h post-treatment. The* coloured* nodes represent query proteins and first shell interactions. The* line thickness* indicates the strength of data support

The functional partnerships and interactions that occur between proteins corresponding to the revealed DEGs were predicted by STRING network analysis [25]. The downregulated and upregulated genes were linked into the networks according to their physical and functional associations and their involvement in specific cellular pathways (Fig. 6; Supplement Table S3). Early response to the drug (2 h post-treatment) was clearly associated with overexpression of the *HSPA1A* and *HMOX1* genes. We detected upregulation of the *HMOX1* gene across all the time points except the last one (48 h), with the maximum expression being reached at 6 h after exposure. At 6 h after SFN exposure, the number of stimulated genes increased greatly (Fig. 4; Table 1). Out of 114 DEGs, 30 upregulated genes could be organized into a network according to their function: (a) response to stress; (b) protein unfolding response and response to temperature; (c) regulation of cell death. Only five interacting proteins were downregulated, all of which were receptors [coagulation factor II (thrombin) receptor (*F2R*), lysophosphatidic acid receptor 1 and 3 (*LPAR1/3*), adenosine A1 receptor (*ADORA1*) and peroxisome proliferator-activated receptor gamma (*PPARG*)]. Prolonged SFN treatment (24 h) upregulated the expression of 73 genes and downregulated the expression of 36 genes (Fig. 4; Table 1). The upregulated genes mainly included genes related to apoptosis, including p53-related genes (*MDM2*, *BAX*, *GADD45A*, *CDKN1A*, *ATF3*, *FAS*), and genes involved in the growth arrest and proliferation (*EGR1*, *BTG2*). The stress response network was still activated, including the *TXNRD1* and *SOD1* genes and the histone protein cluster genes *HIST1H2BD* and *HIST1H2BK*. The downregulated genes include plasminogen activator tissue (*PLAT*), *ITGB2* and *ITGB4* grouped into the network, which could be involved in the regulation of cell migration and tissue remodelling (Fig. 6). Transcription factor-specific protein 1 (*SP1*) has been shown to be upregulated in several type of cancers; it is associated with poor prognosis and could be implicated in prostate cancer chemoprevention [26]. Our analysis demonstrated that SFN-treated A375 cells showed reduced *SP1* expression at 24 h, which orchestrated the coordinated overexpression of 16 other proteins in the network (Fig. 6). The transcriptional co-factor transducin beta-like (*TBL1X*) 1 has been demonstrated to be involved in cellular proliferation and invasiveness in both human and murine pancreatic ductal adenocarcinoma [27]. Our data also indicated a crucial role for this protein, as it is downregulated at 24 h by SFN and probably controls the expression of ten other genes in the network. At 48 h, SFN upregulated a small number of genes related to the final stage of stress response and p53-related genes that play a role in apoptosis, such as TP53-inducible nuclear protein 1 (*TP53INP1*), sestrin 2 (*SESN2*), Fas cell surface death receptor (*FAS*) and glucose-6-phosphate dehydrogenase (*G6PD*) (Fig. 6; Table 1).

## Discussion

Over the last few years, an increase in research using dietary phytochemicals for targeting cancer has been driven by the partial efficacy of chemotherapy and radiotherapy and the associated side effects. Isothiocyanates and in particular SFN have been investigated in vitro and in vivo studies. SFN has long been known to act as a therapeutic or preventive agent and an inducer of apoptosis in pre-cancerous cells and tumour cells of different origins [18, 28–33]. Previous studies have indicated an anticancer activity of SFN in melanoma [24, 34]. SFN has been shown to inhibit the metastasis of murine B16F-10 melanoma cells by inducing apoptosis via caspase activation both in vitro and in vivo. Moreover, partial analysis on the effects of SFN on A375 cell line has been reported indicating a reduction on cell viability, but only at 10 μM onward or after a treatment had been refreshed after 24 h (at 5 μM). No activation of apoptosis mediated by caspase 4, 8, 9 was revealed or other cellular mechanisms such as proliferation, cell cycle progression, invasion and migration analysed. Few reports have shown the pro-apoptotic effects of SFN on human melanoma cells by the p53 and p38 pathways and the anti-proliferative effects associated with loss of the chromatin modifying enzyme EZH2 [35, 36]. Nonetheless, the detailed molecular mechanisms by which SFN affects human melanoma are still unknown. Therefore, we implemented a comprehensive investigation at the cellular and molecular level to evaluate the effects of SFN, for the first time, on both primary and metastatic human melanoma cells and compared with its effects on normal epidermal melanocytes. This study represents an attempt to focus on specific processes and genes involved in response to SFN treatment in melanoma using functional assays and a transcriptomic approach. The results demonstrated that while SFN impairs the viability of both A375 and 501MEL cells in a dose- and time-dependent manner, it does not affect non-cancerous HEMa cells. To identify the main mechanisms responsible for growth inhibition, AKT phosphorylation and apoptosis were detected in all the cell lines tested. In agreement with previous reports [36, 37], our data showed that inhibition of cell viability is correlated with reduced AKT phosphorylation. Many studies have focused on the SFN-driven induction of apoptosis in different cancer cell types [19, 37–39]. Consistent with some previous reports [36], we demonstrated that SFN increases the percentage of apoptotic melanoma cells by cleaving PARP, activating p-p53, caspase-3, caspase-8 and caspase-9 and decreasing Bcl2 expression, confirming its pro-apoptotic role. Moreover, our data suggested that metastatic 501MEL cells are more resistant to SFN treatment than primary A375 melanoma cells.

Several studies have demonstrated that SFN causes G_1_/S and G_2_/M cell cycle arrest by altering the levels of cyclin A, cyclin B1, cyclin D1 and p21cip1/waf1 in cancer [40, 41]. Indeed, our data indicated that cell cycle arrest occurred in the melanoma cell lines treated with SFN, while no changes were observed in the HEMa cells. In fact, the results showed a strong accumulation of cells in the G_2_/M phase in a time-dependent manner, followed by a decrease in the number of cells in the G_0_/G_1_ phase. G_2_/M cell cycle arrest is reportedly associated with checkpoint damage, which is regulated by cyclin B1/CDK1 and the cyclin kinase inhibitor p21 in several cell lines [42–44]. We demonstrated that SFN induced G_2_/M arrest via inhibiting cyclin B1, but it did not affect cyclin A (data not shown). SFN strongly reduced the migration and invasion of melanoma A375 cells, which is in agreement with previous studies performed on various cancer cell lines [23, 38, 45–47], suggesting the importance of this compound in tumour spreading.

For the first time, we also examined the gene expression profile of A375 cells treated with SFN at different time points (2, 6, 24 and 48 h) using RNA-seq technology. Most of the genes (145) were upregulated, and only 74 genes were downregulated. In the early stages after treatment (from 2 to 6 h), we detected notable coordination of the induction of genes encoding the stress-inducible antioxidant protein Heme oxygenase-1 (*HMOX1*) and the heat shock-associated genes *HSPA1A*, *HSPA6*, *HSPA1B*, *HSPA1L*, *HSPB8*, *HSPH1* and *HSP90AA1*. This was followed by activation of the p53 signalling pathway and p53-dependent apoptotic processes. Heat shock proteins and HMOX1 play an important cytoprotective role during and after exposure to stress, possess powerful anti-apoptotic properties and promote cell survival under various pathological conditions. Thus, the role of these proteins in cancer seems to be ambiguous. Our data showed peak expression of stress-inducible proteins promptly after SFN treatment (from 2 to 6 h), which gradually faded after prolonged SFN treatment (24–48 h) as the apoptotic process began. Consistent with previous reports [48, 49] that associated the Nrf2 pathway with SFN-induced chemoprevention, we found that the Nrf2 target genes *HMOX1*, *TXNRD1*, *GCLC*, *GCLM*, *AKR1B10* and *G6PD* were upregulated after melanoma treatment.

As expected, a number of cell proliferation gene inhibitors were activated, including *GADD45* (encoding for growth arrest) and DNA damage-inducible proteins, which are involved in the G_2_/M checkpoint and may participate in the regulation of Cdc2 kinase activity and the activation of the p38/Jun N-terminal kinase pathway [43, 50]. Both *GADD45A* and *GADD45B* were upregulated in A375 cells treated with SFN for 24 h. Additionally, transcription factor 3 (*ATF3*), another cell growth repressor and pro-apoptotic gene [51, 52], was significantly upregulated after treatment. In line with the above observation, the anti-apoptotic and pro-survival adenosine receptor ADORA1 [53] was found to be downregulated by SFN. The nuclear transcription factor *EGR1* plays a role in signal transduction pathways mediating cellular proliferation and growth arrest and in the control of cell differentiation and death genes through the upregulation of p53 [54, 55] and PTEN [56]. Our data demonstrated that SFN increases the expression of *EGR1* in melanoma cells, which is in agreement with recent breast cancer data [57].

Finally, we clearly demonstrated that highly expressed p53 is strongly correlated with SFN-induced apoptosis in melanoma. Further, the pro-apoptotic genes *BAX*, *BBC3* [p53 upregulated modulator of apoptosis (PUMA)] and *FAS* were also found to be upregulated at the transcriptional level. These three genes are important players in the apoptotic process, because they are directly transactivated by p53 [58–61]. The upregulation of *BAX* and *PUMA* after SFN treatment has been previously demonstrated in different tumours [36, 62–64]. In addition, our results indicated that SFN enhances *FAS/CD95* gene expression at 24 and 48 h, suggesting that the FAS–FASL signalling pathway also contributes to SFN-induced apoptosis in melanoma cells. Similarly, SFN-induced apoptosis by FAS/CD95 has been reported for human leukaemia [65] and human prostate cancer cells [66].

Migration and invasion are crucial events that occur in the metastasis of primary tumours, including melanoma, and understanding all aspects of this process is essential to prevent cancer-related death. Several genes involved in invasion and migration were affected by SFN, including *HTRA3*, *PLAT*, *INHBA*, *FST* and *ITGB4*. In, particular, the well-characterized invasion inhibitor [67] serine protease *HTRA3* was shown to be upregulated. In contrast, tissue-type plasminogen activator (*PLAT*) was found to be downregulated at 24 h; this gene encodes a secreted serine protease that converts the pro-enzyme plasminogen to plasmin, which is a fibrinolytic enzyme that plays a role in cell migration and tissue remodelling [68]. The follistatin (*FST*) gene, related to lymphangiogenesis and cell growth, was downregulated at 24 h. Activin A plays a role in melanoma migration and reduces lymphatic endothelial sprout formation in vitro, but because of its pleiotropic effects on cell mobility, it is not suitable as a pharmacological target [69]. In addition, integrin β4 (*ITGB4*) downregulation was also observed. *ITGB4* promotes invasion and migration in various cancer cells [70]. As a receptor for the laminins, ITGB4 is essential for the organization and maintenance of epithelial structure and plays a pivotal role in the metastasis of various cancer cells [71].

SFN has demonstrated chemopreventive properties with selective cytotoxicity in several type of cancer based on its ability to target multiple mechanisms within the cell to control carcinogenesis. Pro-apoptotic, anti-inflammatory and histone modulation are some of the known and crucial mechanisms by which SFN exerts chemoprevention. In addition to pre-clinical experiments, SFN has demonstrated promising results also in clinical studies in woman with breast ductal carcinoma in situ (DCIS) [72], glioblastoma cells [38] and prostate cancer [73]. Moreover, it was found that people who consumed cruciferous vegetables at least once a week have reduced risk of pharynx, oesophageal, colorectal, breast, oral cavity and kidney cancers [74]. Recently, the effect of SFN on cancer stem cell is also another emerging area of interest that may contribute to its chemopreventive properties [75]. Moreover, combination therapies that associate SFN with other therapeutic agents support a possible treatment modality for prostate and colorectal cancer [76, 77]. SFN combination with agents other than chemotherapeutics has bee report to play an important role also in bladder cancer and bronchial carcinoid cell lines [76, 78]. Furthermore, SFN is highly tolerable and safe in humans with no genotoxic effect [79]. However, more clinical studies are necessary to fully explore the efficacy of SFN as combination therapy, anti-cancer agent or chemopreventive in humans.

In conclusion, for the first time, our data highlight the effects of SFN on melanoma gene expression profiles and elucidates the mechanisms by which it exerts anticancer activity by suppressing various critical hallmarks of cancer, such as cell growth and proliferation, apoptosis, invasion and migration. Our data provides a global view of the potential complex and concurrent mechanisms by which SFN may fight against melanoma and serve as a resource for future investigations. Our results confirm the favourable toxicological profile and high potential for chemotherapeutic activity of SFN. Moreover, our data indicate that SFN is an attractive multipotent antitumour agent for melanoma therapy, and it could open new avenues for the prevention of tumour progression and/or treatment of human malignancies.

## Electronic supplementary material

Below is the link to the electronic supplementary material.

Supplementary material 1 (PDF 489 kb)
