# Supplementary material for: Antitumor activity and expression profiles of genes induced by sulforaphane in human melanoma cells
Source: Eur J Nutr. 2017 Sep 1;57(7):2547–69. doi: 10.1007/s00394-017-1527-7 (PMC6182666; doi:10.1007/s00394-017-1527-7)
Supplement: Supplementary file 1 — Supplementary material 1 (PDF 489 kb) [file 394_2017_1527_MOESM1_ESM.pdf]

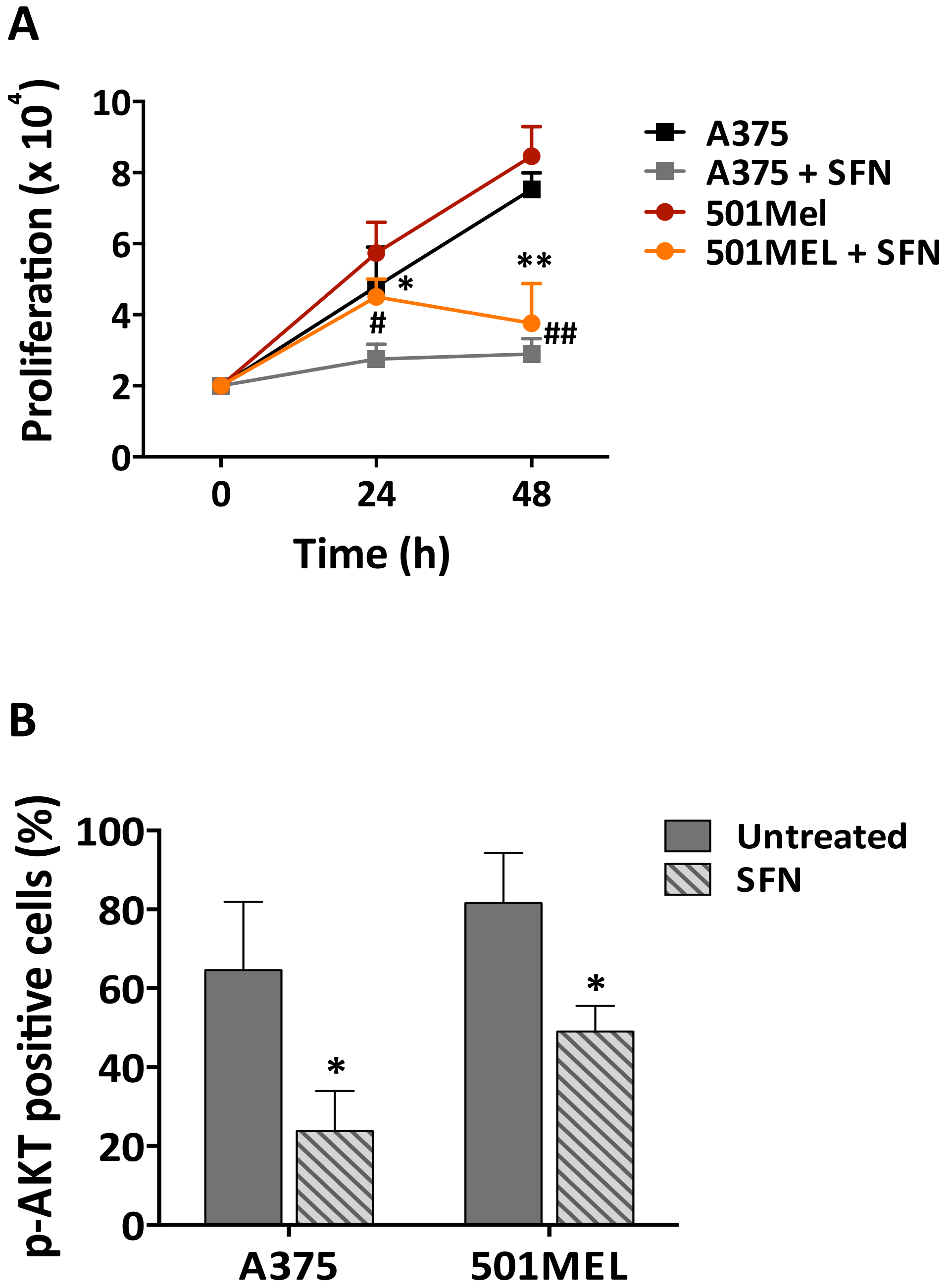

Figure S1. SFN reduces cell viability and proliferation. (A) A375 and 501MEL treated with 2 µg/ml of SFN were stained with Trypan Blue and counted by microscopy. *:501MEL and #: A375 indicate the statistical analysis verso untreated cells at the same time points (($p<0.05$ (*) $p<0.01$ (**)).
(B) p-AKT positive cells were evaluated by FACS analysis at 24 h post treatment. Results are representative of three independent experiments ± SD. The statistical analysis was performed by paired two-tailed Student's test.

Table S1. Up-regulated and down-regulated genes in A375 cells during cell growth in culture (without compound treatment).

Gene Name and Symbol	Fold change
E74 Like ETS Transcription Factor 3 (ELF3)	7,66
Transmembrane Protein 74B (TMEM74B)	5,70
Uroplakin 1A (UPK1A)	5,46
Carbonic Anhydrase 9 (CA9)	4,85
Thioredoxin Interacting Protein (TXNIP)	4,52
Prostate Cancer Associated Transcript 6 (Non-Protein Coding) (KDM5B-AS1)	4,35
UPK1A Antisense RNA 1 (UPK1A-AS1)	4,31
N-Myc Downstream Regulated 1 (NDRG1)	4,26
Thrombospondin-Type Laminin G Domain And EAR Repeats (TSPEAR)	4,05
Neuritin 1 (NRN1)	4,05
MIR210 Host Gene (MIR210HG)	3,98
Chromosome 4 Open Reading Frame 47 (C4orf47)	3,64
PTPRF Interacting Protein Alpha 4 (PPFIA4)	3,61
Zona Pellucida Glycoprotein 1 (ZP1)	3,48
Ribosomal Modification Protein RimK-Like Family Member A (RIMKLA)	3,37
Transmembrane Protein 45A (TMEM45A)	3,28
Espin Pseudogene (ESPNP)	3,28
SH3 Domain Containing 21 (SH3D21)	3,19
Aldolase, Fructose-Bisphosphate C (ALDOC)	3,07
Stanniocalcin 1 (STC1)	3,07
TSPEAR Antisense RNA 2 (C21orf90)	3,05
Sperm Associated Antigen 4 (SPAG4)	2,98
ArfGAP With Coiled-Coil, Ankyrin Repeat And PH Domains 1 (ACAP1)	2,82
Enolase 2 (ENO2)	2,75
Rho Guanine Nucleotide Exchange Factor 37 (ARHGEF37)	2,65
Ephrin A3 (EFNA3)	2,62
BCL2/Adenovirus E1B 19kDa Interacting Protein 3 (BNIP3)	2,57
Protein Phosphatase 1 Regulatory Subunit 3B (PPP1R3B)	2,57
Basic Helix-Loop-Helix Family Member E40 (BHLHE40)	2,56
6-Phosphofructo-2-Kinase/Fructose-2,6-Biphosphatase 4 (PFKFB4)	2,50
Obscurin, Cytoskeletal Calmodulin And Titin-Interacting RhoGEF (OBSCN)	2,45
Protein Tyrosine Phosphatase, Receptor Type B (PTPRB)	2,43
Endothelial PAS Domain Protein 1 (EPAS1)	2,33
Myosin XVB (MYO15B)	2,18
Intercellular Adhesion Molecule 5 (ICAM5)	2,17
ORAI Calcium Release-Activated Calcium Modulator 3 (ORAI3)	2,16
Potassium Channel Tetramerization Domain Containing 11 (KCTD11)	2,15
AHNAK Nucleoprotein 2 (AHNAK2)	2,11
MAX Interactor 1, Dimerization Protein (MXI1)	2,07
Nuclear Factor Of Activated T-Cells 4 (NFATC4)	2,07
Ankyrin Repeat Domain 37 (ANKRD37)	2,06
Cyclin G2 (CCNG2)	1,92
Heparan Sulfate Proteoglycan 2 (HSPG2)	1,90

Perilipin 2 (PLIN2)	1,89
Discs Large Homolog 4 (DLG4)	1,84
Insulin Induced Gene 2 (INSIG2)	1,78
Nuclear Paraspeckle Assembly Transcript 1 (Non-Protein Coding) (NEAT1)	1,78
Aldehyde Dehydrogenase 3 Family Member B1 (ALDH3B1)	1,78
Thrombospondin Type 1 Domain Containing 4 (THSD4)	1,77
Pyruvate Dehydrogenase Kinase 1 (PDK1)	1,75
ATP Binding Cassette Subfamily C Member 3 (ABBC3)	1,72
Prolyl 4-Hydroxylase Subunit Alpha 1 (P4HA1)	1,71
Peptidylglycine Alpha-Amidating Monooxygenase (PAM)	1,71
Inositol 1,4,5-Trisphosphate Receptor Type 1 (ITPR1)	1,70
ATP Binding Cassette Subfamily A Member 2 (ABCA2)	1,67
Dedicator Of Cytokinesis 6 (DOCK6)	1,66
Zinc Finger Protein 395 (ZNF395)	1,66
Mannose Phosphate Isomerase (MPI)	1,65
ABI Family Member 3 Binding Protein (ABI3BP)	1,58
BCL2/Adenovirus E1B 19kDa Interacting Protein 3-Like (BNIP3L)	1,58
Phosphoglycerate Kinase 1 (PGK1)	1,57
Branched Chain Keto Acid Dehydrogenase E1, Alpha Polypeptide (BCKDHA)	1,56
Lysine Demethylase 3A (KDM3A)	1,53
Oncostatin M Receptor (OSMR)	1,51
Glucan (1,4-Alpha-), Branching Enzyme 1 (GBE1)	1,50
Family With Sequence Similarity 162 Member A (FAM162A)	1,49
Phosphofructokinase, Liver Type (PFKL)	1,49
DNA Damage Inducible Transcript 4 (DDIT4)	1,48
Ankyrin Repeat And Zinc Finger Domain Containing 1 (ANKZF1)	1,45
Chromosome 4 Open Reading Frame 3 (C4orf3)	1,45
Zinc Finger Protein 292 (ZNF292)	1,44
Hexokinase 2 (HK2)	1,42
Agrin (AGRN)	1,34
Laminin Subunit Beta 1 (LAMB1)	1,33
5'-Nucleotidase Ecto (NT5E)	1,33
EPS8 Like 2 (EPS8L2)	1,18
Heat Shock Protein 90kDa Alpha Family Class A Member 1 (HSP90AA1)	-1,17
Heterogeneous Nuclear Ribonucleoprotein A/B (HNRNPAB)	-1,17
Cysteine And Histidine Rich Domain Containing 1 (CHORDC1)	-1,22
Alanyl-tRNA synthetase (AARS)	-1,23
Cell Division Cycle 25A (CDC25A)	-1,26
Tyrosyl-TRNA Synthetase (YARS)	-1,28
H2A Histone Family Member Z (H2AFZ)	-1,28
Ribonucleotide Reductase Regulatory Subunit M2 (RRM2)	-1,30
Cytochrome C, Somatic (CYCS)	-1,32
WD Repeat Domain 77 (WDR77)	-1,32
Flap Structure-Specific Endonuclease 1 (FEN1)	-1,33
Cell Division Cycle 6 (CDC6)	-1,34
Acireductone Dioxygenase 1 (ADI1)	-1,35
Torsin Family 3 Member A (TOR3A)	-1,36

Solute Carrier Family 7 Member 11 (SLC7A11)	-1,38
MYB Proto-Oncogene Like 2 (MYBL2)	-1,40
Cyclin E2 (CCNE2)	-1,40
Geminin, DNA Replication Inhibitor (GMNN)	-1,41
Minichromosome Maintenance 10 Replication Initiation Factor (MCM10)	-1,46
Cyclin D3 (CCND3)	-1,46
Heat Shock Protein Family A (Hsp70) Member 8 (HSPA8)	-1,48
DNA Replication And Sister Chromatid Cohesion 1 (DSCC1)	-1,51
DnaJ Heat Shock Protein Family (Hsp40) Member A1 (DNAJA1)	-1,54
Phosphoserine Phosphatase (PSPH)	-1,54
Proliferating Cell Nuclear Antigen (PCNA)	-1,57
Methionyl-TRNA Synthetase (MARS)	-1,60
Claspin (CLSPN)	-1,63
Heat Shock Protein Family H (Hsp110) Member 1 (HSPH1)	-1,66
MPV17 Mitochondrial Inner Membrane Protein Like 2 (MPV17L2)	-1,68
Transferrin Receptor (TFRC)	-1,75
Tryptophanyl-TRNA Synthetase (WARS)	-1,87
Heat Shock Protein Family A (Hsp70) Member 1A (HSPA1A)	-1,96
Solute Carrier Family 6 Member 9 (SLC6A9)	-2,10
Heat Shock Protein Family A (Hsp70) Member 1B (HSPA1B)	-2,15

Table S2. The results for each enriched GO category are listed in this table.

Ensembl Gene Stable ID	Gene symbol	Description	Entrez ID
**Biological process**	**Response to stress**	**GO:0006950**	
C=2825; O=86; E=38.03; R=2.26; rawP=1.50e-15; adjP=2.43e-12			
ENSG00000095752	IL11	interleukin 11	3589
ENSG00000162616	DNAJB4	DnaJ (Hsp40) homolog, subfamily B, member 4	11080
ENSG00000091592	NLRP1	NLR family, pyrin domain containing 1	22861
ENSG00000134986	NREP	neuronal regeneration related protein homolog (rat)	9315
ENSG00000124762	CDKN1A	cyclin-dependent kinase inhibitor 1A (p21, Cip1)	1026
ENSG00000100292	HMOX1	heme oxygenase (decycling) 1	3162
ENSG00000104368	PLAT	plasminogen activator, tissue	5327
ENSG00000112319	EYA4	eyes absent homolog 4 (Drosophila)	2070
ENSG00000135679	MDM2	Mdm2, p53 E3 ubiquitin protein ligase homolog (mouse)	4193
ENSG00000070669	ASNS	asparagine synthetase (glutamine-hydrolyzing)	440
ENSG00000105993	DNAJB6	DnaJ (Hsp40) homolog, subfamily B, member 6	10049
ENSG00000128016	ZFP36	zinc finger protein 36, C3H type, homolog (mouse)	7538
ENSG00000181104	F2R	coagulation factor II (thrombin) receptor	2149
ENSG00000239713	APOBEC3G	apolipoprotein B mRNA editing enzyme, catalytic polypeptide-like 3G	60489
ENSG00000116761	CTH	cystathionase (cystathionine gamma-lyase)	1491
ENSG00000173846	PLK3	polo-like kinase 3	1263
ENSG00000196352	CD55	CD55 molecule, decay accelerating factor for complement (Cromer blood group)	1604
ENSG00000142168	SOD1	superoxide dismutase 1, soluble	6647
ENSG00000185088	RPS27L	ribosomal protein S27-like	51065
ENSG00000132170	PPARG	peroxisome proliferator-activated receptor gamma	5468
ENSG00000159388	BTG2	BTG family, member 2	7832
ENSG00000110172	CHORDC1	cysteine and histidine-rich domain (CHORD) containing 1	26973
ENSG00000197903	HIST1H2BK	histone cluster 1, H2bk	85236
ENSG00000162772	ATF3	activating transcription factor 3	467
ENSG00000086061	DNAJA1	DnaJ (Hsp40) homolog, subfamily A, member 1	3301
ENSG00000138413	IDH1	isocitrate dehydrogenase 1 (NADP+), soluble	3417
ENSG00000080546	SESN1	sestrin 1	27244
ENSG00000173110	HSPA6	heat shock 70kDa protein 6 (HSP70B')	3310
ENSG00000160255	ITGB2	integrin, beta 2 (complement component 3 receptor 3 and 4 subunit)	3689
ENSG00000119630	PGF	placental growth factor	5228
ENSG00000204390	HSPA1L	heat shock 70kDa protein 1-like	3305
ENSG00000186480	INSIG1	insulin induced gene 1	3638
ENSG00000160211	G6PD	glucose-6-phosphate dehydrogenase	2539
ENSG00000115919	KYNU	kynureninase	8942
ENSG00000099860	GADD45B	growth arrest and DNA-damage-inducible, beta	4616
ENSG00000152137	HSPB8	heat shock 22kDa protein 8	26353

ENSG00000105327	BBC3	BCL2 binding component 3	27113
ENSG00000120885	CLU	clusterin	1191
ENSG00000120694	HSPH1	heat shock 105kDa/110kDa protein 1	10808
ENSG00000156273	BACH1	BTB and CNC homology 1, basic leucine zipper transcription factor 1	571
ENSG00000023909	GCLM	glutamate-cysteine ligase, modifier subunit	2730
ENSG00000116299	KIAA1324	KIAA1324	57535
ENSG00000131080	EDA2R	ectodysplasin A2 receptor	60401
ENSG00000120738	EGR1	early growth response 1	1958
ENSG00000074590	NUAK1	NUAK family, SNF1-like kinase, 1	9891
ENSG00000164938	TP53INP1	tumor protein p53 inducible nuclear protein 1	94241
ENSG00000160200	CBS	cystathionine-beta-synthase	875
ENSG00000132002	DNAJB1	DnaJ (Hsp40) homolog, subfamily B, member 1	3337
ENSG00000048052	HDAC9	histone deacetylase 9	9734
ENSG00000168209	DDIT4	DNA-damage-inducible transcript 4	54541
ENSG00000085276	MECOM	MDS1 and EVI1 complex locus	2122
ENSG00000161011	SQSTM1	sequestosome 1	8878
ENSG00000136560	TANK	TRAF family member-associated NFKB activator	10010
ENSG00000170855	TRIAP1	TP53 regulated inhibitor of apoptosis 1	51499
ENSG00000087088	BAX	BCL2-associated X protein	581
ENSG00000010278	CD9	CD9 molecule	928
ENSG00000072310	SREBF1	sterol regulatory element binding transcription factor 1	6720
ENSG00000080824	HSP90AA1	heat shock protein 90kDa alpha (cytosolic), class A member 1	3320
ENSG00000116285	ERRFI1	ERBB receptor feedback inhibitor 1	54206
ENSG00000184995	IFNE	interferon, epsilon	338376
ENSG00000198431	TXNRD1	thioredoxin reductase 1	7296
ENSG00000134574	DDB2	damage-specific DNA binding protein 2, 48kDa	1643
ENSG00000001084	GCLC	glutamate-cysteine ligase, catalytic subunit	2729
ENSG00000181222	POLR2A	polymerase (RNA) II (DNA directed) polypeptide A, 220kDa	5430
ENSG00000179583	CIITA	class II, major histocompatibility complex, transactivator	4261
ENSG00000204388	HSPA1B	heat shock 70kDa protein 1B	3304
ENSG00000026103	FAS	Fas (TNF receptor superfamily, member 6)	355
ENSG00000135047	CTSL1	cathepsin L1	1514
ENSG00000121691	CAT	catalase	847
ENSG00000117318	ID3	inhibitor of DNA binding 3, dominant negative helix-loop-helix protein	3399
ENSG00000065911	MTHFD2	methylenetetrahydrofolate dehydrogenase (NADP+ dependent) 2, methenyltetrahydrofolate cyclohydrolase	10797
ENSG00000116717	GADD45A	growth arrest and DNA-damage-inducible, alpha	1647
ENSG00000087074	PPP1R15A	protein phosphatase 1, regulatory subunit 15A	23645
ENSG00000153879	CEBPG	CCAAT/enhancer binding protein (C/EBP), gamma	1054
ENSG00000181026	AEN	apoptosis enhancing nuclease	64782
ENSG00000255150	EID3	EP300 interacting inhibitor of differentiation 3	493861
ENSG00000132470	ITGB4	integrin, beta 4	3691
ENSG00000174307	PHLDA3	pleckstrin homology-like domain, family A, member 3	23612
ENSG00000105939	ZC3HAV1	zinc finger CCCH-type, antiviral 1	56829

Biological process	Apoptotic process	GO:0006915	
C=1470;O=57;E=19.79;R=2.88;rawP=4.88e-14;adjP=3.44e-11			
ENSG00000023909	GCLM	glutamate-cysteine ligase, modifier subunit	2730
ENSG00000116299	KIAA1324	KIAA1324	57535
ENSG00000091592	NLRP1	NLR family, pyrin domain containing 1	22861
ENSG00000164938	TP53INP1	tumor protein p53 inducible nuclear protein 1	94241
ENSG00000146072	TNFRSF21	tumor necrosis factor receptor superfamily, member 21	27242
ENSG00000124762	CDKN1A	cyclin-dependent kinase inhibitor 1A (p21, Cip1)	1026
ENSG00000100292	HMOX1	heme oxygenase (decycling) 1	3162
ENSG00000139289	PHLDA1	pleckstrin homology-like domain, family A, member 1	22822
ENSG00000125657	TNFSF9	tumor necrosis factor (ligand) superfamily, member 9	8744
ENSG00000100625	SIX4	SIX homeobox 4	51804
ENSG00000196072	BLOC1S2	biogenesis of lysosomal organelles complex-1, subunit 2	282991
ENSG00000085276	MECOM	MDS1 and EVI1 complex locus	2122
ENSG00000168209	DDIT4	DNA-damage-inducible transcript 4	54541
ENSG00000198121	LPAR1	lysophosphatidic acid receptor 1	1902
ENSG00000151929	BAG3	BCL2-associated athanogene 3	9531
ENSG00000135679	MDM2	Mdm2, p53 E3 ubiquitin protein ligase homolog (mouse)	4193
ENSG00000070669	ASNS	asparagine synthetase (glutamine-hydrolyzing)	440
ENSG00000161011	SQSTM1	sequestosome 1	8878
ENSG00000105993	DNAJB6	DnaJ (Hsp40) homolog, subfamily B, member 6	10049
ENSG00000170855	TRIAP1	TP53 regulated inhibitor of apoptosis 1	51499
ENSG00000162892	IL24	interleukin 24	11009
ENSG00000184254	ALDH1A3	aldehyde dehydrogenase 1 family, member A3	220
ENSG00000087088	BAX	BCL2-associated X protein	581
ENSG00000006327	TNFRSF12A	tumor necrosis factor receptor superfamily, member 12A	51330
ENSG00000181104	F2R	coagulation factor II (thrombin) receptor	2149
ENSG00000240694	PNMA2	paraneoplastic Ma antigen 2	10687
ENSG00000146674	IGFBP3	insulin-like growth factor binding protein 3	3486
ENSG00000108179	PPIF	peptidylprolyl isomerase F	10105
ENSG00000173846	PLK3	polo-like kinase 3	1263
ENSG00000162734	PEA15	phosphoprotein enriched in astrocytes 15	8682
ENSG00000142168	SOD1	superoxide dismutase 1, soluble	6647
ENSG00000185088	RPS27L	ribosomal protein S27-like	51065
ENSG00000132170	PPARG	peroxisome proliferator-activated receptor gamma	5468
ENSG00000001084	GCLC	glutamate-cysteine ligase, catalytic subunit	2729
ENSG00000159388	BTG2	BTG family, member 2	7832
ENSG00000115641	FHL2	four and a half LIM domains 2	2274
ENSG00000204388	HSPA1B	heat shock 70kDa protein 1B	3304
ENSG00000026103	FAS	Fas (TNF receptor superfamily, member 6)	355
ENSG00000135047	CTSL1	cathepsin L1	1514
ENSG00000160255	ITGB2	integrin, beta 2 (complement component 3 receptor 3 and 4 subunit)	3689
ENSG00000121691	CAT	catalase	847
ENSG00000117318	ID3	inhibitor of DNA binding 3, dominant negative helix-loop-helix protein	3399

ENSG00000116717	GADD45A	growth arrest and DNA-damage-inducible, alpha	1647
ENSG00000142627	EPHA2	EPH receptor A2	1969
ENSG00000087074	PPP1R15A	protein phosphatase 1, regulatory subunit 15A	23645
ENSG00000099860	GADD45B	growth arrest and DNA-damage-inducible, beta	4616
ENSG00000181026	AEN	apoptosis enhancing nuclease	64782
ENSG00000105327	BBC3	BCL2 binding component 3	27113
ENSG00000120885	CLU	clusterin	1191
ENSG00000174307	PHLDA3	pleckstrin homology-like domain, family A, member 3	23612
ENSG00000160570	DEDD2	death effector domain containing 2	162989
ENSG00000136002	ARHGEF4	Rho guanine nucleotide exchange factor (GEF) 4	50649
**Biological process**	**Death**	**GO:0016265**	
C=1639;O=60;E=22.06;R=2.72;rawP=1.06e-13;adjP=3.44e-11			
ENSG00000023909	GCLM	glutamate-cysteine ligase, modifier subunit	2730
ENSG00000116299	KIAA1324	KIAA1324	57535
ENSG00000091592	NLRP1	NLR family, pyrin domain containing 1	22861
ENSG00000164938	TP53INP1	tumor protein p53 inducible nuclear protein 1	94241
ENSG00000146072	TNFRSF21	tumor necrosis factor receptor superfamily, member 21	27242
ENSG00000124762	CDKN1A	cyclin-dependent kinase inhibitor 1A (p21, Cip1)	1026
ENSG00000128965	CHAC1	ChaC, cation transport regulator homolog 1 (E. coli)	79094
ENSG00000100292	HMOX1	heme oxygenase (decycling) 1	3162
ENSG00000139289	PHLDA1	pleckstrin homology-like domain, family A, member 1	22822
ENSG00000125657	TNFSF9	tumor necrosis factor (ligand) superfamily, member 9	8744
ENSG00000100625	SIX4	SIX homeobox 4	51804
ENSG00000196072	BLOC1S2	biogenesis of lysosomal organelles complex-1, subunit 2	282991
ENSG00000085276	MECOM	MDS1 and EVI1 complex locus	2122
ENSG00000168209	DDIT4	DNA-damage-inducible transcript 4	54541
ENSG00000198121	LPAR1	lysophosphatidic acid receptor 1	1902
ENSG00000151929	BAG3	BCL2-associated athanogene 3	9531
ENSG00000135679	MDM2	Mdm2, p53 E3 ubiquitin protein ligase homolog (mouse)	4193
ENSG00000070669	ASNS	asparagine synthetase (glutamine-hydrolyzing)	440
ENSG00000161011	SQSTM1	sequestosome 1	8878
ENSG00000105993	DNAJB6	DnaJ (Hsp40) homolog, subfamily B, member 6	10049
ENSG00000170855	TRIAP1	TP53 regulated inhibitor of apoptosis 1	51499
ENSG00000162892	IL24	interleukin 24	11009
ENSG00000184254	ALDH1A3	aldehyde dehydrogenase 1 family, member A3	220
ENSG00000087088	BAX	BCL2-associated X protein	581
ENSG00000006327	TNFRSF12A	tumor necrosis factor receptor superfamily, member 12A	51330
ENSG00000181104	F2R	coagulation factor II (thrombin) receptor	2149
ENSG00000240694	PNMA2	paraneoplastic Ma antigen 2	10687
ENSG00000146674	IGFBP3	insulin-like growth factor binding protein 3	3486
ENSG00000108179	PPIF	peptidylprolyl isomerase F	10105
ENSG00000173846	PLK3	polo-like kinase 3	1263
ENSG00000162734	PEA15	phosphoprotein enriched in astrocytes 15	8682
ENSG00000106105	GARS	glycyl-tRNA synthetase	2617
ENSG00000142168	SOD1	superoxide dismutase 1, soluble	6647
ENSG00000185088	RPS27L	ribosomal protein S27-like	51065

ENSG00000132170	PPARG	peroxisome proliferator-activated receptor gamma	5468
ENSG00000001084	GCLC	glutamate-cysteine ligase, catalytic subunit	2729
ENSG00000159388	BTG2	BTG family, member 2	7832
ENSG00000115641	FHL2	four and a half LIM domains 2	2274
ENSG00000204388	HSPA1B	heat shock 70kDa protein 1B	3304
ENSG00000026103	FAS	Fas (TNF receptor superfamily, member 6)	355
ENSG00000135047	CTSL1	cathepsin L1	1514
ENSG00000160255	ITGB2	integrin, beta 2 (complement component 3 receptor 3 and 4 subunit)	3689
ENSG00000121691	CAT	catalase	847
ENSG00000117318	ID3	inhibitor of DNA binding 3, dominant negative helix-loop-helix protein	3399
ENSG00000116717	GADD45A	growth arrest and DNA-damage-inducible, alpha	1647
ENSG00000142627	EPHA2	EPH receptor A2	1969
ENSG00000087074	PPP1R15A	protein phosphatase 1, regulatory subunit 15A	23645
ENSG00000099860	GADD45B	growth arrest and DNA-damage-inducible, beta	4616
ENSG00000152137	HSPB8	heat shock 22kDa protein 8	26353
ENSG00000181026	AEN	apoptosis enhancing nuclease	64782
ENSG00000105327	BBC3	BCL2 binding component 3	27113
ENSG00000120885	CLU	clusterin	1191
ENSG00000174307	PHLDA3	pleckstrin homology-like domain, family A, member 3	23612
ENSG00000160570	DEDD2	death effector domain containing 2	162989
ENSG00000136002	ARHGEF4	Rho guanine nucleotide exchange factor (GEF) 4	50649
**Biological process**	**Programmed cell death**	**GO:0012501**	
C=1485;O=57;E=19.99;R=2.85;rawP=7.55e-14;adjP=3.44e-11			
ENSG00000023909	GCLM	glutamate-cysteine ligase, modifier subunit	2730
ENSG00000116299	KIAA1324	KIAA1324	57535
ENSG00000091592	NLRP1	NLR family, pyrin domain containing 1	22861
ENSG00000164938	TP53INP1	tumor protein p53 inducible nuclear protein 1	94241
ENSG00000146072	TNFRSF21	tumor necrosis factor receptor superfamily, member 21	27242
ENSG00000124762	CDKN1A	cyclin-dependent kinase inhibitor 1A (p21, Cip1)	1026
ENSG00000128965	CHAC1	ChaC, cation transport regulator homolog 1 (E. coli)	79094
ENSG00000100292	HMOX1	heme oxygenase (decycling) 1	3162
ENSG00000139289	PHLDA1	pleckstrin homology-like domain, family A, member 1	22822
ENSG00000125657	TNFSF9	tumor necrosis factor (ligand) superfamily, member 9	8744
ENSG00000100625	SIX4	SIX homeobox 4	51804
ENSG00000196072	BLOC1S2	biogenesis of lysosomal organelles complex-1, subunit 2	282991
ENSG00000085276	MECOM	MDS1 and EVI1 complex locus	2122
ENSG00000168209	DDIT4	DNA-damage-inducible transcript 4	54541
ENSG00000198121	LPAR1	lysophosphatidic acid receptor 1	1902
ENSG00000151929	BAG3	BCL2-associated athanogene 3	9531
ENSG00000135679	MDM2	Mdm2, p53 E3 ubiquitin protein ligase homolog (mouse)	4193
ENSG00000070669	ASNS	asparagine synthetase (glutamine-hydrolyzing)	440
ENSG00000161011	SQSTM1	sequestosome 1	8878
ENSG00000105993	DNAJB6	DnaJ (Hsp40) homolog, subfamily B, member 6	10049
ENSG00000170855	TRIAP1	TP53 regulated inhibitor of apoptosis 1	51499

ENSG00000162892	IL24	interleukin 24	11009
ENSG00000184254	ALDH1A3	aldehyde dehydrogenase 1 family, member A3	220
ENSG00000087088	BAX	BCL2-associated X protein	581
ENSG00000006327	TNFRSF12A	tumor necrosis factor receptor superfamily, member 12A	51330
ENSG00000181104	F2R	coagulation factor II (thrombin) receptor	2149
ENSG00000240694	PNMA2	paraneoplastic Ma antigen 2	10687
ENSG00000146674	IGFBP3	insulin-like growth factor binding protein 3	3486
ENSG00000108179	PPIF	peptidylprolyl isomerase F	10105
ENSG00000173846	PLK3	polo-like kinase 3	1263
ENSG00000162734	PEA15	phosphoprotein enriched in astrocytes 15	8682
ENSG00000142168	SOD1	superoxide dismutase 1, soluble	6647
ENSG00000185088	RPS27L	ribosomal protein S27-like	51065
ENSG00000132170	PPARG	peroxisome proliferator-activated receptor gamma	5468
ENSG00000001084	GCLC	glutamate-cysteine ligase, catalytic subunit	2729
ENSG00000159388	BTG2	BTG family, member 2	7832
ENSG00000115641	FHL2	four and a half LIM domains 2	2274
ENSG00000204388	HSPA1B	heat shock 70kDa protein 1B	3304
ENSG00000026103	FAS	Fas (TNF receptor superfamily, member 6)	355
ENSG00000135047	CTSL1	cathepsin L1	1514
ENSG00000160255	ITGB2	integrin, beta 2 (complement component 3 receptor 3 and 4 subunit)	3689
ENSG00000121691	CAT	catalase	847
ENSG00000117318	ID3	inhibitor of DNA binding 3, dominant negative helix-loop-helix protein	3399
ENSG00000116717	GADD45A	growth arrest and DNA-damage-inducible, alpha	1647
ENSG00000142627	EPHA2	EPH receptor A2	1969
ENSG00000087074	PPP1R15A	protein phosphatase 1, regulatory subunit 15A	23645
ENSG00000099860	GADD45B	growth arrest and DNA-damage-inducible, beta	4616
ENSG00000181026	AEN	apoptosis enhancing nuclease	64782
ENSG00000105327	BBC3	BCL2 binding component 3	27113
ENSG00000120885	CLU	clusterin	1191
ENSG00000174307	PHLDA3	pleckstrin homology-like domain, family A, member 3	23612
ENSG00000160570	DEDD2	death effector domain containing 2	162989
ENSG00000136002	ARHGEF4	Rho guanine nucleotide exchange factor (GEF) 4	50649
**Biological process**	**Cell death**	**GO:0008219**	
C=1637;O=60;E=22.04;R=2.72;rawP=1.00e-13;adjP=3.44e-11			
ENSG00000023909	GCLM	glutamate-cysteine ligase, modifier subunit	2730
ENSG00000116299	KIAA1324	KIAA1324	57535
ENSG00000091592	NLRP1	NLR family, pyrin domain containing 1	22861
ENSG00000164938	TP53INP1	tumor protein p53 inducible nuclear protein 1	94241
ENSG00000146072	TNFRSF21	tumor necrosis factor receptor superfamily, member 21	27242
ENSG00000124762	CDKN1A	cyclin-dependent kinase inhibitor 1A (p21, Cip1)	1026
ENSG00000100292	HMOX1	heme oxygenase (decycling) 1	3162
ENSG00000139289	PHLDA1	pleckstrin homology-like domain, family A, member 1	22822
ENSG00000125657	TNFSF9	tumor necrosis factor (ligand) superfamily, member 9	8744
ENSG00000100625	SIX4	SIX homeobox 4	51804

ENSG00000196072	BLOC1S2	biogenesis of lysosomal organelles complex-1, subunit 2	282991
ENSG00000085276	MECOM	MDS1 and EVI1 complex locus	2122
ENSG00000168209	DDIT4	DNA-damage-inducible transcript 4	54541
ENSG00000198121	LPAR1	lysophosphatidic acid receptor 1	1902
ENSG00000151929	BAG3	BCL2-associated athanogene 3	9531
ENSG00000135679	MDM2	Mdm2, p53 E3 ubiquitin protein ligase homolog (mouse)	4193
ENSG00000070669	ASNS	asparagine synthetase (glutamine-hydrolyzing)	440
ENSG00000161011	SQSTM1	sequestosome 1	8878
ENSG00000105993	DNAJB6	DnaJ (Hsp40) homolog, subfamily B, member 6	10049
ENSG00000170855	TRIAP1	TP53 regulated inhibitor of apoptosis 1	51499
ENSG00000162892	IL24	interleukin 24	11009
ENSG00000184254	ALDH1A3	aldehyde dehydrogenase 1 family, member A3	220
ENSG00000087088	BAX	BCL2-associated X protein	581
ENSG00000006327	TNFRSF12A	tumor necrosis factor receptor superfamily, member 12A	51330
ENSG00000181104	F2R	coagulation factor II (thrombin) receptor	2149
ENSG00000240694	PNMA2	paraneoplastic Ma antigen 2	10687
ENSG00000146674	IGFBP3	insulin-like growth factor binding protein 3	3486
ENSG00000108179	PPIF	peptidylprolyl isomerase F	10105
ENSG00000173846	PLK3	polo-like kinase 3	1263
ENSG00000162734	PEA15	phosphoprotein enriched in astrocytes 15	8682
ENSG00000106105	GARS	glycyl-tRNA synthetase	2617
ENSG00000142168	SOD1	superoxide dismutase 1, soluble	6647
ENSG00000185088	RPS27L	ribosomal protein S27-like	51065
ENSG00000132170	PPARG	peroxisome proliferator-activated receptor gamma	5468
ENSG00000001084	GCLC	glutamate-cysteine ligase, catalytic subunit	2729
ENSG00000159388	BTG2	BTG family, member 2	7832
ENSG00000115641	FHL2	four and a half LIM domains 2	2274
ENSG00000204388	HSPA1B	heat shock 70kDa protein 1B	3304
ENSG00000026103	FAS	Fas (TNF receptor superfamily, member 6)	355
ENSG00000135047	CTSL1	cathepsin L1	1514
ENSG00000160255	ITGB2	integrin, beta 2 (complement component 3 receptor 3 and 4 subunit)	3689
ENSG00000121691	CAT	catalase	847
ENSG00000117318	ID3	inhibitor of DNA binding 3, dominant negative helix-loop-helix protein	3399
ENSG00000116717	GADD45A	growth arrest and DNA-damage-inducible, alpha	1647
ENSG00000142627	EPHA2	EPH receptor A2	1969
ENSG00000087074	PPP1R15A	protein phosphatase 1, regulatory subunit 15A	23645
ENSG00000099860	GADD45B	growth arrest and DNA-damage-inducible, beta	4616
ENSG00000152137	HSPB8	heat shock 22kDa protein 8	26353
ENSG00000181026	AEN	apoptosis enhancing nuclease	64782
ENSG00000105327	BBC3	BCL2 binding component 3	27113
ENSG00000120885	CLU	clusterin	1191
ENSG00000174307	PHLDA3	pleckstrin homology-like domain, family A, member 3	23612
ENSG00000160570	DEDD2	death effector domain containing 2	162989
ENSG00000136002	ARHGEF4	Rho guanine nucleotide exchange factor (GEF) 4	50649

**Biological process**	**Response to topologically incorrect protein**	**GO:0035966**	
C=136;O=16;E=1.83;R=8.74;rawP=3.87e-11;adjP=9.47e-09			
ENSG00000120694	HSPH1	heat shock 105kDa/110kDa protein 1	10808
ENSG00000162616	DNAJB4	DnaJ (Hsp40) homolog, subfamily B, member 4	11080
ENSG00000162772	ATF3	activating transcription factor 3	467
ENSG00000086061	DNAJA1	DnaJ (Hsp40) homolog, subfamily A, member 1	3301
ENSG00000132002	DNAJB1	DnaJ (Hsp40) homolog, subfamily B, member 1	3337
ENSG00000204388	HSPA1B	heat shock 70kDa protein 1B	3304
ENSG00000173110	HSPA6	heat shock 70kDa protein 6 (HSP70B')	3310
ENSG00000204390	HSPA1L	heat shock 70kDa protein 1-like	3305
ENSG00000070669	ASNS	asparagine synthetase (glutamine-hydrolyzing)	440
ENSG00000087074	PPP1R15A	protein phosphatase 1, regulatory subunit 15A	23645
ENSG00000105993	DNAJB6	DnaJ (Hsp40) homolog, subfamily B, member 6	10049
ENSG00000087088	BAX	BCL2-associated X protein	581
ENSG00000120885	CLU	clusterin	1191
ENSG00000080824	HSP90AA1	heat shock protein 90kDa alpha (cytosolic), class A member 1	3320
ENSG00000116761	CTH	cystathionase (cystathionine gamma-lyase)	1491
**Biological process**	**Response to stimulus**	**GO:0050896**	
C=6326;O=129;E=85.16;R=1.51;rawP=4.09e-11;adjP=9.47e-09			
ENSG00000162616	DNAJB4	DnaJ (Hsp40) homolog, subfamily B, member 4	11080
ENSG00000221926	TRIM16	tripartite motif containing 16	10626
ENSG00000091592	NLRP1	NLR family, pyrin domain containing 1	22861
ENSG00000146072	TNFRSF21	tumor necrosis factor receptor superfamily, member 21	27242
ENSG00000109814	UGDH	UDP-glucose 6-dehydrogenase	7358
ENSG00000134986	NREP	neuronal regeneration related protein homolog (rat)	9315
ENSG00000124762	CDKN1A	cyclin-dependent kinase inhibitor 1A (p21, Cip1)	1026
ENSG00000104368	PLAT	plasminogen activator, tissue	5327
ENSG00000171517	LPAR3	lysophosphatidic acid receptor 3	23566
ENSG00000112319	EYA4	eyes absent homolog 4 (Drosophila)	2070
ENSG00000099994	SUSD2	sushi domain containing 2	56241
ENSG00000198121	LPAR1	lysophosphatidic acid receptor 1	1902
ENSG00000128016	ZFP36	zinc finger protein 36, C3H type, homolog (mouse)	7538
ENSG00000184254	ALDH1A3	aldehyde dehydrogenase 1 family, member A3	220
ENSG00000179046	TRIML2	tripartite motif family-like 2	205860
ENSG00000239713	APOBEC3G	apolipoprotein B mRNA editing enzyme, catalytic polypeptide-like 3G	60489
ENSG00000173846	PLK3	polo-like kinase 3	1263
ENSG00000196352	CD55	CD55 molecule, decay accelerating factor for complement (Cromer blood group)	1604
ENSG00000142168	SOD1	superoxide dismutase 1, soluble	6647
ENSG00000185088	RPS27L	ribosomal protein S27-like	51065
ENSG00000132170	PPARG	peroxisome proliferator-activated receptor gamma	5468
ENSG00000153292	GPR110	G protein-coupled receptor 110	266977

ENSG00000197903	HIST1H2BK	histone cluster 1, H2bk	85236
ENSG00000138413	IDH1	isocitrate dehydrogenase 1 (NADP+), soluble	3417
ENSG00000080546	SESN1	sestrin 1	27244
ENSG00000173110	HSPA6	heat shock 70kDa protein 6 (HSP70B')	3310
ENSG00000160255	ITGB2	integrin, beta 2 (complement component 3 receptor 3 and 4 subunit)	3689
ENSG00000119630	PGF	placental growth factor	5228
ENSG00000204390	HSPA1L	heat shock 70kDa protein 1-like	3305
ENSG00000142627	EPHA2	EPH receptor A2	1969
ENSG00000186480	INSIG1	insulin induced gene 1	3638
ENSG00000115919	KYNU	kynureninase	8942
ENSG00000105327	BBC3	BCL2 binding component 3	27113
ENSG00000075223	SEMA3C	sema domain, immunoglobulin domain (Ig), short basic domain, secreted, (semaphorin) 3C	10512
ENSG00000120885	CLU	clusterin	1191
ENSG00000120694	HSPH1	heat shock 105kDa/110kDa protein 1	10808
ENSG00000156273	BACH1	BTB and CNC homology 1, basic leucine zipper transcription factor 1	571
ENSG00000120738	EGR1	early growth response 1	1958
ENSG00000074590	NUAK1	NUAK family, SNF1-like kinase, 1	9891
ENSG00000164938	TP53INP1	tumor protein p53 inducible nuclear protein 1	94241
ENSG00000102048	ASB9	ankyrin repeat and SOCS box containing 9	140462
ENSG00000132002	DNAJB1	DnaJ (Hsp40) homolog, subfamily B, member 1	3337
ENSG00000138166	DUSP5	dual specificity phosphatase 5	1847
ENSG00000168209	DDIT4	DNA-damage-inducible transcript 4	54541
ENSG00000085276	MECOM	MDS1 and EVI1 complex locus	2122
ENSG00000161011	SQSTM1	sequestosome 1	8878
ENSG00000072310	SREBF1	sterol regulatory element binding transcription factor 1	6720
ENSG00000006327	TNFRSF12A	tumor necrosis factor receptor superfamily, member 12A	51330
ENSG00000162734	PEA15	phosphoprotein enriched in astrocytes 15	8682
ENSG00000003137	CYP26B1	cytochrome P450, family 26, subfamily B, polypeptide 1	56603
ENSG00000134574	DDB2	damage-specific DNA binding protein 2, 48kDa	1643
ENSG00000115641	FHL2	four and a half LIM domains 2	2274
ENSG00000179583	CIITA	class II, major histocompatibility complex, transactivator	4261
ENSG00000026103	FAS	Fas (TNF receptor superfamily, member 6)	355
ENSG00000095383	TBC1D2	TBC1 domain family, member 2	55357
ENSG00000121691	CAT	catalase	847
ENSG00000065911	MTHFD2	methylenetetrahydrofolate dehydrogenase (NADP+ dependent) 2, methenyltetrahydrofolate cyclohydrolase	10797
ENSG00000087074	PPP1R15A	protein phosphatase 1, regulatory subunit 15A	23645
ENSG00000153879	CEBPG	CCAAT/enhancer binding protein (C/EBP), gamma	1054
ENSG00000255150	EID3	EP300 interacting inhibitor of differentiation 3	493861
ENSG00000101849	TBL1X	transducin (beta)-like 1X-linked	6907
ENSG00000105939	ZC3HAV1	zinc finger CCCH-type, antiviral 1	56829
ENSG00000095752	IL11	interleukin 11	3589
ENSG00000170485	NPAS2	neuronal PAS domain protein 2	4862
ENSG00000162733	DDR2	discoidin domain receptor tyrosine kinase 2	4921

ENSG00000100292	HMOX1	heme oxygenase (decycling) 1	3162
ENSG00000125657	TNFSF9	tumor necrosis factor (ligand) superfamily, member 9	8744
ENSG00000070669	ASNS	asparagine synthetase (glutamine-hydrolyzing)	440
ENSG00000135679	MDM2	Mdm2, p53 E3 ubiquitin protein ligase homolog (mouse)	4193
ENSG00000105993	DNAJB6	DnaJ (Hsp40) homolog, subfamily B, member 6	10049
ENSG00000181104	F2R	coagulation factor II (thrombin) receptor	2149
ENSG00000063660	GPC1	glypican 1	2817
ENSG00000146674	IGFBP3	insulin-like growth factor binding protein 3	3486
ENSG00000123975	CKS2	CDC28 protein kinase regulatory subunit 2	1164
ENSG00000116761	CTH	cystathionase (cystathionine gamma-lyase)	1491
ENSG00000159388	BTG2	BTG family, member 2	7832
ENSG00000110172	CHORDC1	cysteine and histidine-rich domain (CHORD) containing 1	26973
ENSG00000162772	ATF3	activating transcription factor 3	467
ENSG00000086061	DNAJA1	DnaJ (Hsp40) homolog, subfamily A, member 1	3301
ENSG00000160211	G6PD	glucose-6-phosphate dehydrogenase	2539
ENSG00000099860	GADD45B	growth arrest and DNA-damage-inducible, beta	4616
ENSG00000152137	HSPB8	heat shock 22kDa protein 8	26353
ENSG00000106070	GRB10	growth factor receptor-bound protein 10	2887
ENSG00000134363	FST	follistatin	10468
ENSG00000170689	HOXB9	homeobox B9	3219
ENSG00000146733	PSPH	phosphoserine phosphatase	5723
ENSG00000023909	GCLM	glutamate-cysteine ligase, modifier subunit	2730
ENSG00000116299	KIAA1324	KIAA1324	57535
ENSG00000131080	EDA2R	ectodysplasin A2 receptor	60401
ENSG00000152377	SPOCK1	sparc/osteonectin, cwcv and kazal-like domains proteoglycan (testican) 1	6695
ENSG00000160200	CBS	cystathionine-beta-synthase	875
ENSG00000130513	GDF15	growth differentiation factor 15	9518
ENSG00000048052	HDAC9	histone deacetylase 9	9734
ENSG00000123094	RASSF8	Ras association (RalGDS/AF-6) domain family (N-terminal) member 8	11228
ENSG00000148700	ADD3	adducin 3 (gamma)	120
ENSG00000170855	TRIAP1	TP53 regulated inhibitor of apoptosis 1	51499
ENSG00000136560	TANK	TRAF family member-associated NFKB activator	10010
ENSG00000010278	CD9	CD9 molecule	928
ENSG00000087088	BAX	BCL2-associated X protein	581
ENSG00000107796	ACTA2	actin, alpha 2, smooth muscle, aorta	59
ENSG00000080824	HSP90AA1	heat shock protein 90kDa alpha (cytosolic), class A member 1	3320
ENSG00000100889	PCK2	phosphoenolpyruvate carboxykinase 2 (mitochondrial)	5106
ENSG00000116285	ERRFI1	ERBB receptor feedback inhibitor 1	54206
ENSG00000170801	HTRA3	HtrA serine peptidase 3	94031
ENSG00000185591	SP1	Sp1 transcription factor	6667
ENSG00000198431	TXNRD1	thioredoxin reductase 1	7296
ENSG00000184995	IFNE	interferon, epsilon	338376
ENSG00000001084	GCLC	glutamate-cysteine ligase, catalytic subunit	2729

ENSG00000168243	GNG4	guanine nucleotide binding protein (G protein), gamma 4	2786
ENSG00000181222	POLR2A	polymerase (RNA) II (DNA directed) polypeptide A, 220kDa	5430
ENSG00000204388	HSPA1B	heat shock 70kDa protein 1B	3304
ENSG00000135047	CTSL1	cathepsin L1	1514
ENSG00000117318	ID3	inhibitor of DNA binding 3, dominant negative helix-loop-helix protein	3399
ENSG00000116717	GADD45A	growth arrest and DNA-damage-inducible, alpha	1647
ENSG00000168685	IL7R	interleukin 7 receptor	3575
ENSG00000181026	AEN	apoptosis enhancing nuclease	64782
ENSG00000132470	ITGB4	integrin, beta 4	3691
ENSG00000160570	DEDD2	death effector domain containing 2	162989
ENSG00000174307	PHLDA3	pleckstrin homology-like domain, family A, member 3	23612
ENSG00000136002	ARHGEF4	Rho guanine nucleotide exchange factor (GEF) 4	50649
**Biological process**	**Response to unfolded protein**	**GO:0006986**	
C=129;O=15;E=1.74;R=8.64;rawP=1.91e-10;adjP=3.87e-08			
ENSG00000204390	HSPA1L	heat shock 70kDa protein 1-like	3305
ENSG00000120694	HSPH1	heat shock 105kDa/110kDa protein 1	10808
ENSG00000162616	DNAJB4	DnaJ (Hsp40) homolog, subfamily B, member 4	11080
ENSG00000070669	ASNS	asparagine synthetase (glutamine-hydrolyzing)	440
ENSG00000105993	DNAJB6	DnaJ (Hsp40) homolog, subfamily B, member 6	10049
ENSG00000087074	PPP1R15A	protein phosphatase 1, regulatory subunit 15A	23645
ENSG00000087088	BAX	BCL2-associated X protein	581
ENSG00000162772	ATF3	activating transcription factor 3	467
ENSG00000080824	HSP90AA1	heat shock protein 90kDa alpha (cytosolic), class A member 1	3320
ENSG00000086061	DNAJA1	DnaJ (Hsp40) homolog, subfamily A, member 1	3301
ENSG00000132002	DNAJB1	DnaJ (Hsp40) homolog, subfamily B, member 1	3337
ENSG00000204388	HSPA1B	heat shock 70kDa protein 1B	3304
ENSG00000116761	CTH	cystathionase (cystathionine gamma-lyase)	1491
ENSG00000173110	HSPA6	heat shock 70kDa protein 6 (HSP70B')	3310
**Biological process**	**Regulation of programmed cell death**	**GO:0043067**	
C=1126;O=43;E=15.16;R=2.84;rawP=2.44e-10;adjP=4.39e-08			
ENSG00000023909	GCLM	glutamate-cysteine ligase, modifier subunit	2730
ENSG00000116299	KIAA1324	KIAA1324	57535
ENSG00000091592	NLRP1	NLR family, pyrin domain containing 1	22861
ENSG00000164938	TP53INP1	tumor protein p53 inducible nuclear protein 1	94241
ENSG00000124762	CDKN1A	cyclin-dependent kinase inhibitor 1A (p21, Cip1)	1026
ENSG00000100292	HMOX1	heme oxygenase (decycling) 1	3162
ENSG00000139289	PHLDA1	pleckstrin homology-like domain, family A, member 1	22822
ENSG00000100625	SIX4	SIX homeobox 4	51804
ENSG00000196072	BLOC1S2	biogenesis of lysosomal organelles complex-1, subunit 2	282991
ENSG00000085276	MECOM	MDS1 and EVI1 complex locus	2122
ENSG00000168209	DDIT4	DNA-damage-inducible transcript 4	54541

ENSG00000198121	LPAR1	lysophosphatidic acid receptor 1	1902
ENSG00000151929	BAG3	BCL2-associated athanogene 3	9531
ENSG00000135679	MDM2	Mdm2, p53 E3 ubiquitin protein ligase homolog (mouse)	4193
ENSG00000070669	ASNS	asparagine synthetase (glutamine-hydrolyzing)	440
ENSG00000105993	DNAJB6	DnaJ (Hsp40) homolog, subfamily B, member 6	10049
ENSG00000170855	TRIAP1	TP53 regulated inhibitor of apoptosis 1	51499
ENSG00000184254	ALDH1A3	aldehyde dehydrogenase 1 family, member A3	220
ENSG00000087088	BAX	BCL2-associated X protein	581
ENSG00000006327	TNFRSF12A	tumor necrosis factor receptor superfamily, member 12A	51330
ENSG00000181104	F2R	coagulation factor II (thrombin) receptor	2149
ENSG00000146674	IGFBP3	insulin-like growth factor binding protein 3	3486
ENSG00000173846	PLK3	polo-like kinase 3	1263
ENSG00000162734	PEA15	phosphoprotein enriched in astrocytes 15	8682
ENSG00000108179	PPIF	peptidylprolyl isomerase F	10105
ENSG00000142168	SOD1	superoxide dismutase 1, soluble	6647
ENSG00000185088	RPS27L	ribosomal protein S27-like	51065
ENSG00000132170	PPARG	peroxisome proliferator-activated receptor gamma	5468
ENSG00000001084	GCLC	glutamate-cysteine ligase, catalytic subunit	2729
ENSG00000159388	BTG2	BTG family, member 2	7832
ENSG00000115641	FHL2	four and a half LIM domains 2	2274
ENSG00000204388	HSPA1B	heat shock 70kDa protein 1B	3304
ENSG00000026103	FAS	Fas (TNF receptor superfamily, member 6)	355
ENSG00000121691	CAT	catalase	847
ENSG00000117318	ID3	inhibitor of DNA binding 3, dominant negative helix-loop-helix protein	3399
ENSG00000181026	AEN	apoptosis enhancing nuclease	64782
ENSG00000105327	BBC3	BCL2 binding component 3	27113
ENSG00000120885	CLU	clusterin	1191
ENSG00000174307	PHLDA3	pleckstrin homology-like domain, family A, member 3	23612
ENSG00000160570	DEDD2	death effector domain containing 2	162989
**Biological process**	**Regulation of cell death**	**GO:0010941**	
C=1153;O=43;E=15.52;R=2.77;rawP=5.12e-10;adjP=8.30e-08			
ENSG00000023909	GCLM	glutamate-cysteine ligase, modifier subunit	2730
ENSG00000116299	KIAA1324	KIAA1324	57535
ENSG00000091592	NLRP1	NLR family, pyrin domain containing 1	22861
ENSG00000164938	TP53INP1	tumor protein p53 inducible nuclear protein 1	94241
ENSG00000124762	CDKN1A	cyclin-dependent kinase inhibitor 1A (p21, Cip1)	1026
ENSG00000100292	HMOX1	heme oxygenase (decycling) 1	3162
ENSG00000139289	PHLDA1	pleckstrin homology-like domain, family A, member 1	22822
ENSG00000100625	SIX4	SIX homeobox 4	51804
ENSG00000196072	BLOC1S2	biogenesis of lysosomal organelles complex-1, subunit 2	282991
ENSG00000085276	MECOM	MDS1 and EVI1 complex locus	2122
ENSG00000168209	DDIT4	DNA-damage-inducible transcript 4	54541
ENSG00000198121	LPAR1	lysophosphatidic acid receptor 1	1902
ENSG00000151929	BAG3	BCL2-associated athanogene 3	9531

ENSG00000135679	MDM2	Mdm2, p53 E3 ubiquitin protein ligase homolog (mouse)	4193
ENSG00000070669	ASNS	asparagine synthetase (glutamine-hydrolyzing)	440
ENSG00000105993	DNAJB6	DnaJ (Hsp40) homolog, subfamily B, member 6	10049
ENSG00000170855	TRIAP1	TP53 regulated inhibitor of apoptosis 1	51499
ENSG00000184254	ALDH1A3	aldehyde dehydrogenase 1 family, member A3	220
ENSG00000087088	BAX	BCL2-associated X protein	581
ENSG00000006327	TNFRSF12A	tumor necrosis factor receptor superfamily, member 12A	51330
ENSG00000181104	F2R	coagulation factor II (thrombin) receptor	2149
ENSG00000146674	IGFBP3	insulin-like growth factor binding protein 3	3486
ENSG00000173846	PLK3	polo-like kinase 3	1263
ENSG00000162734	PEA15	phosphoprotein enriched in astrocytes 15	8682
ENSG00000108179	PPIF	peptidylprolyl isomerase F	10105
ENSG00000142168	SOD1	superoxide dismutase 1, soluble	6647
ENSG00000185088	RPS27L	ribosomal protein S27-like	51065
ENSG00000132170	PPARG	peroxisome proliferator-activated receptor gamma	5468
ENSG00000001084	GCLC	glutamate-cysteine ligase, catalytic subunit	2729
ENSG00000159388	BTG2	BTG family, member 2	7832
ENSG00000115641	FHL2	four and a half LIM domains 2	2274
ENSG00000204388	HSPA1B	heat shock 70kDa protein 1B	3304
ENSG00000026103	FAS	Fas (TNF receptor superfamily, member 6)	355
ENSG00000121691	CAT	catalase	847
ENSG00000117318	ID3	inhibitor of DNA binding 3, dominant negative helix-loop-helix protein	3399
ENSG00000181026	AEN	apoptosis enhancing nuclease	64782
ENSG00000105327	BBC3	BCL2 binding component 3	27113
ENSG00000120885	CLU	clusterin	1191
ENSG00000174307	PHLDA3	pleckstrin homology-like domain, family A, member 3	23612
ENSG00000160570	DEDD2	death effector domain containing 2	162989
**Molecular function**	**Protein binding**	**GO:0005515**	
C=7048;O=135;E=93.01;R=1.45;rawP=2.68e-10;adjP=6.97e-08			
ENSG00000162616	DNAJB4	DnaJ (Hsp40) homolog, subfamily B, member 4	11080
ENSG00000163257	DCAF16	DDB1 and CUL4 associated factor 16	54876
ENSG00000221926	TRIM16	tripartite motif containing 16	10626
ENSG00000091592	NLRP1	NLR family, pyrin domain containing 1	22861
ENSG00000146072	TNFRSF21	tumor necrosis factor receptor superfamily, member 21	27242
ENSG00000124762	CDKN1A	cyclin-dependent kinase inhibitor 1A (p21, Cip1)	1026
ENSG00000104368	PLAT	plasminogen activator, tissue	5327
ENSG00000140941	MAP1LC3B	microtubule-associated protein 1 light chain 3 beta	81631
ENSG00000171517	LPAR3	lysophosphatidic acid receptor 3	23566
ENSG00000169136	ATF5	activating transcription factor 5	22809
ENSG00000198121	LPAR1	lysophosphatidic acid receptor 1	1902
ENSG00000151929	BAG3	BCL2-associated athanogene 3	9531
ENSG00000154175	ABI3BP	ABI family, member 3 (NESH) binding protein	25890
ENSG00000128016	ZFP36	zinc finger protein 36, C3H type, homolog (mouse)	7538
ENSG00000184254	ALDH1A3	aldehyde dehydrogenase 1 family, member A3	220

ENSG00000239713	APOBEC3G	apolipoprotein B mRNA editing enzyme, catalytic polypeptide-like 3G	60489
ENSG00000173846	PLK3	polo-like kinase 3	1263
ENSG00000142168	SOD1	superoxide dismutase 1, soluble	6647
ENSG00000185088	RPS27L	ribosomal protein S27-like	51065
ENSG00000132170	PPARG	peroxisome proliferator-activated receptor gamma	5468
ENSG00000197903	HIST1H2BK	histone cluster 1, H2bk	85236
ENSG00000079308	TNS1	tensin 1	7145
ENSG00000138413	IDH1	isocitrate dehydrogenase 1 (NADP+), soluble	3417
ENSG00000160255	ITGB2	integrin, beta 2 (complement component 3 receptor 3 and 4 subunit)	3689
ENSG00000119630	PGF	placental growth factor	5228
ENSG00000186480	INSIG1	insulin induced gene 1	3638
ENSG00000142627	EPHA2	EPH receptor A2	1969
ENSG00000088356	PDRG1	p53 and DNA-damage regulated 1	81572
ENSG00000115919	KYNU	kynureninase	8942
ENSG00000105327	BBC3	BCL2 binding component 3	27113
ENSG00000075223	SEMA3C	sema domain, immunoglobulin domain (Ig), short basic domain, secreted, (semaphorin) 3C	10512
ENSG00000120885	CLU	clusterin	1191
ENSG00000120694	HSPH1	heat shock 105kDa/110kDa protein 1	10808
ENSG00000156273	BACH1	BTB and CNC homology 1, basic leucine zipper transcription factor 1	571
ENSG00000120738	EGR1	early growth response 1	1958
ENSG00000074590	NUAK1	NUAK family, SNF1-like kinase, 1	9891
ENSG00000164938	TP53INP1	tumor protein p53 inducible nuclear protein 1	94241
ENSG00000197019	SERTAD1	SERTA domain containing 1	29950
ENSG00000102048	ASB9	ankyrin repeat and SOCS box containing 9	140462
ENSG00000132002	DNAJB1	DnaJ (Hsp40) homolog, subfamily B, member 1	3337
ENSG00000168209	DDIT4	DNA-damage-inducible transcript 4	54541
ENSG00000085276	MECOM	MDS1 and EVI1 complex locus	2122
ENSG00000161011	SQSTM1	sequestosome 1	8878
ENSG00000072310	SREBF1	sterol regulatory element binding transcription factor 1	6720
ENSG00000006327	TNFRSF12A	tumor necrosis factor receptor superfamily, member 12A	51330
ENSG00000121931	LRIF1	ligand dependent nuclear receptor interacting factor 1	55791
ENSG00000162734	PEA15	phosphoprotein enriched in astrocytes 15	8682
ENSG00000134574	DDB2	damage-specific DNA binding protein 2, 48kDa	1643
ENSG00000173614	NMNAT1	nicotinamide nucleotide adenylyltransferase 1	64802
ENSG00000115641	FHL2	four and a half LIM domains 2	2274
ENSG00000179583	CIITA	class II, major histocompatibility complex, transactivator	4261
ENSG00000026103	FAS	Fas (TNF receptor superfamily, member 6)	355
ENSG00000095383	TBC1D2	TBC1 domain family, member 2	55357
ENSG00000121691	CAT	catalase	847
ENSG00000087074	PPP1R15A	protein phosphatase 1, regulatory subunit 15A	23645
ENSG00000153879	CEBPG	CCAAT/enhancer binding protein (C/EBP), gamma	1054
ENSG00000255150	EID3	EP300 interacting inhibitor of differentiation 3	493861
ENSG00000101849	TBL1X	transducin (beta)-like 1X-linked	6907

ENSG00000105939	ZC3HAV1	zinc finger CCCH-type, antiviral 1	56829
ENSG00000095752	IL11	interleukin 11	3589
ENSG00000124496	TRERF1	transcriptional regulating factor 1	55809
ENSG00000170485	NPAS2	neuronal PAS domain protein 2	4862
ENSG00000162733	DDR2	discoidin domain receptor tyrosine kinase 2	4921
ENSG00000167996	FTH1	ferritin, heavy polypeptide 1	2495
ENSG00000139289	PHLDA1	pleckstrin homology-like domain, family A, member 1	22822
ENSG00000100292	HMOX1	heme oxygenase (decycling) 1	3162
ENSG00000125657	TNFSF9	tumor necrosis factor (ligand) superfamily, member 9	8744
ENSG00000059728	MXD1	MAX dimerization protein 1	4084
ENSG00000196072	BLOC1S2	biogenesis of lysosomal organelles complex-1, subunit 2	282991
ENSG00000070669	ASNS	asparagine synthetase (glutamine-hydrolyzing)	440
ENSG00000135679	MDM2	Mdm2, p53 E3 ubiquitin protein ligase homolog (mouse)	4193
ENSG00000105993	DNAJB6	DnaJ (Hsp40) homolog, subfamily B, member 6	10049
ENSG00000162892	IL24	interleukin 24	11009
ENSG00000181104	F2R	coagulation factor II (thrombin) receptor	2149
ENSG00000063660	GPC1	glypican 1	2817
ENSG00000158373	HIST1H2BD	histone cluster 1, H2bd	3017
ENSG00000146674	IGFBP3	insulin-like growth factor binding protein 3	3486
ENSG00000116761	CTH	cystathionase (cystathionine gamma-lyase)	1491
ENSG00000106105	GARS	glycyl-tRNA synthetase	2617
ENSG00000159388	BTG2	BTG family, member 2	7832
ENSG00000110172	CHORDC1	cysteine and histidine-rich domain (CHORD) containing 1	26973
ENSG00000162772	ATF3	activating transcription factor 3	467
ENSG00000086061	DNAJA1	DnaJ (Hsp40) homolog, subfamily A, member 1	3301
ENSG00000171617	ENC1	ectodermal-neural cortex 1 (with BTB-like domain)	8507
ENSG00000162545	CAMK2N1	calcium/calmodulin-dependent protein kinase II inhibitor 1	55450
ENSG00000160211	G6PD	glucose-6-phosphate dehydrogenase	2539
ENSG00000152137	HSPB8	heat shock 22kDa protein 8	26353
ENSG00000106070	GRB10	growth factor receptor-bound protein 10	2887
ENSG00000134363	FST	follistatin	10468
ENSG00000170689	HOXB9	homeobox B9	3219
ENSG00000146733	PSPH	phosphoserine phosphatase	5723
ENSG00000023909	GCLM	glutamate-cysteine ligase, modifier subunit	2730
ENSG00000131080	EDA2R	ectodysplasin A2 receptor	60401
ENSG00000160200	CBS	cystathionine-beta-synthase	875
ENSG00000109220	CHIC2	cysteine-rich hydrophobic domain 2	26511
ENSG00000026950	BTN3A1	butyrophilin, subfamily 3, member A1	11119
ENSG00000130513	GDF15	growth differentiation factor 15	9518
ENSG00000048052	HDAC9	histone deacetylase 9	9734
ENSG00000148700	ADD3	adducin 3 (gamma)	120
ENSG00000170855	TRIAP1	TP53 regulated inhibitor of apoptosis 1	51499
ENSG00000136560	TANK	TRAF family member-associated NFKB activator	10010
ENSG00000161513	FDXR	ferredoxin reductase	2232

ENSG00000010278	CD9	CD9 molecule	928
ENSG00000087088	BAX	BCL2-associated X protein	581
ENSG00000080824	HSP90AA1	heat shock protein 90kDa alpha (cytosolic), class A member 1	3320
ENSG00000100889	PCK2	phosphoenolpyruvate carboxykinase 2 (mitochondrial)	5106
ENSG00000116285	ERRFI1	ERBB receptor feedback inhibitor 1	54206
ENSG00000170801	HTRA3	HtrA serine peptidase 3	94031
ENSG00000140961	OSGIN1	oxidative stress induced growth inhibitor 1	29948
ENSG00000185591	SP1	Sp1 transcription factor	6667
ENSG00000240694	PNMA2	paraneoplastic Ma antigen 2	10687
ENSG00000184995	IFNE	interferon, epsilon	338376
ENSG00000143217	PVRL4	poliovirus receptor-related 4	81607
ENSG00000001084	GCLC	glutamate-cysteine ligase, catalytic subunit	2729
ENSG00000181222	POLR2A	polymerase (RNA) II (DNA directed) polypeptide A, 220kDa	5430
ENSG00000204388	HSPA1B	heat shock 70kDa protein 1B	3304
ENSG00000135047	CTSL1	cathepsin L1	1514
ENSG00000117318	ID3	inhibitor of DNA binding 3, dominant negative helix-loop-helix protein	3399
ENSG00000116717	GADD45A	growth arrest and DNA-damage-inducible, alpha	1647
ENSG00000168685	IL7R	interleukin 7 receptor	3575
ENSG00000166897	ELFN2	extracellular leucine-rich repeat and fibronectin type III domain containing 2	114794
ENSG00000132470	ITGB4	integrin, beta 4	3691
ENSG00000160570	DEDD2	death effector domain containing 2	162989
ENSG00000136002	ARHGEF4	Rho guanine nucleotide exchange factor (GEF) 4	50649
**Molecular function**	**Protein dimerization activity**	**GO:0046983**	
C=926;O=31;E=12.22;R=2.54;rawP=1.41e-06;adjP=0.0001			
ENSG00000023909	GCLM	glutamate-cysteine ligase, modifier subunit	2730
ENSG00000156273	BACH1	BTB and CNC homology 1, basic leucine zipper transcription factor 1	571
ENSG00000106105	GARS	glycyl-tRNA synthetase	2617
ENSG00000142168	SOD1	superoxide dismutase 1, soluble	6647
ENSG00000170485	NPAS2	neuronal PAS domain protein 2	4862
ENSG00000001084	GCLC	glutamate-cysteine ligase, catalytic subunit	2729
ENSG00000197903	HIST1H2BK	histone cluster 1, H2bk	85236
ENSG00000160200	CBS	cystathionine-beta-synthase	875
ENSG00000162772	ATF3	activating transcription factor 3	467
ENSG00000100292	HMOX1	heme oxygenase (decycling) 1	3162
ENSG00000138413	IDH1	isocitrate dehydrogenase 1 (NADP+), soluble	3417
ENSG00000169136	ATF5	activating transcription factor 5	22809
ENSG00000059728	MXD1	MAX dimerization protein 1	4084
ENSG00000085276	MECOM	MDS1 and EVI1 complex locus	2122
ENSG00000119630	PGF	placental growth factor	5228
ENSG00000121691	CAT	catalase	847
ENSG00000117318	ID3	inhibitor of DNA binding 3, dominant negative helix-loop-helix protein	3399

ENSG00000070669	ASNS	asparagine synthetase (glutamine-hydrolyzing)	440
ENSG00000160211	G6PD	glucose-6-phosphate dehydrogenase	2539
ENSG00000115919	KYNU	kynureninase	8942
ENSG00000184254	ALDH1A3	aldehyde dehydrogenase 1 family, member A3	220
ENSG00000087088	BAX	BCL2-associated X protein	581
ENSG00000072310	SREBF1	sterol regulatory element binding transcription factor 1	6720
ENSG00000153879	CEBPG	CCAAT/enhancer binding protein (C/EBP), gamma	1054
ENSG00000080824	HSP90AA1	heat shock protein 90kDa alpha (cytosolic), class A member 1	3320
ENSG00000158373	HIST1H2BD	histone cluster 1, H2bd	3017
ENSG00000239713	APOBEC3G	apolipoprotein B mRNA editing enzyme, catalytic polypeptide-like 3G	60489
ENSG00000146733	PSPH	phosphoserine phosphatase	5723
ENSG00000185591	SP1	Sp1 transcription factor	6667
**Molecular function**	**Cofactor binding**	**GO:0048037**	
C=251;O=15;E=3.31;R=4.53;rawP=1.19e-06;adjP=0.0001			
ENSG00000198431	TXNRD1	thioredoxin reductase 1	7296
ENSG00000121691	CAT	catalase	847
ENSG00000166123	GPT2	glutamic pyruvate transaminase (alanine aminotransferase) 2	84706
ENSG00000070669	ASNS	asparagine synthetase (glutamine-hydrolyzing)	440
ENSG00000135069	PSAT1	phosphoserine aminotransferase 1	29968
ENSG00000001084	GCLC	glutamate-cysteine ligase, catalytic subunit	2729
ENSG00000160211	G6PD	glucose-6-phosphate dehydrogenase	2539
ENSG00000115919	KYNU	kynureninase	8942
ENSG00000184254	ALDH1A3	aldehyde dehydrogenase 1 family, member A3	220
ENSG00000109814	UGDH	UDP-glucose 6-dehydrogenase	7358
ENSG00000023330	ALAS1	aminolevulinate, delta-, synthase 1	211
ENSG00000160200	CBS	cystathionine-beta-synthase	875
ENSG00000138413	IDH1	isocitrate dehydrogenase 1 (NADP+), soluble	3417
ENSG00000116761	CTH	cystathionase (cystathionine gamma-lyase)	1491
**Molecular function**	**Binding**	**GO:0005488**	
C=11168;O=170;E=147.37;R=1.15;rawP=7.09e-06;adjP=0.0005			
ENSG00000162616	DNAJB4	DnaJ (Hsp40) homolog, subfamily B, member 4	11080
ENSG00000163257	DCAF16	DDB1 and CUL4 associated factor 16	54876
ENSG00000158286	RNF207	ring finger protein 207	388591
ENSG00000221926	TRIM16	tripartite motif containing 16	10626
ENSG00000091592	NLRP1	NLR family, pyrin domain containing 1	22861
ENSG00000146072	TNFRSF21	tumor necrosis factor receptor superfamily, member 21	27242
ENSG00000109814	UGDH	UDP-glucose 6-dehydrogenase	7358
ENSG00000128965	CHAC1	ChaC, cation transport regulator homolog 1 (E. coli)	79094
ENSG00000124762	CDKN1A	cyclin-dependent kinase inhibitor 1A (p21, Cip1)	1026
ENSG00000128510	CPA4	carboxypeptidase A4	51200
ENSG00000140941	MAP1LC3B	microtubule-associated protein 1 light chain 3 beta	81631
ENSG00000104368	PLAT	plasminogen activator, tissue	5327
ENSG00000141569	TRIM65	tripartite motif containing 65	201292

ENSG00000171517	LPAR3	lysophosphatidic acid receptor 3	23566
ENSG00000169136	ATF5	activating transcription factor 5	22809
ENSG00000112319	EYA4	eyes absent homolog 4 (Drosophila)	2070
ENSG00000099994	SUSD2	sushi domain containing 2	56241
ENSG00000151929	BAG3	BCL2-associated athanogene 3	9531
ENSG00000198121	LPAR1	lysophosphatidic acid receptor 1	1902
ENSG00000060140	STYK1	serine/threonine/tyrosine kinase 1	55359
ENSG00000154175	ABI3BP	ABI family, member 3 (NESH) binding protein	25890
ENSG00000128016	ZFP36	zinc finger protein 36, C3H type, homolog (mouse)	7538
ENSG00000184254	ALDH1A3	aldehyde dehydrogenase 1 family, member A3	220
ENSG00000239713	APOBEC3G	apolipoprotein B mRNA editing enzyme, catalytic polypeptide-like 3G	60489
ENSG00000173846	PLK3	polo-like kinase 3	1263
ENSG00000142168	SOD1	superoxide dismutase 1, soluble	6647
ENSG00000185088	RPS27L	ribosomal protein S27-like	51065
ENSG00000132170	PPARG	peroxisome proliferator-activated receptor gamma	5468
ENSG00000197903	HIST1H2BK	histone cluster 1, H2bk	85236
ENSG00000079308	TNS1	tensin 1	7145
ENSG00000138413	IDH1	isocitrate dehydrogenase 1 (NADP+), soluble	3417
ENSG00000173110	HSPA6	heat shock 70kDa protein 6 (HSP70B')	3310
ENSG00000147130	ZMYM3	zinc finger, MYM-type 3	9203
ENSG00000204390	HSPA1L	heat shock 70kDa protein 1-like	3305
ENSG00000119630	PGF	placental growth factor	5228
ENSG00000160255	ITGB2	integrin, beta 2 (complement component 3 receptor 3 and 4 subunit)	3689
ENSG00000142627	EPHA2	EPH receptor A2	1969
ENSG00000186480	INSIG1	insulin induced gene 1	3638
ENSG00000088356	PDRG1	p53 and DNA-damage regulated 1	81572
ENSG00000115919	KYNU	kynureninase	8942
ENSG00000023330	ALAS1	aminolevulinate, delta-, synthase 1	211
ENSG00000105327	BBC3	BCL2 binding component 3	27113
ENSG00000075223	SEMA3C	sema domain, immunoglobulin domain (Ig), short basic domain, secreted, (semaphorin) 3C	10512
ENSG00000120885	CLU	clusterin	1191
ENSG00000166123	GPT2	glutamic pyruvate transaminase (alanine aminotransferase) 2	84706
ENSG00000120694	HSPH1	heat shock 105kDa/110kDa protein 1	10808
ENSG00000156273	BACH1	BTB and CNC homology 1, basic leucine zipper transcription factor 1	571
ENSG00000120738	EGR1	early growth response 1	1958
ENSG00000074590	NUAK1	NUAK family, SNF1-like kinase, 1	9891
ENSG00000164938	TP53INP1	tumor protein p53 inducible nuclear protein 1	94241
ENSG00000197019	SERTAD1	SERTA domain containing 1	29950
ENSG00000102048	ASB9	ankyrin repeat and SOCS box containing 9	140462
ENSG00000132002	DNAJB1	DnaJ (Hsp40) homolog, subfamily B, member 1	3337
ENSG00000168209	DDIT4	DNA-damage-inducible transcript 4	54541
ENSG00000085276	MECOM	MDS1 and EVI1 complex locus	2122
ENSG00000148288	GBGT1	globoside alpha-1,3-N-acetylgalactosaminyltransferase 1	26301

ENSG00000161011	SQSTM1	sequestosome 1	8878
ENSG00000112297	AIM1	absent in melanoma 1	202
ENSG00000072310	SREBF1	sterol regulatory element binding transcription factor 1	6720
ENSG00000006327	TNFRSF12A	tumor necrosis factor receptor superfamily, member 12A	51330
ENSG00000146453	PNLDC1	poly(A)-specific ribonuclease (PARN)-like domain containing 1	154197
ENSG00000121931	LRIF1	ligand dependent nuclear receptor interacting factor 1	55791
ENSG00000003137	CYP26B1	cytochrome P450, family 26, subfamily B, polypeptide 1	56603
ENSG00000162734	PEA15	phosphoprotein enriched in astrocytes 15	8682
ENSG00000134574	DDB2	damage-specific DNA binding protein 2, 48kDa	1643
ENSG00000173614	NMNAT1	nicotinamide nucleotide adenylyltransferase 1	64802
ENSG00000115641	FHL2	four and a half LIM domains 2	2274
ENSG00000154556	SORBS2	sorbin and SH3 domain containing 2	8470
ENSG00000179583	CIITA	class II, major histocompatibility complex, transactivator	4261
ENSG00000095383	TBC1D2	TBC1 domain family, member 2	55357
ENSG00000026103	FAS	Fas (TNF receptor superfamily, member 6)	355
ENSG00000121691	CAT	catalase	847
ENSG00000065911	MTHFD2	methylenetetrahydrofolate dehydrogenase (NADP+ dependent) 2, methenyltetrahydrofolate cyclohydrolase	10797
ENSG00000087074	PPP1R15A	protein phosphatase 1, regulatory subunit 15A	23645
ENSG00000153879	CEBPG	CCAAT/enhancer binding protein (C/EBP), gamma	1054
ENSG00000255150	EID3	EP300 interacting inhibitor of differentiation 3	493861
ENSG00000101849	TBL1X	transducin (beta)-like 1X-linked	6907
ENSG00000105939	ZC3HAV1	zinc finger CCCH-type, antiviral 1	56829
ENSG00000095752	IL11	interleukin 11	3589
ENSG00000124496	TRERF1	transcriptional regulating factor 1	55809
ENSG00000170485	NPAS2	neuronal PAS domain protein 2	4862
ENSG00000162733	DDR2	discoidin domain receptor tyrosine kinase 2	4921
ENSG00000139289	PHLDA1	pleckstrin homology-like domain, family A, member 1	22822
ENSG00000100292	HMOX1	heme oxygenase (decycling) 1	3162
ENSG00000125657	TNFSF9	tumor necrosis factor (ligand) superfamily, member 9	8744
ENSG00000059728	MXD1	MAX dimerization protein 1	4084
ENSG00000196072	BLOC1S2	biogenesis of lysosomal organelles complex-1, subunit 2	282991
ENSG00000178381	ZFAND2A	zinc finger, AN1-type domain 2A	90637
ENSG00000070669	ASNS	asparagine synthetase (glutamine-hydrolyzing)	440
ENSG00000135679	MDM2	Mdm2, p53 E3 ubiquitin protein ligase homolog (mouse)	4193
ENSG00000105993	DNAJB6	DnaJ (Hsp40) homolog, subfamily B, member 6	10049
ENSG00000162892	IL24	interleukin 24	11009
ENSG00000181104	F2R	coagulation factor II (thrombin) receptor	2149
ENSG00000063660	GPC1	glypican 1	2817
ENSG00000158373	HIST1H2BD	histone cluster 1, H2bd	3017
ENSG00000146674	IGFBP3	insulin-like growth factor binding protein 3	3486
ENSG00000116761	CTH	cystathionase (cystathionine gamma-lyase)	1491
ENSG00000108179	PPIF	peptidylprolyl isomerase F	10105
ENSG00000106105	GARS	glycyl-tRNA synthetase	2617
ENSG00000159388	BTG2	BTG family, member 2	7832

ENSG00000110172	CHORDC1	cysteine and histidine-rich domain (CHORD) containing 1	26973
ENSG00000162772	ATF3	activating transcription factor 3	467
ENSG00000086061	DNAJA1	DnaJ (Hsp40) homolog, subfamily A, member 1	3301
ENSG00000171617	ENC1	ectodermal-neural cortex 1 (with BTB-like domain)	8507
ENSG00000162545	CAMK2N1	calcium/calmodulin-dependent protein kinase II inhibitor 1	55450
ENSG00000160211	G6PD	glucose-6-phosphate dehydrogenase	2539
ENSG00000152137	HSPB8	heat shock 22kDa protein 8	26353
ENSG00000106070	GRB10	growth factor receptor-bound protein 10	2887
ENSG00000129282	MRM1	mitochondrial rRNA methyltransferase 1 homolog (S. cerevisiae)	79922
ENSG00000134363	FST	follistatin	10468
ENSG00000170689	HOXB9	homeobox B9	3219
ENSG00000146733	PSPH	phosphoserine phosphatase	5723
ENSG00000023909	GCLM	glutamate-cysteine ligase, modifier subunit	2730
ENSG00000131080	EDA2R	ectodysplasin A2 receptor	60401
ENSG00000152377	SPOCK1	sparc/osteonectin, cwcv and kazal-like domains proteoglycan (testican) 1	6695
ENSG00000026950	BTN3A1	butyrophilin, subfamily 3, member A1	11119
ENSG00000109220	CHIC2	cysteine-rich hydrophobic domain 2	26511
ENSG00000160200	CBS	cystathionine-beta-synthase	875
ENSG00000130513	GDF15	growth differentiation factor 15	9518
ENSG00000100625	SIX4	SIX homeobox 4	51804
ENSG00000048052	HDAC9	histone deacetylase 9	9734
ENSG00000148700	ADD3	adducin 3 (gamma)	120
ENSG00000161513	FDXR	ferredoxin reductase	2232
ENSG00000136560	TANK	TRAF family member-associated NFKB activator	10010
ENSG00000170855	TRIAP1	TP53 regulated inhibitor of apoptosis 1	51499
ENSG00000087088	BAX	BCL2-associated X protein	581
ENSG00000010278	CD9	CD9 molecule	928
ENSG00000107796	ACTA2	actin, alpha 2, smooth muscle, aorta	59
ENSG00000080824	HSP90AA1	heat shock protein 90kDa alpha (cytosolic), class A member 1	3320
ENSG00000170801	HTRA3	HtrA serine peptidase 3	94031
ENSG00000116285	ERRFI1	ERBB receptor feedback inhibitor 1	54206
ENSG00000100889	PCK2	phosphoenolpyruvate carboxykinase 2 (mitochondrial)	5106
ENSG00000140961	OSGIN1	oxidative stress induced growth inhibitor 1	29948
ENSG00000240694	PNMA2	paraneoplastic Ma antigen 2	10687
ENSG00000185591	SP1	Sp1 transcription factor	6667
ENSG00000143217	PVRL4	poliovirus receptor-related 4	81607
ENSG00000184995	IFNE	interferon, epsilon	338376
ENSG00000198431	TXNRD1	thioredoxin reductase 1	7296
ENSG00000135069	PSAT1	phosphoserine aminotransferase 1	29968
ENSG00000156218	ADAMTSL3	ADAMTS-like 3	57188
ENSG00000001084	GCLC	glutamate-cysteine ligase, catalytic subunit	2729
ENSG00000160161	CILP2	cartilage intermediate layer protein 2	148113

ENSG00000181222	POLR2A	polymerase (RNA) II (DNA directed) polypeptide A, 220kDa	5430
ENSG00000204388	HSPA1B	heat shock 70kDa protein 1B	3304
ENSG00000135047	CTSL1	cathepsin L1	1514
ENSG00000117318	ID3	inhibitor of DNA binding 3, dominant negative helix-loop-helix protein	3399
ENSG00000116717	GADD45A	growth arrest and DNA-damage-inducible, alpha	1647
ENSG00000168685	IL7R	interleukin 7 receptor	3575
ENSG00000166897	ELFN2	extracellular leucine-rich repeat and fibronectin type III domain containing 2	114794
ENSG00000181026	AEN	apoptosis enhancing nuclease	64782
ENSG00000221963	APOL6	apolipoprotein L, 6	80830
ENSG00000118496	FBXO30	F-box protein 30	84085
ENSG00000132470	ITGB4	integrin, beta 4	3691
ENSG00000174307	PHLDA3	pleckstrin homology-like domain, family A, member 3	23612
ENSG00000160570	DEDD2	death effector domain containing 2	162989
ENSG00000136002	ARHGEF4	Rho guanine nucleotide exchange factor (GEF) 4	50649
**Molecular function**	**Identical protein binding**	**GO:0042802**	
C=836;O=27;E=11.03;R=2.45;rawP=1.41e-05;adjP=0.0007			
ENSG00000142168	SOD1	superoxide dismutase 1, soluble	6647
ENSG00000115641	FHL2	four and a half LIM domains 2	2274
ENSG00000160200	CBS	cystathionine-beta-synthase	875
ENSG00000162772	ATF3	activating transcription factor 3	467
ENSG00000100292	HMOX1	heme oxygenase (decycling) 1	3162
ENSG00000138413	IDH1	isocitrate dehydrogenase 1 (NADP+), soluble	3417
ENSG00000026103	FAS	Fas (TNF receptor superfamily, member 6)	355
ENSG00000085276	MECOM	MDS1 and EVI1 complex locus	2122
ENSG00000119630	PGF	placental growth factor	5228
ENSG00000121691	CAT	catalase	847
ENSG00000135679	MDM2	Mdm2, p53 E3 ubiquitin protein ligase homolog (mouse)	4193
ENSG00000070669	ASNS	asparagine synthetase (glutamine-hydrolyzing)	440
ENSG00000161011	SQSTM1	sequestosome 1	8878
ENSG00000160211	G6PD	glucose-6-phosphate dehydrogenase	2539
ENSG00000115919	KYNU	kynureninase	8942
ENSG00000184254	ALDH1A3	aldehyde dehydrogenase 1 family, member A3	220
ENSG00000087088	BAX	BCL2-associated X protein	581
ENSG00000152137	HSPB8	heat shock 22kDa protein 8	26353
ENSG00000153879	CEBPG	CCAAT/enhancer binding protein (C/EBP), gamma	1054
ENSG00000255150	EID3	EP300 interacting inhibitor of differentiation 3	493861
ENSG00000080824	HSP90AA1	heat shock protein 90kDa alpha (cytosolic), class A member 1	3320
ENSG00000239713	APOBEC3G	apolipoprotein B mRNA editing enzyme, catalytic polypeptide-like 3G	60489
ENSG00000146733	PSPH	phosphoserine phosphatase	5723
ENSG00000185591	SP1	Sp1 transcription factor	6667
**Molecular function**	**Vitamin B6 binding**	**GO:0070279**	

C=54;O=6;E=0.71;R=8.42;rawP=7.44e-05;adjP=0.0028			
ENSG00000160200	CBS	cystathionine-beta-synthase	875
ENSG00000166123	GPT2	glutamic pyruvate transaminase (alanine aminotransferase) 2	84706
ENSG00000135069	PSAT1	phosphoserine aminotransferase 1	29968
ENSG00000115919	KYNU	kynureninase	8942
ENSG00000116761	CTH	cystathionase (cystathionine gamma-lyase)	1491
ENSG00000023330	ALAS1	aminolevulinate, delta-, synthase 1	211
**Molecular function**	**Pyridoxal phosphate binding**	**GO:0030170**	
C=54;O=6;E=0.71;R=8.42;rawP=7.44e-05;adjP=0.0028			
ENSG00000160200	CBS	cystathionine-beta-synthase	875
ENSG00000166123	GPT2	glutamic pyruvate transaminase (alanine aminotransferase) 2	84706
ENSG00000135069	PSAT1	phosphoserine aminotransferase 1	29968
ENSG00000115919	KYNU	kynureninase	8942
ENSG00000116761	CTH	cystathionase (cystathionine gamma-lyase)	1491
ENSG00000023330	ALAS1	aminolevulinate, delta-, synthase 1	211
**Molecular function**	**Glutamate-cysteine ligase activity**	**GO:0004357**	
C=2;O=2;E=0.03;R=75.78;rawP=0.0002;adjP=0.0065			
ENSG00000023909	GCLM	glutamate-cysteine ligase, modifier subunit	2730
ENSG00000001084	GCLC	glutamate-cysteine ligase, catalytic subunit	2729
**Molecular function**	**Protein homodimerization activity**	**GO:0042803**	
C=551;O=18;E=7.27;R=2.48;rawP=0.0004;adjP=0.0116			
ENSG00000142168	SOD1	superoxide dismutase 1, soluble	6647
ENSG00000160200	CBS	cystathionine-beta-synthase	875
ENSG00000100292	HMOX1	heme oxygenase (decycling) 1	3162
ENSG00000138413	IDH1	isocitrate dehydrogenase 1 (NADP+), soluble	3417
ENSG00000085276	MECOM	MDS1 and EVI1 complex locus	2122
ENSG00000119630	PGF	placental growth factor	5228
ENSG00000121691	CAT	catalase	847
ENSG00000070669	ASNS	asparagine synthetase (glutamine-hydrolyzing)	440
ENSG00000115919	KYNU	kynureninase	8942
ENSG00000160211	G6PD	glucose-6-phosphate dehydrogenase	2539
ENSG00000184254	ALDH1A3	aldehyde dehydrogenase 1 family, member A3	220
ENSG00000153879	CEBPG	CCAAT/enhancer binding protein (C/EBP), gamma	1054
ENSG00000087088	BAX	BCL2-associated X protein	581
ENSG00000080824	HSP90AA1	heat shock protein 90kDa alpha (cytosolic), class A member 1	3320
ENSG00000239713	APOBEC3G	apolipoprotein B mRNA editing enzyme, catalytic polypeptide-like 3G	60489
ENSG00000146733	PSPH	phosphoserine phosphatase	5723
ENSG00000185591	SP1	Sp1 transcription factor	6667

**Molecular function**	**Lysophosphatidic acid receptor activity**	**GO:0070915**	
C=3;O=2;E=0.04;R=50.52;rawP=0.0005;adjP=0.0118			
ENSG00000198121	LPAR1	lysophosphatidic acid receptor 1	1902
ENSG00000171517	LPAR3	lysophosphatidic acid receptor 3	23566
**Cellular component**	**lysophosphatidic acid receptor activity**	**GO:0070915**	
C=2;O=2;E=0.02;R=80.98;rawP=0.0002;adjP=0.0116			
ENSG00000167996	FTH1	ferritin, heavy polypeptide 1	2495
ENSG00000087086	FTL	ferritin, light polypeptide	2512
**Cellular component**	**Intracellular ferritin complex**	**GO:0008043**	
C=2;O=2;E=0.02;R=80.98;rawP=0.0002;adjP=0.0116			
ENSG00000167996	FTH1	ferritin, heavy polypeptide 1	2495
ENSG00000087086	FTL	ferritin, light polypeptide	2512
**Cellular component**	**Glutamate-cysteine ligase complex**	**GO:0017109**	
C=2;O=2;E=0.02;R=80.98;rawP=0.0002;adjP=0.0116			
ENSG00000023909	GCLM	glutamate-cysteine ligase, modifier subunit	2730
ENSG00000001084	GCLC	glutamate-cysteine ligase, catalytic subunit	2729
**Cellular component**	**Cytosol**	**GO:0005829**	
C=2304;O=46;E=28.45;R=1.62;rawP=0.0005;adjP=0.0217			
ENSG00000023909	GCLM	glutamate-cysteine ligase, modifier subunit	2730
ENSG00000156273	BACH1	BTB and CNC homology 1, basic leucine zipper transcription factor 1	571
ENSG00000091592	NLRP1	NLR family, pyrin domain containing 1	22861
ENSG00000170485	NPAS2	neuronal PAS domain protein 2	4862
ENSG00000164938	TP53INP1	tumor protein p53 inducible nuclear protein 1	94241
ENSG00000109814	UGDH	UDP-glucose 6-dehydrogenase	7358
ENSG00000160200	CBS	cystathionine-beta-synthase	875
ENSG00000124762	CDKN1A	cyclin-dependent kinase inhibitor 1A (p21, Cip1)	1026
ENSG00000100292	HMOX1	heme oxygenase (decycling) 1	3162
ENSG00000196072	BLOC1S2	biogenesis of lysosomal organelles complex-1, subunit 2	282991
ENSG00000151929	BAG3	BCL2-associated athanogene 3	9531
ENSG00000135679	MDM2	Mdm2, p53 E3 ubiquitin protein ligase homolog (mouse)	4193
ENSG00000070669	ASNS	asparagine synthetase (glutamine-hydrolyzing)	440
ENSG00000161011	SQSTM1	sequestosome 1	8878
ENSG00000128016	ZFP36	zinc finger protein 36, C3H type, homolog (mouse)	7538
ENSG00000136560	TANK	TRAF family member-associated NFKB activator	10010
ENSG00000087088	BAX	BCL2-associated X protein	581
ENSG00000107796	ACTA2	actin, alpha 2, smooth muscle, aorta	59
ENSG00000080824	HSP90AA1	heat shock protein 90kDa alpha (cytosolic), class A member 1	3320

ENSG00000239713	APOBEC3G	apolipoprotein B mRNA editing enzyme, catalytic polypeptide-like 3G	60489
ENSG00000116761	CTH	cystathionase (cystathionine gamma-lyase)	1491
ENSG00000198431	TXNRD1	thioredoxin reductase 1	7296
ENSG00000087086	FTL	ferritin, light polypeptide	2512
ENSG00000142168	SOD1	superoxide dismutase 1, soluble	6647
ENSG00000106105	GARS	glycyl-tRNA synthetase	2617
ENSG00000135069	PSAT1	phosphoserine aminotransferase 1	29968
ENSG00000132170	PPARG	peroxisome proliferator-activated receptor gamma	5468
ENSG00000001084	GCLC	glutamate-cysteine ligase, catalytic subunit	2729
ENSG00000138413	IDH1	isocitrate dehydrogenase 1 (NADP+), soluble	3417
ENSG00000204388	HSPA1B	heat shock 70kDa protein 1B	3304
ENSG00000026103	FAS	Fas (TNF receptor superfamily, member 6)	355
ENSG00000121691	CAT	catalase	847
ENSG00000088356	PDRG1	p53 and DNA-damage regulated 1	81572
ENSG00000115919	KYNU	kynureninase	8942
ENSG00000087074	PPP1R15A	protein phosphatase 1, regulatory subunit 15A	23645
ENSG00000160211	G6PD	glucose-6-phosphate dehydrogenase	2539
ENSG00000105327	BBC3	BCL2 binding component 3	27113
ENSG00000106070	GRB10	growth factor receptor-bound protein 10	2887
ENSG00000120885	CLU	clusterin	1191
ENSG00000146733	PSPH	phosphoserine phosphatase	5723
ENSG00000136002	ARHGEF4	Rho guanine nucleotide exchange factor (GEF) 4	50649
**Cellular component**	**Intracellular organelle lumen**	**GO:0070013**	
C=3164;O=57;E=39.07;R=1.46;rawP=0.0013;adjP=0.0373			
ENSG00000166123	GPT2	glutamic pyruvate transaminase (alanine aminotransferase) 2	84706
ENSG00000120694	HSPH1	heat shock 105kDa/110kDa protein 1	10808
ENSG00000162616	DNAJB4	DnaJ (Hsp40) homolog, subfamily B, member 4	11080
ENSG00000120738	EGR1	early growth response 1	1958
ENSG00000221926	TRIM16	tripartite motif containing 16	10626
ENSG00000170485	NPAS2	neuronal PAS domain protein 2	4862
ENSG00000002587	HS3ST1	heparan sulfate (glucosamine) 3-O-sulfotransferase 1	9957
ENSG00000164938	TP53INP1	tumor protein p53 inducible nuclear protein 1	94241
ENSG00000102048	ASB9	ankyrin repeat and SOCS box containing 9	140462
ENSG00000160200	CBS	cystathionine-beta-synthase	875
ENSG00000132002	DNAJB1	DnaJ (Hsp40) homolog, subfamily B, member 1	3337
ENSG00000124762	CDKN1A	cyclin-dependent kinase inhibitor 1A (p21, Cip1)	1026
ENSG00000100292	HMOX1	heme oxygenase (decycling) 1	3162
ENSG00000139289	PHLDA1	pleckstrin homology-like domain, family A, member 1	22822
ENSG00000138166	DUSP5	dual specificity phosphatase 5	1847
ENSG00000169136	ATF5	activating transcription factor 5	22809
ENSG00000059728	MXD1	MAX dimerization protein 1	4084
ENSG00000048052	HDAC9	histone deacetylase 9	9734
ENSG00000085276	MECOM	MDS1 and EVI1 complex locus	2122

ENSG00000148700	ADD3	adducin 3 (gamma)	120
ENSG00000135679	MDM2	Mdm2, p53 E3 ubiquitin protein ligase homolog (mouse)	4193
ENSG00000161011	SQSTM1	sequestosome 1	8878
ENSG00000105993	DNAJB6	DnaJ (Hsp40) homolog, subfamily B, member 6	10049
ENSG00000161513	FDXR	ferredoxin reductase	2232
ENSG00000100889	PCK2	phosphoenolpyruvate carboxykinase 2 (mitochondrial)	5106
ENSG00000121931	LRIF1	ligand dependent nuclear receptor interacting factor 1	55791
ENSG00000063660	GPC1	glypican 1	2817
ENSG00000240694	PNMA2	paraneoplastic Ma antigen 2	10687
ENSG00000185591	SP1	Sp1 transcription factor	6667
ENSG00000198431	TXNRD1	thioredoxin reductase 1	7296
ENSG00000108179	PPIF	peptidylprolyl isomerase F	10105
ENSG00000173846	PLK3	polo-like kinase 3	1263
ENSG00000134574	DDB2	damage-specific DNA binding protein 2, 48kDa	1643
ENSG00000106105	GARS	glycyl-tRNA synthetase	2617
ENSG00000142168	SOD1	superoxide dismutase 1, soluble	6647
ENSG00000173614	NMNAT1	nicotinamide nucleotide adenylyltransferase 1	64802
ENSG00000132170	PPARG	peroxisome proliferator-activated receptor gamma	5468
ENSG00000153292	GPR110	G protein-coupled receptor 110	266977
ENSG00000115641	FHL2	four and a half LIM domains 2	2274
ENSG00000162772	ATF3	activating transcription factor 3	467
ENSG00000171617	ENC1	ectodermal-neural cortex 1 (with BTB-like domain)	8507
ENSG00000138413	IDH1	isocitrate dehydrogenase 1 (NADP+), soluble	3417
ENSG00000181222	POLR2A	polymerase (RNA) II (DNA directed) polypeptide A, 220kDa	5430
ENSG00000179583	CIITA	class II, major histocompatibility complex, transactivator	4261
ENSG00000204388	HSPA1B	heat shock 70kDa protein 1B	3304
ENSG00000135047	CTSL1	cathepsin L1	1514
ENSG00000121691	CAT	catalase	847
ENSG00000115919	KYNU	kynureninase	8942
ENSG00000023330	ALAS1	aminolevulinate, delta-, synthase 1	211
ENSG00000153879	CEBPG	CCAAT/enhancer binding protein (C/EBP), gamma	1054
ENSG00000181026	AEN	apoptosis enhancing nuclease	64782
ENSG00000101849	TBL1X	transducin (beta)-like 1X-linked	6907
ENSG00000170689	HOXB9	homeobox B9	3219
ENSG00000160570	DEDD2	death effector domain containing 2	162989
**Cellular component**	**Organelle lumen**	**GO:0043233**	
C=3209;O=58;E=39.63;R=1.46;rawP=0.0011;adjP=0.0373			
ENSG00000166123	GPT2	glutamic pyruvate transaminase (alanine aminotransferase) 2	84706
ENSG00000120694	HSPH1	heat shock 105kDa/110kDa protein 1	10808
ENSG00000162616	DNAJB4	DnaJ (Hsp40) homolog, subfamily B, member 4	11080
ENSG00000120738	EGR1	early growth response 1	1958
ENSG00000221926	TRIM16	tripartite motif containing 16	10626
ENSG00000170485	NPAS2	neuronal PAS domain protein 2	4862
ENSG00000002587	HS3ST1	heparan sulfate (glucosamine) 3-O-sulfotransferase 1	9957

ENSG00000164938	TP53INP1	tumor protein p53 inducible nuclear protein 1	94241
ENSG00000102048	ASB9	ankyrin repeat and SOCS box containing 9	140462
ENSG00000160200	CBS	cystathionine-beta-synthase	875
ENSG00000132002	DNAJB1	DnaJ (Hsp40) homolog, subfamily B, member 1	3337
ENSG00000124762	CDKN1A	cyclin-dependent kinase inhibitor 1A (p21, Cip1)	1026
ENSG00000100292	HMOX1	heme oxygenase (decycling) 1	3162
ENSG00000139289	PHLDA1	pleckstrin homology-like domain, family A, member 1	22822
ENSG00000138166	DUSP5	dual specificity phosphatase 5	1847
ENSG00000169136	ATF5	activating transcription factor 5	22809
ENSG00000059728	MXD1	MAX dimerization protein 1	4084
ENSG00000048052	HDAC9	histone deacetylase 9	9734
ENSG00000085276	MECOM	MDS1 and EVI1 complex locus	2122
ENSG00000148700	ADD3	adducin 3 (gamma)	120
ENSG00000135679	MDM2	Mdm2, p53 E3 ubiquitin protein ligase homolog (mouse)	4193
ENSG00000161011	SQSTM1	sequestosome 1	8878
ENSG00000105993	DNAJB6	DnaJ (Hsp40) homolog, subfamily B, member 6	10049
ENSG00000161513	FDXR	ferredoxin reductase	2232
ENSG00000100889	PCK2	phosphoenolpyruvate carboxykinase 2 (mitochondrial)	5106
ENSG00000121931	LRIF1	ligand dependent nuclear receptor interacting factor 1	55791
ENSG00000063660	GPC1	glypican 1	2817
ENSG00000240694	PNMA2	paraneoplastic Ma antigen 2	10687
ENSG00000185591	SP1	Sp1 transcription factor	6667
ENSG00000198431	TXNRD1	thioredoxin reductase 1	7296
ENSG00000108179	PPIF	peptidylprolyl isomerase F	10105
ENSG00000173846	PLK3	polo-like kinase 3	1263
ENSG00000134574	DDB2	damage-specific DNA binding protein 2, 48kDa	1643
ENSG00000106105	GARS	glycyl-tRNA synthetase	2617
ENSG00000142168	SOD1	superoxide dismutase 1, soluble	6647
ENSG00000173614	NMNAT1	nicotinamide nucleotide adenylyltransferase 1	64802
ENSG00000132170	PPARG	peroxisome proliferator-activated receptor gamma	5468
ENSG00000153292	GPR110	G protein-coupled receptor 110	266977
ENSG00000115641	FHL2	four and a half LIM domains 2	2274
ENSG00000162772	ATF3	activating transcription factor 3	467
ENSG00000171617	ENC1	ectodermal-neural cortex 1 (with BTB-like domain)	8507
ENSG00000138413	IDH1	isocitrate dehydrogenase 1 (NADP+), soluble	3417
ENSG00000181222	POLR2A	polymerase (RNA) II (DNA directed) polypeptide A, 220kDa	5430
ENSG00000179583	CIITA	class II, major histocompatibility complex, transactivator	4261
ENSG00000204388	HSPA1B	heat shock 70kDa protein 1B	3304
ENSG00000135047	CTSL1	cathepsin L1	1514
ENSG00000121691	CAT	catalase	847
ENSG00000115919	KYNU	kynureninase	8942
ENSG00000023330	ALAS1	aminolevulinate, delta-, synthase 1	211
ENSG00000153879	CEBPG	CCAAT/enhancer binding protein (C/EBP), gamma	1054
ENSG00000181026	AEN	apoptosis enhancing nuclease	64782
ENSG00000120885	CLU	clusterin	1191

ENSG00000101849	TBL1X	transducin (beta)-like 1X-linked	6907
ENSG00000170689	HOXB9	homeobox B9	3219
ENSG00000160570	DEDD2	death effector domain containing 2	162989
**Cellular component**	**Membrane-enclosed lumen**	**GO:0031974**	
C=3252;O=58;E=40.16;R=1.44;rawP=0.0015;adjP=0.0373			
ENSG00000166123	GPT2	glutamic pyruvate transaminase (alanine aminotransferase) 2	84706
ENSG00000120694	HSPH1	heat shock 105kDa/110kDa protein 1	10808
ENSG00000162616	DNAJB4	DnaJ (Hsp40) homolog, subfamily B, member 4	11080
ENSG00000120738	EGR1	early growth response 1	1958
ENSG00000221926	TRIM16	tripartite motif containing 16	10626
ENSG00000170485	NPAS2	neuronal PAS domain protein 2	4862
ENSG00000002587	HS3ST1	heparan sulfate (glucosamine) 3-O-sulfotransferase 1	9957
ENSG00000164938	TP53INP1	tumor protein p53 inducible nuclear protein 1	94241
ENSG00000102048	ASB9	ankyrin repeat and SOCS box containing 9	140462
ENSG00000160200	CBS	cystathionine-beta-synthase	875
ENSG00000132002	DNAJB1	DnaJ (Hsp40) homolog, subfamily B, member 1	3337
ENSG00000124762	CDKN1A	cyclin-dependent kinase inhibitor 1A (p21, Cip1)	1026
ENSG00000100292	HMOX1	heme oxygenase (decycling) 1	3162
ENSG00000139289	PHLDA1	pleckstrin homology-like domain, family A, member 1	22822
ENSG00000138166	DUSP5	dual specificity phosphatase 5	1847
ENSG00000169136	ATF5	activating transcription factor 5	22809
ENSG00000059728	MXD1	MAX dimerization protein 1	4084
ENSG00000048052	HDAC9	histone deacetylase 9	9734
ENSG00000085276	MECOM	MDS1 and EVI1 complex locus	2122
ENSG00000148700	ADD3	adducin 3 (gamma)	120
ENSG00000135679	MDM2	Mdm2, p53 E3 ubiquitin protein ligase homolog (mouse)	4193
ENSG00000161011	SQSTM1	sequestosome 1	8878
ENSG00000105993	DNAJB6	DnaJ (Hsp40) homolog, subfamily B, member 6	10049
ENSG00000161513	FDXR	ferredoxin reductase	2232
ENSG00000100889	PCK2	phosphoenolpyruvate carboxykinase 2 (mitochondrial)	5106
ENSG00000121931	LRIF1	ligand dependent nuclear receptor interacting factor 1	55791
ENSG00000063660	GPC1	glypican 1	2817
ENSG00000240694	PNMA2	paraneoplastic Ma antigen 2	10687
ENSG00000185591	SP1	Sp1 transcription factor	6667
ENSG00000198431	TXNRD1	thioredoxin reductase 1	7296
ENSG00000108179	PPIF	peptidylprolyl isomerase F	10105
ENSG00000173846	PLK3	polo-like kinase 3	1263
ENSG00000134574	DDB2	damage-specific DNA binding protein 2, 48kDa	1643
ENSG00000106105	GARS	glycyl-tRNA synthetase	2617
ENSG00000142168	SOD1	superoxide dismutase 1, soluble	6647
ENSG00000173614	NMNAT1	nicotinamide nucleotide adenylyltransferase 1	64802
ENSG00000132170	PPARG	peroxisome proliferator-activated receptor gamma	5468
ENSG00000153292	GPR110	G protein-coupled receptor 110	266977
ENSG00000115641	FHL2	four and a half LIM domains 2	2274

ENSG00000162772	ATF3	activating transcription factor 3	467
ENSG00000171617	ENC1	ectodermal-neural cortex 1 (with BTB-like domain)	8507
ENSG00000138413	IDH1	isocitrate dehydrogenase 1 (NADP+), soluble	3417
ENSG00000181222	POLR2A	polymerase (RNA) II (DNA directed) polypeptide A, 220kDa	5430
ENSG00000179583	CIITA	class II, major histocompatibility complex, transactivator	4261
ENSG00000204388	HSPA1B	heat shock 70kDa protein 1B	3304
ENSG00000135047	CTSL1	cathepsin L1	1514
ENSG00000121691	CAT	catalase	847
ENSG00000115919	KYNU	kynureninase	8942
ENSG00000153879	CEBPG	CCAAT/enhancer binding protein (C/EBP), gamma	1054
ENSG00000120885	CLU	clusterin	1191
ENSG00000170689	HOXB9	homeobox B9	3219
**Cellular component**	Nucleus	**GO:0005634**	
C=5563;O=88;E=68.70;R=1.28;rawP=0.0025;adjP=0.0544			
ENSG00000124496	TRERF1	transcriptional regulating factor 1	55809
ENSG00000091592	NLRP1	NLR family, pyrin domain containing 1	22861
ENSG00000134986	NREP	neuronal regeneration related protein homolog (rat)	9315
ENSG00000124762	CDKN1A	cyclin-dependent kinase inhibitor 1A (p21, Cip1)	1026
ENSG00000100292	HMOX1	heme oxygenase (decycling) 1	3162
ENSG00000139289	PHLDA1	pleckstrin homology-like domain, family A, member 1	22822
ENSG00000169136	ATF5	activating transcription factor 5	22809
ENSG00000059728	MXD1	MAX dimerization protein 1	4084
ENSG00000196072	BLOC1S2	biogenesis of lysosomal organelles complex-1, subunit 2	282991
ENSG00000135679	MDM2	Mdm2, p53 E3 ubiquitin protein ligase homolog (mouse)	4193
ENSG00000105993	DNAJB6	DnaJ (Hsp40) homolog, subfamily B, member 6	10049
ENSG00000128016	ZFP36	zinc finger protein 36, C3H type, homolog (mouse)	7538
ENSG00000158373	HIST1H2BD	histone cluster 1, H2bd	3017
ENSG00000239713	APOBEC3G	apolipoprotein B mRNA editing enzyme, catalytic polypeptide-like 3G	60489
ENSG00000116761	CTH	cystathionase (cystathionine gamma-lyase)	1491
ENSG00000146674	IGFBP3	insulin-like growth factor binding protein 3	3486
ENSG00000173846	PLK3	polo-like kinase 3	1263
ENSG00000142168	SOD1	superoxide dismutase 1, soluble	6647
ENSG00000130766	SESN2	sestrin 2	83667
ENSG00000185088	RPS27L	ribosomal protein S27-like	51065
ENSG00000132170	PPARG	peroxisome proliferator-activated receptor gamma	5468
ENSG00000153292	GPR110	G protein-coupled receptor 110	266977
ENSG00000197903	HIST1H2BK	histone cluster 1, H2bk	85236
ENSG00000162772	ATF3	activating transcription factor 3	467
ENSG00000171617	ENC1	ectodermal-neural cortex 1 (with BTB-like domain)	8507
ENSG00000080546	SESN1	sestrin 1	27244
ENSG00000147130	ZMYM3	zinc finger, MYM-type 3	9203
ENSG00000173110	HSPA6	heat shock 70kDa protein 6 (HSP70B')	3310
ENSG00000204390	HSPA1L	heat shock 70kDa protein 1-like	3305
ENSG00000115919	KYNU	kynureninase	8942

ENSG00000099860	GADD45B	growth arrest and DNA-damage-inducible, beta	4616
ENSG00000152137	HSPB8	heat shock 22kDa protein 8	26353
ENSG00000023330	ALAS1	aminolevulinate, delta-, synthase 1	211
ENSG00000134363	FST	follistatin	10468
ENSG00000120885	CLU	clusterin	1191
ENSG00000170689	HOXB9	homeobox B9	3219
ENSG00000120694	HSPH1	heat shock 105kDa/110kDa protein 1	10808
ENSG00000156273	BACH1	BTB and CNC homology 1, basic leucine zipper transcription factor 1	571
ENSG00000120738	EGR1	early growth response 1	1958
ENSG00000074590	NUAK1	NUAK family, SNF1-like kinase, 1	9891
ENSG00000164938	TP53INP1	tumor protein p53 inducible nuclear protein 1	94241
ENSG00000102048	ASB9	ankyrin repeat and SOCS box containing 9	140462
ENSG00000188158	NHS	Nance-Horan syndrome (congenital cataracts and dental anomalies)	4810
ENSG00000160200	CBS	cystathionine-beta-synthase	875
ENSG00000132002	DNAJB1	DnaJ (Hsp40) homolog, subfamily B, member 1	3337
ENSG00000138166	DUSP5	dual specificity phosphatase 5	1847
ENSG00000100625	SIX4	SIX homeobox 4	51804
ENSG00000048052	HDAC9	histone deacetylase 9	9734
ENSG00000085276	MECOM	MDS1 and EVI1 complex locus	2122
ENSG00000148700	ADD3	adducin 3 (gamma)	120
ENSG00000161011	SQSTM1	sequestosome 1	8878
ENSG00000087088	BAX	BCL2-associated X protein	581
ENSG00000072310	SREBF1	sterol regulatory element binding transcription factor 1	6720
ENSG00000116285	ERRFI1	ERBB receptor feedback inhibitor 1	54206
ENSG00000146453	PNLDC1	poly(A)-specific ribonuclease (PARN)-like domain containing 1	154197
ENSG00000121931	LRIF1	ligand dependent nuclear receptor interacting factor 1	55791
ENSG00000240694	PNMA2	paraneoplastic Ma antigen 2	10687
ENSG00000185591	SP1	Sp1 transcription factor	6667
ENSG00000198431	TXNRD1	thioredoxin reductase 1	7296
ENSG00000134574	DDB2	damage-specific DNA binding protein 2, 48kDa	1643
ENSG00000173614	NMNAT1	nicotinamide nucleotide adenylyltransferase 1	64802
ENSG00000115641	FHL2	four and a half LIM domains 2	2274
ENSG00000154556	SORBS2	sorbin and SH3 domain containing 2	8470
ENSG00000181222	POLR2A	polymerase (RNA) II (DNA directed) polypeptide A, 220kDa	5430
ENSG00000179583	CIITA	class II, major histocompatibility complex, transactivator	4261
ENSG00000204388	HSPA1B	heat shock 70kDa protein 1B	3304
ENSG00000026103	FAS	Fas (TNF receptor superfamily, member 6)	355
ENSG00000095383	TBC1D2	TBC1 domain family, member 2	55357
ENSG00000135047	CTSL1	cathepsin L1	1514
ENSG00000117318	ID3	inhibitor of DNA binding 3, dominant negative helix-loop-helix protein	3399
ENSG00000116717	GADD45A	growth arrest and DNA-damage-inducible, alpha	1647
ENSG00000153879	CEBPG	CCAAT/enhancer binding protein (C/EBP), gamma	1054
ENSG00000181026	AEN	apoptosis enhancing nuclease	64782

ENSG00000255150	EID3	EP300 interacting inhibitor of differentiation 3	493861
ENSG00000101849	TBL1X	transducin (beta)-like 1X-linked	6907
ENSG00000160570	DEDD2	death effector domain containing 2	162989
ENSG00000166166	TRMT61A	tRNA methyltransferase 61 homolog A (S. cerevisiae)	115708
ENSG00000105939	ZC3HAV1	zinc finger CCCH-type, antiviral 1	56829
**Cellular component**	**Nuclear part**	**GO:0044428**	
C=2948;O=52;E=36.40;R=1.43;rawP=0.0036;adjP=0.0696			
ENSG00000120694	HSPH1	heat shock 105kDa/110kDa protein 1	10808
ENSG00000162616	DNAJB4	DnaJ (Hsp40) homolog, subfamily B, member 4	11080
ENSG00000120738	EGR1	early growth response 1	1958
ENSG00000221926	TRIM16	tripartite motif containing 16	10626
ENSG00000170485	NPAS2	neuronal PAS domain protein 2	4862
ENSG00000164938	TP53INP1	tumor protein p53 inducible nuclear protein 1	94241
ENSG00000102048	ASB9	ankyrin repeat and SOCS box containing 9	140462
ENSG00000160200	CBS	cystathionine-beta-synthase	875
ENSG00000132002	DNAJB1	DnaJ (Hsp40) homolog, subfamily B, member 1	3337
ENSG00000124762	CDKN1A	cyclin-dependent kinase inhibitor 1A (p21, Cip1)	1026
ENSG00000100292	HMOX1	heme oxygenase (decycling) 1	3162
ENSG00000139289	PHLDA1	pleckstrin homology-like domain, family A, member 1	22822
ENSG00000138166	DUSP5	dual specificity phosphatase 5	1847
ENSG00000169136	ATF5	activating transcription factor 5	22809
ENSG00000059728	MXD1	MAX dimerization protein 1	4084
ENSG00000048052	HDAC9	histone deacetylase 9	9734
ENSG00000085276	MECOM	MDS1 and EVI1 complex locus	2122
ENSG00000160117	ANKLE1	ankyrin repeat and LEM domain containing 1	126549
ENSG00000148700	ADD3	adducin 3 (gamma)	120
ENSG00000135679	MDM2	Mdm2, p53 E3 ubiquitin protein ligase homolog (mouse)	4193
ENSG00000161011	SQSTM1	sequestosome 1	8878
ENSG00000105993	DNAJB6	DnaJ (Hsp40) homolog, subfamily B, member 6	10049
ENSG00000072310	SREBF1	sterol regulatory element binding transcription factor 1	6720
ENSG00000121931	LRIF1	ligand dependent nuclear receptor interacting factor 1	55791
ENSG00000239713	APOBEC3G	apolipoprotein B mRNA editing enzyme, catalytic polypeptide-like 3G	60489
ENSG00000240694	PNMA2	paraneoplastic Ma antigen 2	10687
ENSG00000185591	SP1	Sp1 transcription factor	6667
ENSG00000198431	TXNRD1	thioredoxin reductase 1	7296
ENSG00000173846	PLK3	polo-like kinase 3	1263
ENSG00000134574	DDB2	damage-specific DNA binding protein 2, 48kDa	1643
ENSG00000142168	SOD1	superoxide dismutase 1, soluble	6647
ENSG00000173614	NMNAT1	nicotinamide nucleotide adenylyltransferase 1	64802
ENSG00000132170	PPARG	peroxisome proliferator-activated receptor gamma	5468
ENSG00000153292	GPR110	G protein-coupled receptor 110	266977
ENSG00000115641	FHL2	four and a half LIM domains 2	2274
ENSG00000162772	ATF3	activating transcription factor 3	467
ENSG00000171617	ENC1	ectodermal-neural cortex 1 (with BTB-like domain)	8507

ENSG00000181222	POLR2A	polymerase (RNA) II (DNA directed) polypeptide A, 220kDa	5430
ENSG00000179583	CIITA	class II, major histocompatibility complex, transactivator	4261
ENSG00000204388	HSPA1B	heat shock 70kDa protein 1B	3304
ENSG00000173110	HSPA6	heat shock 70kDa protein 6 (HSP70B')	3310
ENSG00000204390	HSPA1L	heat shock 70kDa protein 1-like	3305
ENSG00000115919	KYNU	kynureninase	8942
ENSG00000023330	ALAS1	aminolevulinate, delta-, synthase 1	211
ENSG00000153879	CEBPG	CCAAT/enhancer binding protein (C/EBP), gamma	1054
ENSG00000181026	AEN	apoptosis enhancing nuclease	64782
ENSG00000101849	TBL1X	transducin (beta)-like 1X-linked	6907
ENSG00000170689	HOXB9	homeobox B9	3219
ENSG00000160570	DEDD2	death effector domain containing 2	162989
**Cellular component**	**Nuclear lumen**	**GO:0031981**	
C=2632;O=47;E=32.50;R=1.45;rawP=0.0047;adjP=0.0818			
ENSG00000120694	HSPH1	heat shock 105kDa/110kDa protein 1	10808
ENSG00000162616	DNAJB4	DnaJ (Hsp40) homolog, subfamily B, member 4	11080
ENSG00000120738	EGR1	early growth response 1	1958
ENSG00000221926	TRIM16	tripartite motif containing 16	10626
ENSG00000170485	NPAS2	neuronal PAS domain protein 2	4862
ENSG00000164938	TP53INP1	tumor protein p53 inducible nuclear protein 1	94241
ENSG00000102048	ASB9	ankyrin repeat and SOCS box containing 9	140462
ENSG00000160200	CBS	cystathionine-beta-synthase	875
ENSG00000132002	DNAJB1	DnaJ (Hsp40) homolog, subfamily B, member 1	3337
ENSG00000124762	CDKN1A	cyclin-dependent kinase inhibitor 1A (p21, Cip1)	1026
ENSG00000100292	HMOX1	heme oxygenase (decycling) 1	3162
ENSG00000139289	PHLDA1	pleckstrin homology-like domain, family A, member 1	22822
ENSG00000138166	DUSP5	dual specificity phosphatase 5	1847
ENSG00000169136	ATF5	activating transcription factor 5	22809
ENSG00000059728	MXD1	MAX dimerization protein 1	4084
ENSG00000048052	HDAC9	histone deacetylase 9	9734
ENSG00000085276	MECOM	MDS1 and EVI1 complex locus	2122
ENSG00000148700	ADD3	adducin 3 (gamma)	120
ENSG00000135679	MDM2	Mdm2, p53 E3 ubiquitin protein ligase homolog (mouse)	4193
ENSG00000161011	SQSTM1	sequestosome 1	8878
ENSG00000105993	DNAJB6	DnaJ (Hsp40) homolog, subfamily B, member 6	10049
ENSG00000121931	LRIF1	ligand dependent nuclear receptor interacting factor 1	55791
ENSG00000240694	PNMA2	paraneoplastic Ma antigen 2	10687
ENSG00000185591	SP1	Sp1 transcription factor	6667
ENSG00000198431	TXNRD1	thioredoxin reductase 1	7296
ENSG00000173846	PLK3	polo-like kinase 3	1263
ENSG00000134574	DDB2	damage-specific DNA binding protein 2, 48kDa	1643
ENSG00000142168	SOD1	superoxide dismutase 1, soluble	6647
ENSG00000173614	NMNAT1	nicotinamide nucleotide adenylyltransferase 1	64802
ENSG00000132170	PPARG	peroxisome proliferator-activated receptor gamma	5468
ENSG00000153292	GPR110	G protein-coupled receptor 110	266977

ENSG00000115641	FHL2	four and a half LIM domains 2	2274
ENSG00000162772	ATF3	activating transcription factor 3	467
ENSG00000171617	ENC1	ectodermal-neural cortex 1 (with BTB-like domain)	8507
ENSG00000181222	POLR2A	polymerase (RNA) II (DNA directed) polypeptide A, 220kDa	5430
ENSG00000179583	CIITA	class II, major histocompatibility complex, transactivator	4261
ENSG00000204388	HSPA1B	heat shock 70kDa protein 1B	3304
ENSG00000115919	KYNU	kynureninase	8942
ENSG00000023330	ALAS1	aminolevulinate, delta-, synthase 1	211
ENSG00000153879	CEBPG	CCAAT/enhancer binding protein (C/EBP), gamma	1054
ENSG00000181026	AEN	apoptosis enhancing nuclease	64782
ENSG00000101849	TBL1X	transducin (beta)-like 1X-linked	6907
ENSG00000170689	HOXB9	homeobox B9	3219
ENSG00000160570	DEDD2	death effector domain containing 2	162989

User file and parameters: User file: textAreaUpload.txt, Organism: hsapiens, Id Type: ensembl_gene_stable_id, Ref Set: illumina_humanref_8_v2, Significance Level: Top10, Statistics Test: Hypergeometric, MTC: BH

For each GO category, the first row lists its sub-root (biological process, molecular function, or cellular component), category name, and corresponding GO ID.

The second row lists number of reference genes in the category (C), number of genes in the gene set and also in the category (O), expected number in the category (E), Ratio of enrichment (R), p value from hypergeometric test

Table S3. String network analysis of DEGs. Browse interactions in tabular form.

#node1	node2	node1_string_internal_id	node2_string_internal_id	node1_external_id	node2_external_id	homology	experimentally determined interaction	database annotated	combineds core
**6h post SFN treatment**									
SQSTM1	MAP1LC3B	1857860	1846025	9606.ENSP00000374455	9606.ENSP00000268607	0	0,999	0	0,999
GCLM	GCLC	1855202	1843208	9606.ENSP00000359258	9606.ENSP00000229416	0	0,978	0,9	0,997
HSP90AA1	HSPA6	1851814	1849273	9606.ENSP00000335153	9606.ENSP00000310219	0	0,901	0,9	0,989
GARS	ASNS	1857811	1842325	9606.ENSP00000373918	9606.ENSP00000175506	0	0,986	0	0,986
HSP90AA1	HSPH1	1851814	1850070	9606.ENSP00000335153	9606.ENSP00000318687	0	0,677	0,9	0,966
DNAJB6	DNAJB1	1845081	1844287	9606.ENSP00000262177	9606.ENSP00000254322	0,701	0,32	0,9	0,929
HSPA6	DNAJB1	1849273	1844287	9606.ENSP00000310219	9606.ENSP00000254322	0	0,301	0,9	0,927
HSPH1	DNAJB1	1850070	1844287	9606.ENSP00000318687	9606.ENSP00000254322	0	0,255	0,9	0,922
HSPH1	DNAJB6	1850070	1845081	9606.ENSP00000318687	9606.ENSP00000262177	0	0,235	0,9	0,92
HSPA6	DNAJB6	1849273	1845081	9606.ENSP00000310219	9606.ENSP00000262177	0	0,235	0,9	0,92
HSPH1	HSPA6	1850070	1849273	9606.ENSP00000318687	9606.ENSP00000310219	0,786	0,211	0,9	0,917
HSP90AA1	DNAJB6	1851814	1845081	9606.ENSP00000335153	9606.ENSP00000262177	0	0,168	0,9	0,913
HSP90AA1	DNAJB1	1851814	1844287	9606.ENSP00000335153	9606.ENSP00000254322	0	0,168	0,9	0,913
MTHFD2	ALDH1L2	1858157	1844615	9606.ENSP00000377617	9606.ENSP00000258494	0	0,074	0,9	0,903
DEDD2	HSPA6	1864303	1849273	9606.ENSP00000470082	9606.ENSP00000310219	0	0	0,9	0,9
DEDD2	HSPH1	1864303	1850070	9606.ENSP00000470082	9606.ENSP00000318687	0	0	0,9	0,9
DEDD2	DNAJB1	1864303	1844287	9606.ENSP00000470082	9606.ENSP00000254322	0	0	0,9	0,9
DEDD2	HSP90AA1	1864303	1851814	9606.ENSP00000470082	9606.ENSP00000335153	0	0	0,9	0,9
DEDD2	DNAJB6	1864303	1845081	9606.ENSP00000470082	9606.ENSP00000262177	0	0	0,9	0,9
LPAR1	F2R	1853763	1850333	9606.ENSP00000351755	9606.ENSP00000321326	0	0	0,9	0,899
LPAR1	ADORA1	1853763	1849083	9606.ENSP00000351755	9606.ENSP00000308549	0,686	0	0,9	0,899
LPAR3	F2R	1855308	1850333	9606.ENSP00000359643	9606.ENSP00000321326	0	0	0,9	0,899
PSAT1	PSPH	1856523	1846450	9606.ENSP00000365773	9606.ENSP00000275605	0	0	0,9	0,899
TXNRD1	CTH	1861975	1855373	9606.ENSP00000434516	9606.ENSP00000359976	0	0	0,9	0,899
LPAR3	LPAR1	1855308	1853763	9606.ENSP00000359643	9606.ENSP00000351755	0,949	0	0,9	0,899
LPAR3	ADORA1	1855308	1849083	9606.ENSP00000359643	9606.ENSP00000308549	0,635	0	0,9	0,899
DNAJA1	HSP90AA1	1857095	1851814	9606.ENSP00000369127	9606.ENSP00000335153	0	0,886	0	0,886
BAG3	HSPB8	1854947	1846754	9606.ENSP00000358081	9606.ENSP00000281938	0	0,866	0	0,865
HSP90AA1	CHORDC1	1851814	1850131	9606.ENSP00000335153	9606.ENSP00000319255	0	0,815	0	0,815
HSPA1A	HSP90AA1	1856351	1851814	9606.ENSP00000364802	9606.ENSP00000335153	0	0,812	0	0,812
HSPA1L	HSP90AA1	1856352	1851814	9606.ENSP00000364805	9606.ENSP00000335153	0	0,812	0	0,812
HSPA1B	HSP90AA1	1856350	1851814	9606.ENSP00000364801	9606.ENSP00000335153	0	0,812	0	0,812
SQSTM1	SESN2	1857860	1844194	9606.ENSP00000374455	9606.ENSP00000253063	0	0,804	0	0,804
CTH	GCLC	1855373	1843208	9606.ENSP00000359976	9606.ENSP00000229416	0	0	0,8	0,8
CTH	GCLM	1855373	1855202	9606.ENSP00000359976	9606.ENSP00000359258	0	0	0,8	0,8
SQSTM1	BAG3	1857860	1854947	9606.ENSP00000374455	9606.ENSP00000358081	0	0,706	0	0,706
HSPA1A	DNAJB1	1856351	1844287	9606.ENSP00000364802	9606.ENSP00000254322	0	0,69	0	0,69
HSPA1A	BAG3	1856351	1854947	9606.ENSP00000364802	9606.ENSP00000358081	0	0,685	0	0,685
SQSTM1	HSPA6	1857860	1849273	9606.ENSP00000374455	9606.ENSP00000310219	0	0,57	0	0,569
DNAJB1	ZFP36	1844287	1843925	9606.ENSP00000254322	9606.ENSP00000248673	0	0,567	0	0,567
LRIF1	PPARG	1855096	1847090	9606.ENSP00000358778	9606.ENSP00000287820	0	0,567	0	0,567
HSPA1A	PPP1R15A	1856351	1842391	9606.ENSP00000364802	9606.ENSP00000200453	0	0,564	0	0,564
DNAJA1	DNAJB1	1857095	1844287	9606.ENSP00000369127	9606.ENSP00000254322	0,818	0,506	0	0,506
DNAJA1	DNAJB4	1857095	1855345	9606.ENSP00000369127	9606.ENSP00000359799	0,827	0,506	0	0,506
BAG3	HSPA6	1854947	1849273	9606.ENSP00000358081	9606.ENSP00000310219	0	0,469	0	0,469
DNAJB4	HSPH1	1855345	1850070	9606.ENSP00000359799	9606.ENSP00000318687	0	0,409	0	0,409
**12h post SFN treatment**									
FTL	FTH1	1856660	1846327	9606.ENSP00000366525	9606.ENSP00000273550	0,965	0,994	0,9	0,999
FST	INHBA	1844467	1843613	9606.ENSP00000256759	9606.ENSP00000242208	0	0,98	0,8	0,995
**24h post SFN treatment**									
MDM2	CDKN1A	1861282	1843736	9606.ENSP00000417281	9606.ENSP00000244741	0	0,997	0	0,996
MDM2	BBC3	1861282	1860479	9606.ENSP00000417281	9606.ENSP00000404503	0	0,983	0	0,982
CLU	BAX	1849761	1847402	9606.ENSP00000315130	9606.ENSP00000293288	0	0,982	0	0,982
HOXB9	BTG2	1849176	1847240	9606.ENSP00000309439	9606.ENSP00000290551	0	0,576	0,9	0,955
GADD45A	GADD45B	1855383	1842495	9606.ENSP00000360025	9606.ENSP00000215631	0,96	0,576	0,9	0,955
SP1	CDKN1A	1851138	1843736	9606.ENSP00000329357	9606.ENSP00000244741	0	0,567	0,9	0,954
NPAS2	TBL1X	1852092	1842681	9606.ENSP00000338283	9606.ENSP00000217964	0	0,545	0,9	0,952
GADD45A	CDKN1A	1855383	1843736	9606.ENSP00000360025	9606.ENSP00000244741	0	0,915	0	0,915
HSPH1	TNFRSF21	1850070	1847799	9606.ENSP00000318687	9606.ENSP00000296861	0	0	0,9	0,9
TXNRD1	TBL1X	1861975	1842681	9606.ENSP00000434516	9606.ENSP00000217964	0	0	0,9	0,9
TNFRSF21	TBL1X	1847799	1842681	9606.ENSP00000296861	9606.ENSP00000217964	0	0	0,9	0,9
ITGB2	PLAT	1848511	1842802	9606.ENSP00000303242	9606.ENSP00000220809	0	0	0,9	0,9
ALAS1	TBL1X	1849161	1842681	9606.ENSP00000309259	9606.ENSP00000217964	0	0	0,9	0,9
SP1	TBL1X	1851138	1842681	9606.ENSP00000329357	9606.ENSP00000217964	0	0	0,9	0,9
SOD1	CAT	1846132	1843587	9606.ENSP00000270142	9606.ENSP00000241052	0	0	0,9	0,899
FHL2	TBL1X	1850494	1842681	9606.ENSP00000322909	9606.ENSP00000217964	0	0	0,9	0,899
BBC3	SP1	1860479	1851138	9606.ENSP00000404503	9606.ENSP00000329357	0	0	0,9	0,899
SP1	BAX	1851138	1847402	9606.ENSP00000329357	9606.ENSP00000293288	0	0	0,9	0,899
HIST1H2BK	HIST1H2BD	1853439	1847163	9606.ENSP00000349430	9606.ENSP00000289316	0,988	0	0,9	0,899

MDM2	ATF3	1861282	1852803	9606.ENSP00000417281	9606.ENSP00000344352	0	0,812	0	0,812
SP1	EGR1	1851138	1843543	9606.ENSP00000329357	9606.ENSP00000239938	0,646	0,807	0	0,806
IL7R	IL11	1848815	1845567	9606.ENSP00000306157	9606.ENSP00000264563	0	0	0,8	0,8
SREBF1	SP1	1853255	1851138	9606.ENSP00000348069	9606.ENSP00000329357	0	0,798	0	0,798
POLR2A	CDKN1A	1849743	1843736	9606.ENSP00000314949	9606.ENSP00000244741	0	0,723	0	0,722
ITGB2	ITGB4	1848511	1842390	9606.ENSP00000303242	9606.ENSP00000200181	0,819	0	0,72	0,72
SQSTM1	BAG3	1857860	1854947	9606.ENSP00000374455	9606.ENSP00000358081	0	0,706	0	0,706
FAS	TNFRSF10B	1853242	1846494	9606.ENSP00000347979	9606.ENSP00000276431	0,636	0,667	0	0,667
SQSTM1	SOD1	1857860	1846132	9606.ENSP00000374455	9606.ENSP00000270142	0	0,654	0	0,654
IGFBP3	CTSL1	1857331	1852924	9606.ENSP00000370473	9606.ENSP00000345344	0	0,576	0	0,575
DDB2	CDKN1A	1844481	1843736	9606.ENSP00000256996	9606.ENSP00000244741	0	0,576	0	0,575
CDKN1A	GADD45B	1843736	1842495	9606.ENSP00000244741	9606.ENSP00000215631	0	0,576	0	0,575
ID3	SREBF1	1856143	1853255	9606.ENSP00000363689	9606.ENSP00000348069	0	0,576	0	0,575
MDM2	EGR1	1861282	1843543	9606.ENSP00000417281	9606.ENSP00000239938	0	0,576	0	0,575
BAG3	SOD1	1854947	1846132	9606.ENSP00000358081	9606.ENSP00000270142	0	0,573	0	0,573
FHL2	ITGB2	1850494	1848511	9606.ENSP00000322909	9606.ENSP00000303242	0	0,57	0	0,569
BBC3	BAX	1860479	1847402	9606.ENSP00000404503	9606.ENSP00000293288	0	0,57	0	0,569
SESN1	SQSTM1	1859742	1857860	9606.ENSP00000393762	9606.ENSP00000374455	0	0,567	0	0,567
SREBF1	INSIG1	1853255	1852851	9606.ENSP00000348069	9606.ENSP00000344741	0	0,567	0	0,567
SQSTM1	FAS	1857860	1853242	9606.ENSP00000374455	9606.ENSP00000347979	0	0,564	0	0,564
MDM2	GADD45A	1861282	1855383	9606.ENSP00000417281	9606.ENSP00000360025	0	0,564	0	0,563
MDM2	RPS27L	1861282	1851302	9606.ENSP00000417281	9606.ENSP00000331019	0	0,564	0	0,563

Table S4. List of primers used for the qPCR

Target gene	Forward primer (5′ to 3′)	Reverse primer (5′ to 3′)
**GAPDH**	GGAGTCAACGGATTTGGTC	GGCAACAATATCCACTTTACC
**NQO1**	TCTATGCCATGAACTTCAATCC	CTTCAGTTTACCTGTGATGTC
**HO-1**	AAAGTGCAAGATTCTGCCC	GAGTGTAAGGACCCATCGG
**GCLM**	TCTGGAAACTCCCTGACC	CCTTGAATGAATGGAGTTCCC
**GCLC**	AAACCCAAACCATCCTACC	AATATAGAAGTAGCCTCCTTCC
**TXNRD1**	AGACTCTCGAAATTATGGATGG	CCCAATCATGCTTAACTGTCTC
**GADD45b**	ACATCGCCCTGCAAATCC	ACACCCGCACGATGTTGAT
**CDKN1A**	GTCTTGTACCCTTGTGCC	GGTAGAAATCTGTCATGCTGG
**ATF3**	CTGCAGAAAGAGTCGGAG	CTGGAGTCCTCCCATTCTG
**BAX**	GACGGCAACTTCAACTGG	GAAGTCCAATGTCCAGCC
**EGR1**	CAGAAGGACAAGAAAGCAGAC	GGATGGGTATGAGGTGGT
**FAS**	CATAAGCCCTGTCCTCCA	GTCTGTGTACTCCTTCCCT
**HSPA1A**	GCCAACAAGATCACCATCAC	GTCCTCCGCTTTGTACTTCTC
**ITGB4**	GTATTGCGACTATGAGATGAAGG	GGAGGAGACGACATTGAAGG
